# Photocatalyzed Functionalization of Alkenoic Acids in 3D‐Printed Reactors

**DOI:** 10.1002/cssc.202200898

**Published:** 2022-07-08

**Authors:** Alexandra Jorea, Davide Ravelli, Rodrigo M. Romarowski, Stefania Marconi, Ferdinando Auricchio, Maurizio Fagnoni

**Affiliations:** ^1^ Department of Clinical Surgery, Diagnostics and Pediatrics Fondazione IRCCS Policlinico San Matteo Viale Brambilla 74 27100 Pavia Italy; ^2^ PhotoGreen Lab, Department of Chemistry University of Pavia Viale Taramelli 12 27100 Pavia Italy; ^3^ Computational Mechanics and Advanced Materials Group University of Pavia Via Ferrata 3 27100 Pavia Italy

**Keywords:** carboxylic acids, 3D-printed reactors, flow chemistry, photocatalysis, radical reactions

## Abstract

The valorization of alkenoic acids possibly deriving from biomass (fumaric and citraconic acids) was carried out through conversion in important building blocks, such as γ‐keto acids and succinic acid derivatives. The functionalization was carried out by addition onto the C=C double bond of radicals generated under photocatalyzed conditions from suitable hydrogen donors (mainly aldehydes) and by adopting a decatungstate salt as the photocatalyst. Syntheses were performed under batch (in a glass vessel) and flow (by using 3D‐printed reactors) conditions. The design of the latter reactors allowed for an improved yield and productivity.

## Graphical abstract

**Biomass valorization**: Alkenoic acids were smoothly converted into valuable γ‐keto acids and succinic acid derivatives by decatungstate photocatalyzed addition of selected hydrogen donors (e.g. aldehydes). The adoption of flow conditions and of a 3D‐printed reactor allowed to form the derivatized products in up to quantitative yield with a productivity of ≈15 mmol day^−1^.

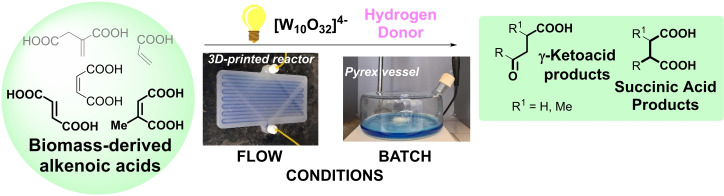

## Introduction

Chemical industry is nowadays largely based on fossil resources, which causes global environmental problems, notably increasing pollution levels and rising temperatures. Accordingly, there is an urgent need to move towards more sustainable ways to provide humankind with the materials and products needed to satisfy their needs. While having recourse to renewable raw materials is currently considered an intriguing alternative, the transition towards this class of building blocks will be compulsory in the near future, due to the rapid depletion of fossil‐based resources. Thus, biomass (not in competition with the food supply chain) will represent the election choice as inexhaustible source of a wide range of value‐added products, including platform chemicals and fuels.[^1^, ^2^, ^3^, ^4^, ^5^, ^6^] Thermochemical catalysis and microbial fermentation are the main approaches available to reutilize non‐edible lignocellulosic agricultural residues or sugar wastes.^[7]^

Organic acids are, among the plethora of organic building blocks derived from biomass, one of the most interesting classes, since they find application for the synthesis of important polymers, notably polyesters and polyamides.^[8]^ Moreover, biomass‐derived organic acids often contain further functional groups (e.g., an unsaturation), and several alkenoic acids can be easily accessed in large amounts by synthetic routes competitive to those employing fossil‐based starting materials.

**Scheme 1:**
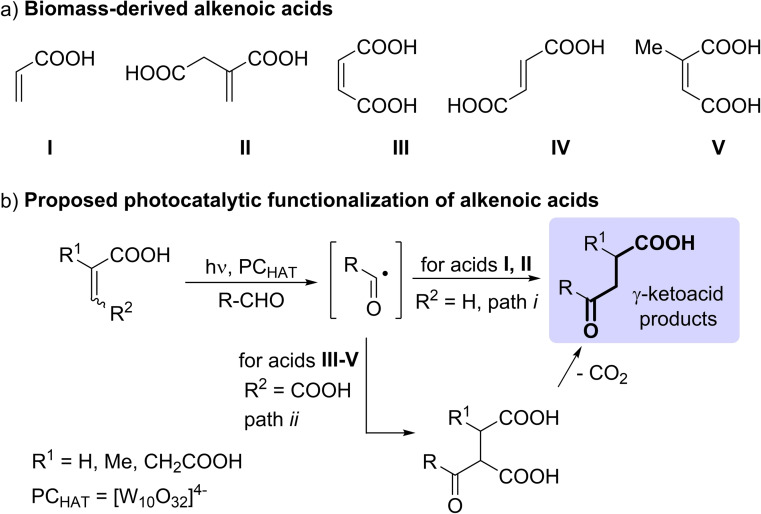
a) Biomass‐derived alkenoic acids tested in this work. b) Decatungstate photocatalyzed addition of aldehydes onto alkenoic acids.

As an example, acrylic acid (**I**, Scheme 1a) is commonly formed through the catalytic oxidation of propylene, but valuable alternative routes allow its generation from biomass‐deriving allyl alcohol or fumaric/maleic acids.[^9^, ^10^, ^11^] One of the most important alkenoic acids from biomass is undoubtedly itaconic acid (**II**), chemically prepared by pyrolysis of citric acid and subsequent hydrolysis of the thus formed itaconic anhydride. However, this priority building block may be formed by microbial fermentation in the frame of industrial biomass conversion.[^12^, ^13^, ^14^, ^15^, ^16^, ^17^, ^18^, ^19^] Maleic acid (**III**), usually formed by non‐renewable sources from maleic anhydride produced through catalytic oxidation of benzene or other hydrocarbons (e.g. butane) is well known to be derived from lignocellulosic agroresidues or from furfural.[^20^, ^21^, ^22^] Fumaric acid (**IV**), a naturally occurring key intermediate, is currently obtained by isomerization of maleic acid. However, this acid may be also obtained by fungal fermentation (employing *Rhizopus* strains) of starch‐containing materials, glucose, xylose and lignocellulosic derivatives.[^23^, ^24^, ^25^, ^26^, ^27^] Another underutilized alkenoic acid is citraconic acid (**V**), mostly formed by isomerization of itaconic acid.[^28^, ^29^, ^30^, ^31^, ^32^]

Unsaturated acids **I**–**V** are important in view of the possible derivatization of the C=C double bond to forge new C−C bonds. One of the most eco‐sustainable approaches for the derivatization of biomass‐derived compounds is through the implementation of photochemical/photocatalyzed strategies,^[33]^ making use of traceless photons.[^34^, ^35^, ^36^]

Recently, we demonstrated that bio‐crotonic acid may be formed by depolymerization of polyhydroxybutyrate (PHB) and easily acylated under photocatalytic conditions.^[37]^ The decatungstate anion [W_10_O_32_]^4−^ functioned here as photocatalyst (PC_HAT_) for the generation of acyl radicals by direct hydrogen atom transfer (HAT) from aldehydes.[^38^, ^39^]

We then envisioned that our approach could be extended to the derivatization of other bio‐based alkenoic acids for the synthesis of lead compounds. At the onset of this project, we focused our attention on the preparation of γ‐keto acids, important building blocks on the route to novel thiazole pyridazinone derivatives (having anti‐convulsant activity)^[40]^ and other heterocycles showing antimicrobial activity.^[41]^ The proposed strategy is depicted in Scheme 1b and involves the radical C(sp^2^)−H functionalization of aldehydes by using acrylic and itaconic acids as alkenoic acids (path *i*).

## Results and Discussion

We first tested the acylation of acrylic acid **I** by using benzaldehyde as model acylating agent. TBADT (tetrabutylammonium decatungstate, (*n*Bu_4_N)_4_[W_10_O_32_])[^38^, ^39^] was used as the PC_HAT_ (2 mol %) upon irradiation at 370 nm with a 40 W LED lamp in aqueous acetonitrile (MeCN : H_2_O 9 : 1). The reactions have been performed batch‐wise in a glass vessel (see Figure S3 in the Supporting Information). Unfortunately, the acylation product 4‐oxo‐4‐phenylbutanoic acid was isolated with a poor mass balance (13 % yield of isolated product) and a white precipitate sticking to the vessel walls was consistently formed, pointing to the possible occurrence of a polymerization event. Itaconic acid **II** was next tested, but the photocatalyzed reaction with benzaldehyde disappointingly failed to give significant amounts of the desired adduct (the GC‐MS analysis of the irradiated mixture is shown in Figure S8).

A strategic change was thus required in our approach. We envisaged that, due to the easy decarboxylation occurring in β‐keto acids, the acylation of maleic/fumaric acids **III**/**IV**, followed by carbon dioxide loss, could give the same adduct accessible from acrylic acid (Scheme 1b, path *ii*). We were confident on the success of this idea, since we previously observed a spontaneous decarboxylation of acetylsuccinic acid (to give levulinic acid) in the photocatalyzed acetylation of maleic anhydride.^[42]^ We then compared the performance of maleic and fumaric acids in the preparation of 4‐oxo‐4‐phenylbutanoic acid by using benzaldehyde as the acylating agent, while maintaining the same conditions and setup used for **I**/**II**. Notably, the adoption of these dicarboxylic acids led to consistently higher mass balances, the hoped for acylated derivative **3 a** being formed in a better yield by using fumaric acid (81 %) rather than maleic acid (60 %).

**Scheme 2:**
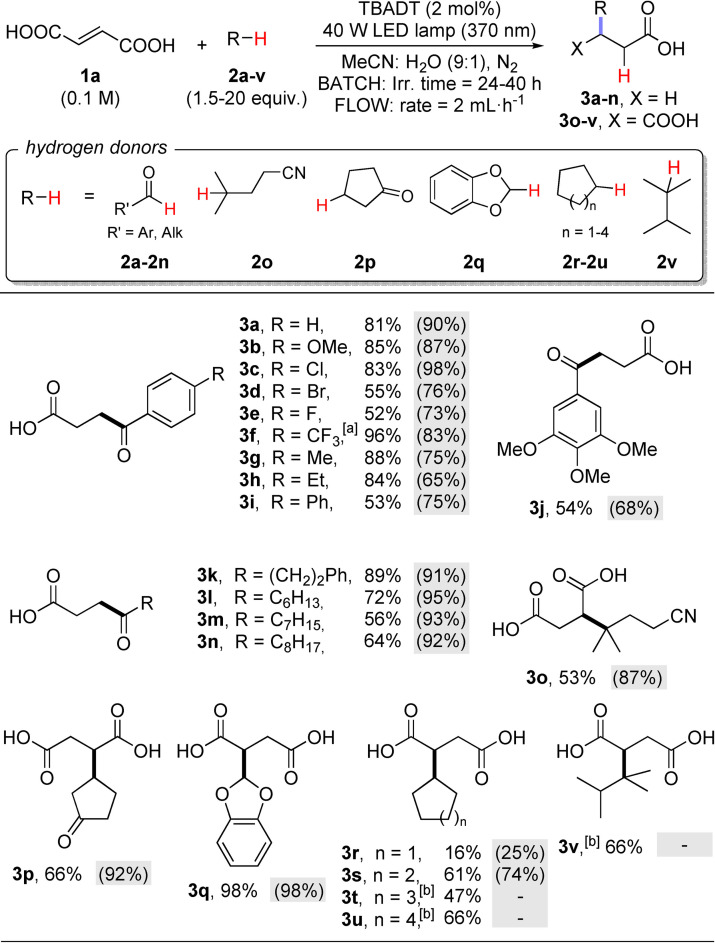
Photocatalyzed functionalization of fumaric acid **1 a**. Isolated yields for reactions performed under batch (2.5 mmol scale) or flow conditions (3D‐printed Reactor A–1.0 mmol scale; figures within parentheses on gray background). Typical reaction conditions: A solution of fumaric acid **1 a** (0.1 m), hydrogen donors **2 a**–**v** (0.15–2 m) and TBADT (2 mol%) in a MeCN : H_2_O 9 : 1 mixture was deaerated by argon bubbling for 10 min and irradiated using a 370 nm LED lamp (see Supporting Information for details). [a] Irradiation performed at 390 nm. [b] Reaction medium: MeCN/CH_2_Cl_2_ 5 : 1 mixture; reaction not performed under flow conditions due to incompatibility issues with the reactor material.

Following these preliminary experiments, the derivatization of fumaric acid (**1 a**, Scheme 2) was then explored by using aromatic aldehydes **2 a**–**2 j** due to the importance of the resulting γ‐keto acids.[^40^, ^41^] The experiments carried out under batch conditions (Pyrex vessel containing 25 mL of solution were used, exposed surface: ≈95 cm^2^; Figure S3) were also compared in most cases with those performed under flow conditions.[^43^, ^44^] Based on literature precedents,^[45]^ we adopted a 3D‐printed reactor made of polypropylene (Reactor A; dimensions: 70 mm×136.5 mm, excluding connectors; exposed surface: ≈96 cm^2^, height: 6 mm), with 1 mm channels, for a total volume of 4.1 mL. The inner channels are surrounded by 2.5 mm of polypropylene on top and bottom (Figures S1 and S2). In this case, the reaction mixture was charged in an appropriate reservoir loop (1 mm PTFE tubing) and circulated through the reactor by means of a syringe pump with a flow rate of 2 mL h^−1^ (Figures S4 and S5). The connection to the reactor inflow and outflow is made through two female luer lock connectors (Figure S1).

Gratifyingly, γ‐keto acids **3 a**–**3 j** resulting from the decarboxylative acylation of **1 a** were formed in good to excellent yields (52 % to 96 %) under batch conditions, quite independently from the aromatic substituents. In the synthesis of **3 f**, it was more convenient to adopt 390 nm light irradiation to promote the desired acylation. The derivatizations performed under flow conditions consistently delivered a higher yield (up to 98 %), except for products **3 f**–**3 h**. We then found that aliphatic aldehydes **2 k**–**2 n** were likewise suitable substrates for the acylation of **1 a** to give the corresponding γ‐keto acids **3 k**–**3 n**. Even with these derivatives, shifting to flow conditions improved the acylation yields (consistently >90 %, Scheme 2).

Inspired by these exciting results, we then extended the scope of the reaction to other hydrogen donors. In fact, we recognized that the present procedure could be a smooth route for the synthesis of succinic acid derivatives (if decarboxylation is prevented). In fact, a way to incorporate a succinyl unit in a given compound is of valuable interest in view of the well‐known succinylation reaction, an important tool in the functionalization of biomolecules.[^46^, ^47^, ^48^, ^49^, ^50^] Moreover, succinic acid derivatives are key compounds for the preparation of γ‐butyrolactones, butanediols, pyrrolidones, as well as tetrahydrofurans.^[51]^

Thus, we purposely selected other hydrogen donors bearing further functional groups to be introduced during the functionalization of **1 a**, namely: isocapronitrile (**2 o**), cyclopentanone (**2 p**) and 1,3‐benzodioxole (**2 q**). The corresponding succinyl derivatives **3 o**–**3 q** were formed in up to 98 % yield and again flow conditions allowed to increase the yield of isolated products. To ameliorate the lipophilicity of the adducts, cycloalkanes **2 r**–**2 u** and branched alkane **2 v** (20 equiv hydrogen donor required in the latter case) were also used as reaction partners. The functionalization with cyclohexane was consistently more efficient than that with cyclopentane and the effect of the flow apparatus is modest in this case (Scheme 2). To exclude any *E/Z* isomerization of **1 a** under the present photocatalyzed conditions, we monitored by NMR the reaction mixture leading to **3 s** (Figure S9, Supporting Information). Gratifyingly, no formation of maleic acid was detected during the reaction course.

The solubility of cycloheptane and cyclooctane in the reaction mixture (MeCN−H_2_O 9 : 1) is poor, requiring its replacement with a MeCN−CH_2_Cl_2_ 5 : 1 mixture. The last reaction medium, however, is incompatible with the 3D‐printed reactor material (polypropylene), therefore flow conditions were not adopted in the latter case.

**Scheme 3:**
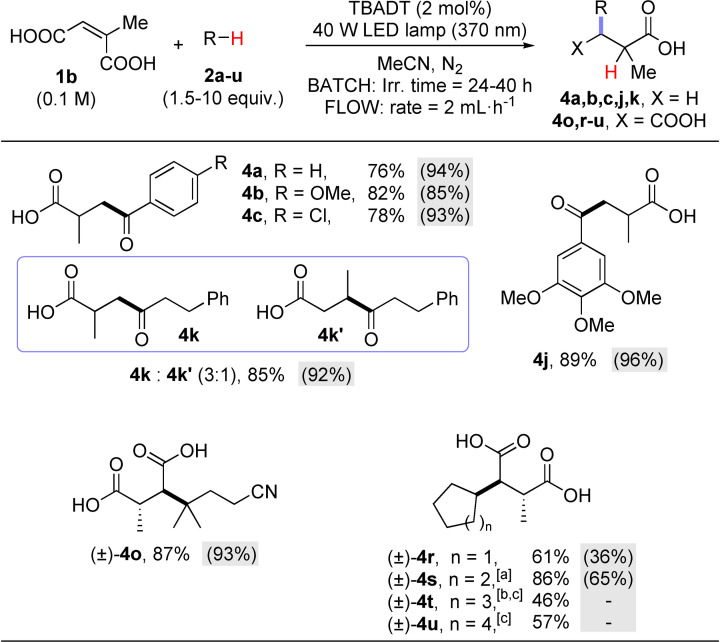
Photocatalyzed functionalization of citraconic acid **1 b**. Isolated yields for reactions performed under batch (2.5 mmol scale) of flow conditions (3D‐printed Reactor A–1.0 mmol scale; figures among parentheses on a gray background). Typical reaction conditions: A solution of citraconic acid **1 b** (0.1 m), hydrogen donors **2 a**–**u** (0.15–1 m) and TBADT (2 mol %) in MeCN was deaerated by argon bubbling for 10 min and irradiated using a 370 nm LED lamp (see Supporting Information for details). [a] Only major diastereoisomer shown; the reaction between **1 b** and **2 s** delivered product **4 s** as a 1.5 : 1 diastereoisomeric mixture arising, respectively, from the *anti* and *syn* addition to the double bond. [b] Only major diastereoisomer shown; the reaction between **1 b** and **2 t** delivered product **4 t** as a 4.5 : 1 diastereoisomeric mixture arising, respectively, from the *anti* and *syn* addition to the double bond. [c] Reaction medium: MeCN/CH_2_Cl_2_ 5 : 1 mixture; reaction not performed under flow conditions due to incompatibility issues with the reactor material.

**Figure 1:**
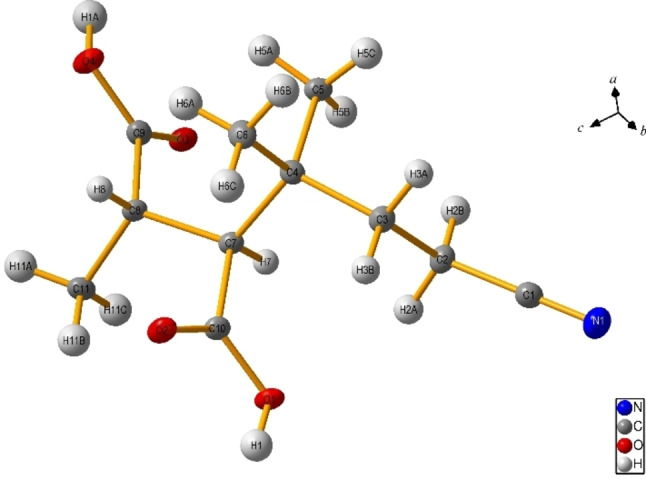
ORTEP view of **4 o** with labeling scheme (thermal ellipsoids are drawn at the 30 % probability level).

In view of the above, we were then intrigued to test the functionalization of citraconic acid (**1 b**, Scheme 3). In fact, the presence of the methyl group may lead to different regio‐/stereo‐ isomers in the desired acylation. As in the case of **1 a**, we initially tested aromatic aldehydes, which led exclusively to acylated/decarboxylated derivatives **4 a**–**4 c**, **4 j**. The reaction between **1 b** and hydrocinnamaldehyde (**2 k**) led, however, to a 3 : 1 mixture of regioisomers (**4 k/4 k′**). The addition of **2 o**, **2 r**–**u** onto **1 b** led again to a single regioisomer in up to quantitative yield. Moreover, a diastereoselective process was observed in the synthesis of **4 o**, **4 r**, **4 u** resulting from the exclusive *anti* addition to the C=C double bond, at variance with the cases of **4 s** and **4 t** where 1.5 : 1 and 4.5 : 1 mixtures of diastereoisomers resulted, respectively (see below and Supporting Information). The stereochemistry of compound **4 o** was confirmed by single‐crystal X‐ray analysis (see Figure 1). In all of these experiments, flow conditions were beneficial to maximize the functionalization yield, except for the case of cycloalkanes (**2 r**, **2 s**) as hydrogen donors (Scheme 3).

Apart from the increased yields, we wanted to verify whether the adoption of flow conditions could be a sensible choice to maximize the productivity of functionalized acids. As detailed in the Supporting Information (section 2), the productivity, expressed as mmol per day of obtained products, is markedly higher in the case of flow conditions, while maintaining the same LED lamp (40 W power), pointing to a better exploitation of the employed light source. In fact, while ca. 1.5 mmol ⋅ day^−1^ can be obtained on average (31 examples) under batch conditions, in the case of flow conditions the same value reaches almost 4.0 mmol day^−1^ on average (27 examples), thus offering a >2.5 times higher productivity (Figure S6). This translates into the possibility to isolate on average ca. 860 mg of product per day (with four products obtained in >1 g day^−1^ amount) when adopting flow conditions, compared to ≈320 mg per day obtained under batch conditions (Figure S7).

After assessing the superior performance offered by flow conditions, to push the productivity potential of the proposed photocatalyzed functionalization we realized an upgraded 3D‐printed reactor (Reactor B; dimensions: 127 mm×180 mm, excluding connectors; exposed surface: ≈229 cm^2^, height: 6 mm; Figure S1), with the same channel dimensions (1 mm) and the same amount of material on top and bottom of the channel (2.5 mm), reaching a total volume of 7.5 mL (Figure S5). As reported in Table 1, we screened a set of conditions for the preparation of **3 a** intended to maximize the overall productivity, also paying attention to the specific productivity parameter, wherein the productivity is normalized with respect to the power of the lamp. Based on the reaction conditions gathered in Scheme 2 (entries 1 and 2 for batch and flow conditions, respectively, in Table 1), we initially verified how the formation of **3 a** was affected by the adopted parameters. Specifically, either the adoption of a less intense light source (20 W) or a higher flow rate (3 mL h^−1^) led to a dramatic decrease of the reaction yield (entries 3, 4). Similarly, the adoption of a higher [**1 a**] (0.15 m) halved the reaction yield (42 %) and the reduction of the flow rate to 1 mL h^−1^ did not ameliorate the reaction performance (entries 5, 6).

**Table 1:** Optimization in terms of productivity of the synthesis of **3 a**.

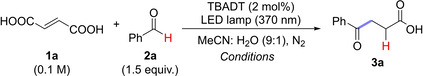									
**Entry**	**Reactor Type**	**1 a concentration [M]**	**Flow rate [mL** **h^−1^]**	**Residence time**	**Lamp power [W]**	**3 a Yield [%]**	**Productivity [mmol** **day^−1^]**	**Specific productivity [mmol** **day^−1^** **W^−1^]**	**Space Time Yield [mmol** **L^−1^** **h^−1^]**
1	Batch (Scheme 2)	0.10	–	24 h	40	81	2.03	0.051	3.4
2	A (Scheme 2)	0.10	2.0	2 h 3′	40	90	4.32	0.108	43.9
3	A	0.10	2.0	2 h 3′	20	35	1.68	0.084	17.1
4	A	0.10	3.0	1 h 22′	40	57	4.10	0.103	41.7
5	A	0.15	2.0	2 h 3′	40	42	3.02	0.076	30.7
6	A	0.15	1.0	4 h 6′	40	43	1.55	0.039	15.7
7	B	0.10	2.0	3 h 45′	40+40	98	4.70	0.059	26.1
8	B	0.15	2.0	3 h 45′	40+40	96	6.91	0.086	38.4
9	B	0.15	4.0	1 h 53′	40+40	99	14.26	0.178	79.2

Having defined the best performance with Reactor A, we then moved to Reactor B. This change prompted us to adopt two LED lamps (for a total power of 80 W) to fully irradiate the reactor exposed surface. To our delight, the reaction conditions previously optimized for Reactor A delivered product **3 a** in >95 % yield (entry 7), albeit the specific productivity calculated in this case was significantly lower due to the adoption of two vs. one LED lamp (compare entries 2 and 7). Nevertheless, when using 0.15 m **1 a** and an increased flow rate (4 mL h^−1^) more than 14 mmol of product per day (>2.5 g) were formed, also reaching the highest specific productivity value, thus confirming the superior performance offered by Reactor B (entries 8, 9). We also calculated an additional performance parameter, namely the space time yield (STY) value, allowing to compare the different reactors employed in the present work. Also in this case, Reactor B offered the best value, thanks to the possibility to adopt both a higher [**1 a**] and flow rate with respect to reactor A, thereby increasing the amount of product obtained while decreasing the residence time within the reactor channels (compare again entries 2 and 9). On the other hand, the productivities and STY offered by batch conditions are significantly lower with respect to the best conditions for either Reactor A or B (compare entry 1 vs. entries 2 and 9).

The procedure described in the present work is a mild route for the smooth synthesis of γ‐keto acids and succinic acid derivatives starting from (possibly biomass‐derived) alkenoic acids. This approach does not require the use of aggressive reagents and well tolerates the acidity of the radical trap, being a problem when adopting basic conditions or when using organometallic species. The generation of radicals is based on the homolytic cleavage of the C(sp^2^)−H bond (in aldehydes **2 a**–**2 n**) or the C(sp^3^)−H bond (in hydrogen donors **2 o**–**2 v**).

**Scheme 4:**
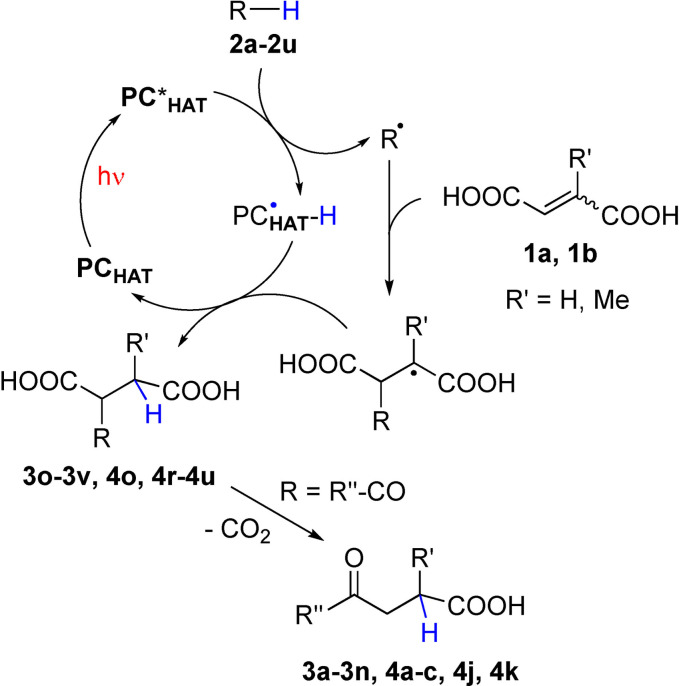
Proposed reaction mechanism.

The mechanism is sketched in Scheme 4 and is based on hydrogen abstraction from **2** by excited TBADT to generate a C‐centered radical that, upon conjugate addition to **1**, leads to the derivatized products **3**,**4**. The PC_HAT_ may be used in a small amount since it is efficiently regenerated by a back hydrogen atom reaction to the radical adduct formed during the process.^[39]^

The fate of the radical addition onto alkenoic acids depends on the nature of both the acid and the hydrogen donor. Acids having an unsubstituted β‐position (acrylic and itaconic acids) should be susceptible of attack by the photogenerated radicals, but this leads to unproductive functionalization^[52]^ probably due to competitive polymerization, as witnessed by some solid formed after the reaction. The situation changes dramatically, however, with 1,4‐butendioic acid derivatives. In fact, aryl,^[53]^ benzyl[^54^, ^55^] and α‐oxy radicals[^56^, ^57^, ^58^] were sparsely used for the functionalization of maleic/fumaric acids, albeit no decarboxylation took place in the reaction course. This behavior is confirmed also in the present work (with donors **2 o**–**2 v**), however, the introduction of an electron‐withdrawing group (such as an acyl moiety) is able to induce a clean decarboxylation to give a γ‐keto acid (Scheme 4).

To the best of our knowledge, in this work we report the first radical functionalization of citraconic acid **1 b**, where two possible regioisomers may be formed. As a matter of fact, in most cases we observed a clean radical addition, since the carbon bearing the methyl group is not functionalized, probably because this is the most hindered site. Furthermore, this regiochemical pathway leads to the formation of the most stable (tertiary) radical adduct. The only exception is the preparation of compound **4 k**, where isomer **4 k′** accounts for ca. 20 % of the mass balance. The modest steric hindrance of the attacking acyl radical (generated from hydrocinnamaldehyde) may account for this result.

**Scheme 5:**
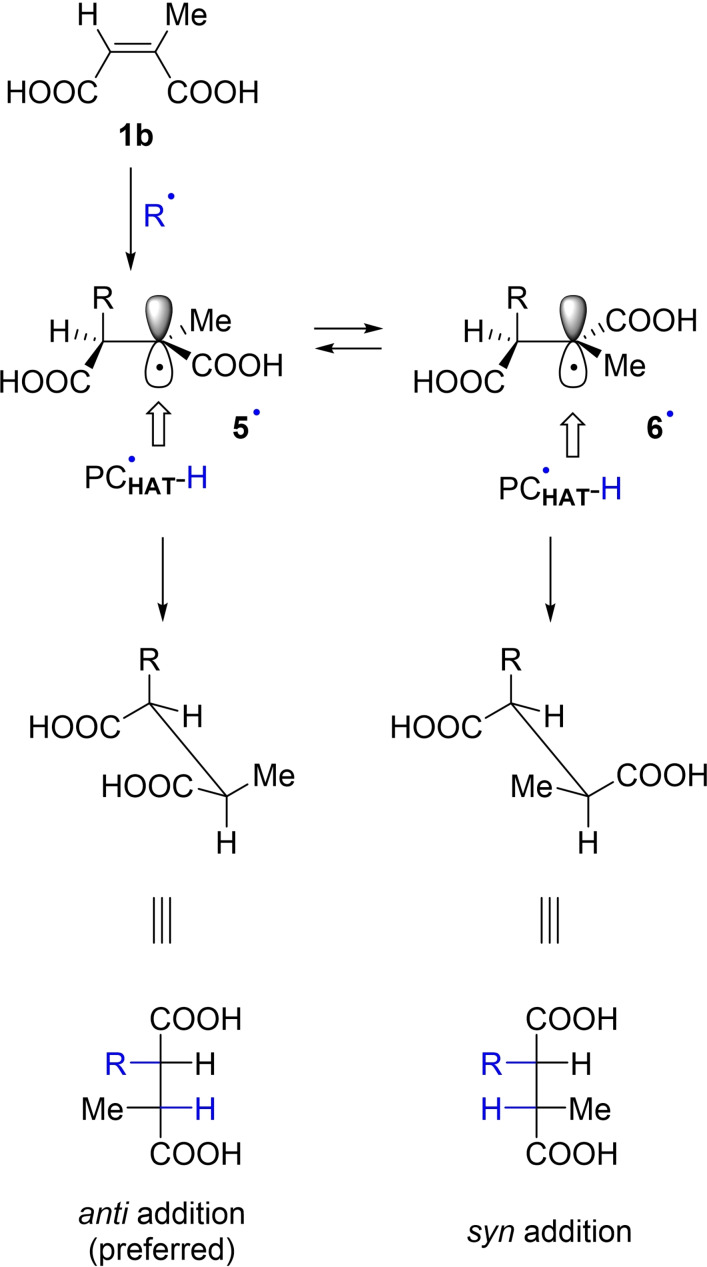
Diastereoselectivity in the radical addition onto **1 b**.

Another interesting issue is the diastereoselective formation of compounds **4 o**, **4 r**–**4 u** where the formation of adducts resulting from an *anti*‐addition of R−H onto the C=C double bond of **1 b** was largely preferred. The attribution of the stereochemistry of the alkylated derivatives was made by comparing the ^13^C NMR signals of the carbon atoms of the carboxylic groups with those of related 2,3‐dialkyl succinic acids^[59]^ and it was supported by the crystal X‐ray analysis of **4 o** (Figure 1). The graphical explanation of the stereochemical assignment is shown in Scheme 5.

First, as demonstrated in the synthesis of **3 s** (see above), no *E/Z* isomerization took place on the starting alkenoic acids during the irradiation. Thus, the regioselective addition of the radical onto **1 b** leads to the conformer **5**⋅. Back hydrogen atom donation from the reduced form of the photocatalyst to **5**⋅ takes place from the opposite side with respect to the entering group R, due to steric repulsion between R and PC⋅_HAT_−H.^[60]^ The R and H moieties (from the R−H substrate) are then placed at opposite sides and an *anti* addition product resulted as the preferred one.

An equilibrium between radical **5**⋅ and **6**⋅ cannot be ruled out,^[61]^ however, enabling the formation of *syn*‐addition products. The latter pathway seems to operate only in the reaction of **1 b** with cyclohexane **2 s** and cycloheptane **2 t**, where both diastereoisomers were formed, although in different amounts. As a matter of fact, this is in line with literature evidence that points to the preferred formation of a single diastereoisomer in the addition of C‐centered radicals on the esters of the *E*‐isomer of **1 b** (dialkyl mesaconates).^[62]^ In fact, the reactivity of an ester‐substituted radical having a tertiary stereogenic carbon atom adjacent to the prochiral radical center is described to show a high 1,2‐stereoinduction deriving from a preferred conformation.^[60]^

## Conclusions

Apart from the importance of the products formed, another crucial issue is the yield/productivity of the functionalized compounds. This is yet another piece of evidence that shifting from batch to flow conditions allowed in most cases to increase the yield of the desired acylation/alkylation products. It is well known, however, that photocatalyzed HAT reactions can be easily carried out under flow conditions.[^38^, ^43^] The challenge here was to obtain significant amounts of products during the scale‐up of the reaction by using the simplest setup possible. 3D‐Printed reactors were the ideal choice because of their low cost and the possibility to implement a flexible tailor‐made design.^[63]^ As a matter of fact, the productivity (mmol day^−1^) was consistently higher under flow conditions with respect to batch conditions (see Table 1). Interestingly, when doubling the surface of the reactor and the light power in passing from Reactor A to B, a higher [**1 a**] and an increased flow rate could be adopted, showing the best performance in terms of STY and specific productivity values. This was again an indication that the correct reactor design was crucial for the scale up of the reaction.

In conclusion, the functionalization of biomass‐derived alkenoic acids could be carried out in a very easy way by flowing the reacting solution in a 3D‐printed reactor upon irradiation with UV LED lamps under photocatalyzed conditions.

## Experimental Section

### Typical procedures for photocatalyzed preparation of compounds 3 and 4

#### Batch conditions

A solution of fumaric acid **1 a** (2.5 mmol, 0.1 m, 290 mg) or citraconic acid **1 b** (2.5 mmol, 0.1 m, 325 mg), hydrogen donors **2 a**–**v** (3.75–50 mmol, 0.15–2 m; 1.5–20 equiv) and TBADT (2 mol %, 166 mg) in 25 mL of a MeCN : H_2_O (9 : 1) mixture or MeCN for **1 a** and **1 b**, respectively, was poured in a Pyrex vessel, deaerated (by argon bubbling for 10 min) and irradiated for 24–40 h using a 370 nm LED lamp (Kessil PR‐160 L, 40 W; see experimental setup in Figure S3) by applying fan cooling to keep temperature below 30 °C. The progress of the reaction was monitored by GC‐FID and, upon completion, the crude mixture was poured into a round‐bottom flask and the solvent removed via rotary evaporation. Then, the reaction product was isolated by column chromatography using SiO_2_ as stationary phase and mixtures of cyclohexane/ethyl acetate as eluants.

#### Flow conditions

A solution of fumaric acid **1 a** (1 mmol, 0.1 M, 116 mg) or citraconic acid **1 b** (1 mmol, 0.1 M, 130 mg), hydrogen donor **2 a**–**s** (1.5–10 mmol, 0.15–1 M; 1.5–10 equiv.), and TBADT (2 mol %, 66 mg) in 10 mL of a MeCN : H_2_O (9 : 1) mixture or MeCN for **1 a** and **1 b**, respectively, was deaerated (by argon bubbling for 10 min) and charged into a coiled tubing reservoir (PTFE, internal diameter: 1 mm; see Figure S4). The reaction mixture was then flown through the channels of the chosen 3D‐printed reactor (reactor A or B) by means of a syringe pump (Figure S4) using a flow rate of 2 mL h^−1^ upon irradiation with a 370 nm LED lamp (Kessil PR‐160 L, 40 W; see experimental setup in Figure S5) by applying fan cooling to keep temperature below 30 °C. The progress of the reaction was monitored by GC‐FID and, upon completion, the crude mixture was poured into a round‐bottom flask and the solvent removed via rotary evaporation. Then, the reaction product was isolated by column chromatography using SiO_2_ as stationary phase and mixtures of cyclohexane/ethyl acetate as eluants.

### Accession Codes

The deposition number CCDC 2177823 (**4 o**) contains the supplementary crystallographic data for this paper. These data can be obtained free of charge via http://www.ccdc.cam.ac.uk/data request/cif or Cambridge Crystallographic Data Centre, 12 Union Road, Cambridge CB2 1EZ, UK; fax: +44 1223 336033.

## Conflict of interest

The authors declare no conflict of interest.

1

## Supporting information

As a service to our authors and readers, this journal provides supporting information supplied by the authors. Such materials are peer reviewed and may be re‐organized for online delivery, but are not copy‐edited or typeset. Technical support issues arising from supporting information (other than missing files) should be addressed to the authors.

Supporting Information

## Data Availability

The data that support the findings of this study are available in the supplementary material of this article.

## References

[1] L. R. Lynd , M. S. Laser , D. Bransby , B. E. Dale , B. Davison , R. Hamilton , M. Himmel , M. Keller , J. D. McMillan , J. Sheehan , C. E. Wyman , Nat. Biotechnol. 2008, 26, 169–172. 18259168 10.1038/nbt0208-169

[2] T. Werpy , G. Petersen , Top value added chemicals from biomass: volume I – results of screening for potential candidates from sugars and synthesis gas, Washington DC: US Department of Energy (US) 2004.

[3] P. M. Foley , E. S. Beach , J. B. Zimmerman , Green Chem. 2011, 13, 1399–1405.

[4] P. T. Anastas , J. B. Zimmerman , Environ. Sci. Technol. 2003, 37, 94A–101A. 10.1021/es032373g 12666905

[5] F. H. Isikgor , C. R. Becer , Polym. Chem. 2015, 6, 4497–4559.

[6] S. Y. Lee , H. U. Kim , T. U. Chae , J. S. Cho , J. W. Kim , J. H. Shin , D. I. Kim , Y.-S. Ko , W. D. Jang , Y.-S. Jang , Nat. Catal. 2019, 2, 18–33.

[7] A–Z of Biorefinery, A Comprehensive View (Eds.: N. Thongchul , A. Kokossis , S. Assabumrungrat ), Elsevier Inc, 2021, pp. 389–506.

[8] J. Iglesias , I. Martínez-Salazar , P. Maireles-Torres , D. Martin Alonso , R. Mariscal , M. López Granados , Chem. Soc. Rev. 2020, 49, 5704–5771. 10.1039/d0cs00177e 32658221

[9] S. Yang , M. Kim , S. Yang , D. Sung Kim , W. Jae Lee , H. Lee , Catal. Sci. Technol. 2016, 6, 3616–3622.

[10] G. Yin , H. Zhong , G. Yao , F. Jin , J. Zhao , Energies 2021, 14, 5456.

[11] J. G. H. Hermens , A. Jensma , B. L. Feringa , Angew. Chem. Int. Ed. 2022, 61, e202112618; 10.1002/anie.202112618 PMC9299676 34783426

[12] G. B. Pedroso , S. Montipó , D. A. N. Mario , S. H. Alves , A. F. Martins , Biomass Convers. Bior. 2017, 7, 23–35.

[13] R. Bafana , R. A. Pandey , Crit. Rev. Biotechnol. 2018, 38, 68–82. 28425297 10.1080/07388551.2017.1312268

[14] B. C. Saha , J. Ind. Microbiol. Biotechnol. 2017, 44, 303–315. 27933436 10.1007/s10295-016-1878-8

[15] L. Regestein , T. Klement , P. Grande , D. Kreyenschulte , B. Heyman , T. Maßmann , A. Eggert , R. Sengpiel , Y. Wang , N. Wierckx , L. M. Blank , A. Spiess , W. Leitner , C. Bolm , M. Wessling , A. Jupke , M. Rosenbaum , J. Büchs , Biotechnol. Biofuels 2018, 11, 279. 30337958 10.1186/s13068-018-1273-y PMC6180396

[16] B.-E. Teleky , D. C. Vodnar , Polymer 2019, 11, 1035.

[17] J. Yang , H. Xu , J. Jiang , N. Zhang , J. Xie , M. Wei , J. Zhao , J. Bioresour. Bioprod. 2019, 4, 135–142.

[18] F. M. Haque , et al., Chem. Rev. 2022, 122, 6322–6373. 35133803 10.1021/acs.chemrev.1c00173

[19] J. R. Elmore , G. N. Dexter , D. Salvachúa , J. Martinez-Baird , E. A. Hatmaker , J. D. Huenemann , D. M. Klingeman , G. L. Peabody V , D. J. Peterson , C. Singer , G. T. Beckham , A. M. Guss , Nat. Commun. 2021, 12, 2261. 33859194 10.1038/s41467-021-22556-8 PMC8050072

[20] Y. Rodenas , R. Mariscal , J. L. G. Fierro , D. Martín Alonso , J. A. Dumesic , M. López Granados , Green Chem. 2018, 20, 2845–2856.

[21] T. Yang , W. Li , Q. Liu , M. Su , T. Zhang , J. Ma , BioResources 2019, 14, 5025–5044.

[22] S.-Y. Jeong , J.-W. Lee , Energies 2021, 14, 918.

[23] A. J. J. Straathof , W. M. van Gulik , Reprogramming Microbial Metabolic Pathways. Subcellular Biochemistry (Eds.: X. Wang , J. Chen , P. Quinn ), Springer, Dordrecht 2012, pp 225–240.

[24] C. A. Roa Engel , A. J. J. Straathof , T. W. Zijlmans , W. M. van Gulik , L. A. M. van der Wielen , Appl. Microbiol. Biotechnol. 2008, 78, 379–389. 18214471 10.1007/s00253-007-1341-x PMC2243254

[25] G. Xu , W. Zou , X. Chen , N. Xu , L. Liu , J. Chen , PLoS One 2012, 7, e52086. 23300594 10.1371/journal.pone.0052086 PMC3530589

[26] V. Martin-Dominguez , J. Estevez , F. de Borja Ojembarrena , V. E. Santos , M. Ladero , Fermentation 2018, 4, 33.

[27] A. Jiménez-Quero , E. Pollet , M. Zhao , E. Marchioni , L. Avérous , V. Phalip , J. Microbiol. Biotechnol. 2016, 26, 1557–1565. 27291673 10.4014/jmb.1603.03073

[28] A. Bohre , U. Novak , M. Grilc , B. Likozar , J. Mol. Catal. 2019, 476, 110520.

[29] K. Avasthi , A. Bohre , M. Grilc , B. Likozar , B. Saha , Catal. Sci. Technol. 2020, 10, 5411–5437.

[30] J. Lebeau , J. P. Efromson , M. D. Lynch , Front. Bioeng. Biotechnol. 2020, 8, 207. 32266236 10.3389/fbioe.2020.00207 PMC7100375

[31] A. Bohre , K. Avasthi , U. Novak , B. Likozar , ACS Sustainable Chem. Eng. 2021, 9, 2902–2911. 10.1021/acssuschemeng.0c08976 PMC8025712 33842102

[32] Y. Wu , M. Shetty , K. Zhang , P. J. Dauenhauer , ACS Eng. Au 2022, 2, 92–102.

[33] B. Chen , L. Chen , Z. Yan , J. Kang , S. Chen , Y. Jin , L. Ma , H. Yan , C. Xia , Green Chem. 2021, 23, 3607–3611.

[34] A. Albini , M. Fagnoni , ChemSusChem 2008, 1, 63–66. 18605663 10.1002/cssc.200700015

[35] N. Hoffmann , Photochem. Photobiol. Sci. 2012, 11, 1613–1641. 22732723 10.1039/c2pp25074h

[36] H. E. Bonfield , T. Knauber , F. Lévesque , E. G. Moschetta , F. Susanne , L. J. Edwards , Nat. Commun. 2020, 11, 804. 32029723 10.1038/s41467-019-13988-4 PMC7004975

[37] A. Parodi , A. Jorea , M. Fagnoni , D. Ravelli , C. Samorì , C. Torri , P. Galletti , Green Chem. 2021, 23, 3420–3427.

[38] L. Capaldo , D. Ravelli , M. Fagnoni , Chem. Rev. 2022, 121, 1875–1924. 10.1021/acs.chemrev.1c00263 PMC8796199 34355884

[39] D. Ravelli , S. Protti , M. Fagnoni , Acc. Chem. Res. 2016, 49, 2232–2242. 27648722 10.1021/acs.accounts.6b00339

[40] A. A. Siddiqui , S. Partap , S. Khisal , M. Shahar Yar , R. Mishra , Bioorg. Chem. 2020, 99, 103584. 32229345 10.1016/j.bioorg.2020.103584

[41] A. A. Abu-Hashem , J. Heterocycl. Chem. 2021, 58, 74–92.

[42] L. Capaldo , R. Riccardi , D. Ravelli , M. Fagnoni , ACS Catal. 2018, 8, 304–309.

[43] L. Buglioni , F. Raymenants , A. Slattery , S. D. A. Zondag , T. Noël , Chem. Rev. 2022, 122, 2752–2906, and reference therein. 34375082 10.1021/acs.chemrev.1c00332 PMC8796205

[44] S. Protti , D. Ravelli , M. Fagnoni , Photochemical processes in continuous-flow reactors: From engineering principles to chemical applications (Ed.: T. Noël ), World Scientific Publishing Europe Ltd, London 2017, pp. 1–36.

[45] A. J. N. Price , A. J. Capel , R. J. Lee , P. Pradel , S. D. R. Christie , J. Flow Chem. 2021, 11, 37–51.

[46] L. Mirmoghtadaie , M. Kadivar , M. Shahedi , Food Chem. 2009, 114, 127–131.

[47] R. Caillard , A. Petit , M. Subirade , Int. J. Biol. Macromol. 2009, 45, 414–420. 19576929 10.1016/j.ijbiomac.2009.06.013

[48] B. G. Shilpashree , S. Arora , P. Chawla , Food Res. Int. 2015, 72, 223–230.

[49] Z. Söyler , K. N. Onwukamike , S. Grelier , E. Grau , H. Cramail , M. A. R. Meier , Green Chem. 2018, 20, 214–224.

[50] C. Espro , E. Paone , F. Mauriello , R. Gotti , E. Uliassi , M. L. Bolognesi , D. Rodríguez-Padrón , R. Luque , Chem. Soc. Rev. 2021, 50, 11191–11207. 34553208 10.1039/d1cs00524c

[51] A. Cukalovic , C. V. Stevens , Biofuels Bioprod. Biorefin. 2008, 2, 505–529.

[52] The acylation of acrylic acid likewise failed when generating the benzoyl radical from 2-oxophenylacetic acid under photoredox catalyzed conditions: See G.-Z. Wang , R. Shang , W.-M. Cheng , Y. Fu , Org. Lett. 2015, 17, 4830–4833.26366608

[53] A. Citterio , A. Cominelli , F. Bonavoglia , Synthesis 1986, 4, 308–309.

[54] L. Cermenati , M. Mella , A. Albini , Tetrahedron 1998, 54, 2575–2582.

[55] L. Cermenati , M. Fagnoni , A. Albini , Can. J. Chem. 2003, 81, 560–566.

[56] D. Dondi , S. Protti , A. Albini , S. Mañas Carpio , M. Fagnoni , Green Chem. 2009, 11, 1653–1659.

[57] M. Hayakawa , H. Shirota , S. Hirayama , R. Yamada , T. Aoyama , A. Ouchi , J. Photochem. Photobiol. A 2021, 413, 113263.

[58] M. Hayakawa , R. Shimizu , H. Omori , H. Shirota , K. Uchida , H. Mashimo , H. Xu , R. Yamada , S. Niino , Y. Wakame , C. Liu , T. Aoyama , A. Ouchi , Tetrahedron 2020, 76, 131557.

[59] L. Ernst , W. Trowitzsch , Chem. Ber. 1974, 107, 3771–3779.

[60] P. Erdmann , J. Schäfer , R. Springer , H.-G. Zeitz , B. Giese , Helv. Chim. Acta 1992, 75, 638–644.

[61] Y. Cai , B. P. Roberts , D. A. Tocher , S. A. Barnett , Org. Biomol. Chem. 2004, 2, 2517–2529. 15326533 10.1039/B407215B

[62] J. C. Tripp , C. H. Schiesser , D. P. Curran , J. Am. Chem. Soc. 2005, 127, 5518–5527. 15826190 10.1021/ja042595u

[63] M. C. Maier , A. Valotta , K. Hiebler , S. Soritz , K. Gavric , B. Grabner , H. Gruber-Woelfler , Org. Process Res. Dev. 2020, 24, 2197–2207.

