# Supplementary material for: Photocatalyzed Functionalization of Alkenoic Acids in 3D‐Printed Reactors
Source: ChemSusChem. 2022 Jul 8;15(17):e202200898. doi: 10.1002/cssc.202200898 (PMC9543820; doi:10.1002/cssc.202200898)

ChemSusChem

Supporting Information

Photocatalyzed Functionalization of Alkenoic Acids in 3D-Printed Reactors

Alexandra Jorea, Davide Ravelli, Rodrigo M. Romarowski, Stefania Marconi, Ferdinando Auricchio, and Maurizio Fagnoni*© 2022 The Authors. ChemSusChem published by Wiley-VCH GmbH. This is an open access article under the terms of the Creative Commons Attribution License, which permits use, distribution and reproduction in any medium, provided the original work is properly cited.

Supporting Information

Photocatalyzed functionalization of alkenoic acids in 3D-printed reactors

Alexandra Jorea,[a,b] Davide Ravelli,[b] Rodrigo M. Romarowski,[c] Stefania Marconi,[c] Ferdinando Auricchio,[c] Maurizio Fagnoni*[b]

[a] Mrs Alexandra Jorea
Department of Clinical Surgery, Diagnostics and Pediatrics
Fondazione IRCCS Policlinico San Matteo
Viale Brambilla 74, 27100 Pavia, Italy

[b] Mrs Alexandra Jorea, Prof. Davide Ravelli, Prof. Maurizio Fagnoni
PhotoGreen Lab, Department of Chemistry,
University of Pavia, viale Taramelli 12, 27100 Pavia, Italy
E-mail: fagnoni@unipv.it

[c] Dr. Rodrigo M. Romarowski, Dr. Stefania Marconi, Prof. Ferdinando Auricchio,
Computational Mechanics and Advanced Materials Group,
University of Pavia, via Ferrata 3, Pavia, Italy

CONTENT (57 pages)

1. Experimental section **S2**

1.1 General information **S2**

1.2 Typical procedures for photocatalyzed functionalizations **S6**

2. Reactors performance evaluation **S9**

3. Characterization of products **S12**

4. References **S25**

5. Copy of ^{1}H and ^{13}C NMR spectra **S26**

1. Experimental section

1.1 General information

Alkenoic acids **1** and H-donors **2** were commercially available and used as received. Acetonitrile and water (HPLC grade) employed for the photochemical reactions was used as received. TBADT was synthesized as described elsewhere.[S1]

NMR spectra were recorded on a 300 (for ^{1}H) or 75 (for ^{13}C) MHz spectrometer; the attributions were made based on ^{1}H and ^{13}C NMR. Data for ^{1}H NMR are reported as follows: chemical shift referred to TMS (δ ppm), multiplicity (s = singlet, bs = broad singlet, d = doublet, t = triplet, q = quadruplet, quint = quintuplet, sext = sextuplet, sept = septuplet, m = multiplet), coupling constant (Hz) and integration. Data for ^{13}C NMR are reported in terms of chemical shift.

GC-FID analyses were performed on an Agilent 7820A. The injection was performed at 250 °C in split mode. The initial oven temperature of 80 °C was maintained for 2 min, increased by 10 °C/min to 250 °C and held for 5 min. An Agilent HP5 30 m × 0.32 mm × 0.25 μm film thickness capillary column was used with nitrogen as the carrier gas at a constant flow rate of 6.0 mL·min^{-1}.

GC/MS analyses were carried out on a Thermo Scientific DSQII single quadrupole GC/MS system. The injection in the GC/MS system was performed at 250 °C in split mode. The initial oven temperature of 80 °C was maintained for 5 min, increased by 10 °C/min to 250 °C and held for 10 min. A Restek Rxi-5ms 30 m × 0.25 mm × 0.25 μm film thickness capillary column was used with helium as the carrier gas at a constant flow rate of 1.0 mL/min. The transfer line temperature was 270 °C and the ion source temperature was 250 °C. Electron ionization mode was used with 70 eV and the ions were registered in full scan mode in a mass range of m/z 40-600 amu. The chromatogram acquisition, detection of mass spectral peaks and their waveform processing were performed using Xcalibur MS Software Version 2.1 (Thermo Scientific Inc.). Assignment of chemical structures to chromatographic peaks was based on the comparison with the databases for GC-MS NIST Mass Spectral Library (NIST 08) and Wiley Registry of Mass Spectral Data (8th Edition).

HRMS data were acquired using a X500B QTOF System (SCIEX, Framingham, MA 01701 USA) available at the CGS of the University of Pavia, equipped with the Twin Sprayer ESI probe and coupled to an ExionLC™ system (SCIEX). The SCIEX OS software 2.1.6 was used as operating platform. For MS detection the following parameters were applied: curtain gas: 30 psi, ion source gas 1: 45 psi, ion source gas 2: 55 psi, temperature: 450 °C, polarity negative, ion spray voltage: -4500 V, TOF mass range: 50-1600 Da, declustering potential: -60 V and collision energy: -10 V.

Single-crystal X-ray diffraction measurements were carried out at CRIST (Centro di Cristallografia of the University of Florence) on a prismatic crystal by using a Bruker D8 Venture equipped with a Photon III pixel array detector, with graphite-monochromatized CuKα radiation (λ = 1.5418 Å). The intensity data were integrated and corrected for standard Lorentz polarization factors with the software package APEX4 v2021.10-0. A total of 2162 unique reflections were collected up to θ= 68.38°.

The 3D-printed reactors (see schematic drawings in Figure S1) have been designed using a CAD software (Autodesk Inventor 2021), relying on the design previously reported.[S2] The final model has then been exported into a Standard Triangulation Language (.STL) file format, which underwent to the so-called slicing procedure, in order to generate the G-code, that is the set of instructions required to run the production by means of 3D printing.

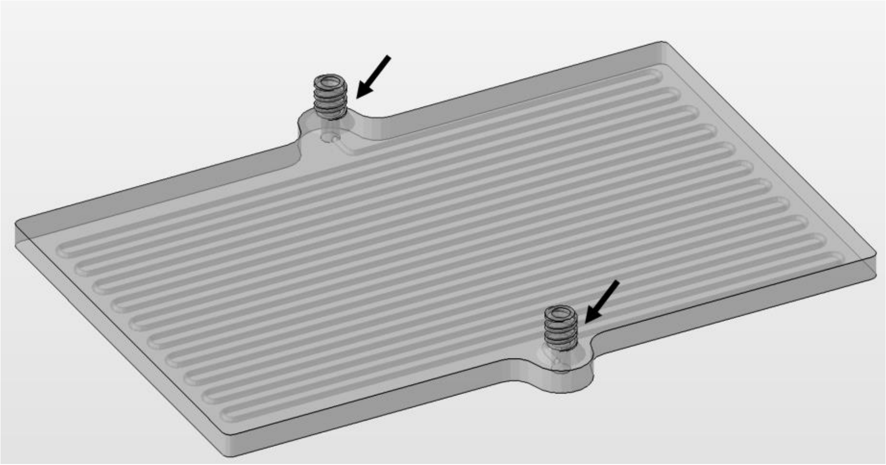

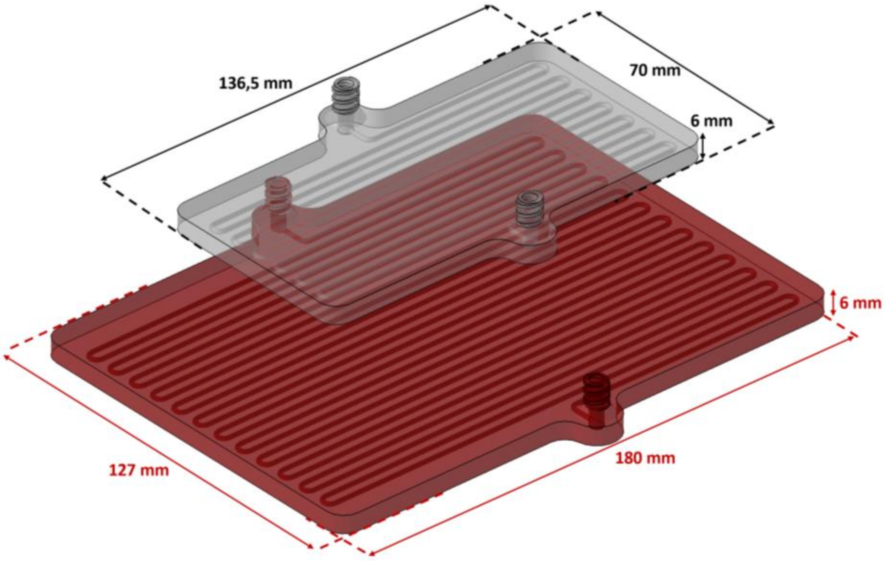

Figure S1. Schematic drawing of 3D-printed reactors, showing the channel path and the inflow / outflow connectors (black arrows; upper part); Schematic drawing of Reactor A (gray) and Reactor B (red) with their respective overall dimensions (bottom part).

The reactor has been produced by means of a 3NTR A4v2 (3ntr © Jdeal-Form srl), a Fused Filament Fabrication 3D printing machine employing a 1.75mm Polypropylene filament (Ultimaker PP) as printing material and a 2.85mm High Impact PolyStyrene filament (3NTR) as support material. The support material has been used only to create the raft, that is a polymeric base on which the model is built and that is removed right after the print is finished. Depending on the specific features of the 3D printer employed for the production, it might be avoided.

The G-code has been generated using the slicing software Ultimaker Cura, employing the following main parameters:

Polypropylene printing temperature: 225 °C
HIPS printing temperature: 255 °C
Build plate temperature: 120 °C

Layer Height: 0.20 mm
Line Width: 0.40 mm
Wall Line Count: 4
Wall Speed: 25mm/s
Fill Gaps: Everywhere
Top/Bottom Pattern: Lines
Top/Bottom Line Directions: -45° / +45°
Top/Bottom Speed: 50mm/s
Infill Density: 100%
Infill Pattern: Lines
Infill Line Directions: 45° / +45°
Infill Speed: 50mm/s
Build Plate Adhesion: Raft and Brim
Brim Width: 12mm

All the speeds have been reduced and fan speed increased at the level of the luer lock connectors, being the area to be printed in each layer smaller than the one of the reactors.

We also verified the optical properties of the polypropylene (PP) material employed to print the reactors, which were compared to those of the Pyrex vessel adopted for batch reactions. Figure S2 shows the absorbance behavior in the UV-Vis range of a 2.5 mm thick 3D-printed PP layer (corresponding to the distance between the reactor surface and the channels) and that offered by the Pyrex vessel.

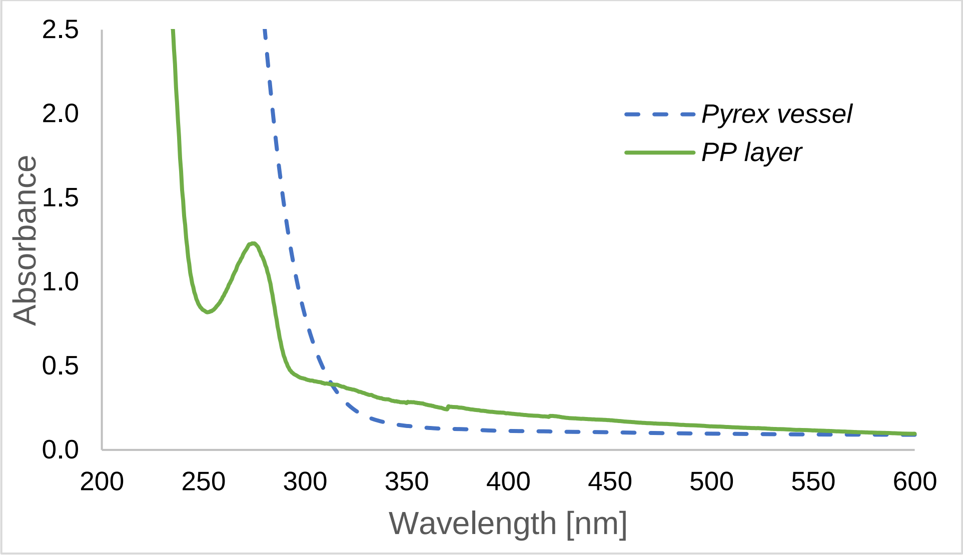

Figure S2. Absorbance plots in the UV-Vis range of: a 2.5 mm thick PP layer (solid green line) and the Pyrex vessel adopted for batch processes (dashed blue line).

1.2 Typical procedures for photocatalyzed functionalizations

Batch conditions A solution of fumaric acid **1a** (2.5 mmol, 0.1 M, 290 mg) or citraconic acid **1b** (2.5 mmol, 0.1 M, 325 mg), hydrogen donors **2a-v** (3.75-50 mmol, 0.15-2 M; 1.5-20 equiv.) and TBADT (2 mol%, 166 mg) in 25 mL of a MeCN:H_2O (9:1) mixture or MeCN for **1a** and **1b**, respectively (except where otherwise noted), was poured in a Pyrex vessel, deaerated (by argon bubbling for 10 minutes) and irradiated for 24-40 h using a 370 nm LED lamp (Kessil PR-160L, 40 W; see experimental setup in Figure S3) by applying fan cooling to keep temperature below 30 °C. The progress of the reaction was monitored by GC-FID and, upon completion, the crude mixture was poured into a round-bottom flask and the solvent removed via rotary evaporation. Then, the reaction product was isolated by column chromatography using SiO_2 as stationary phase and mixtures of cyclohexane/ethyl acetate as eluants.

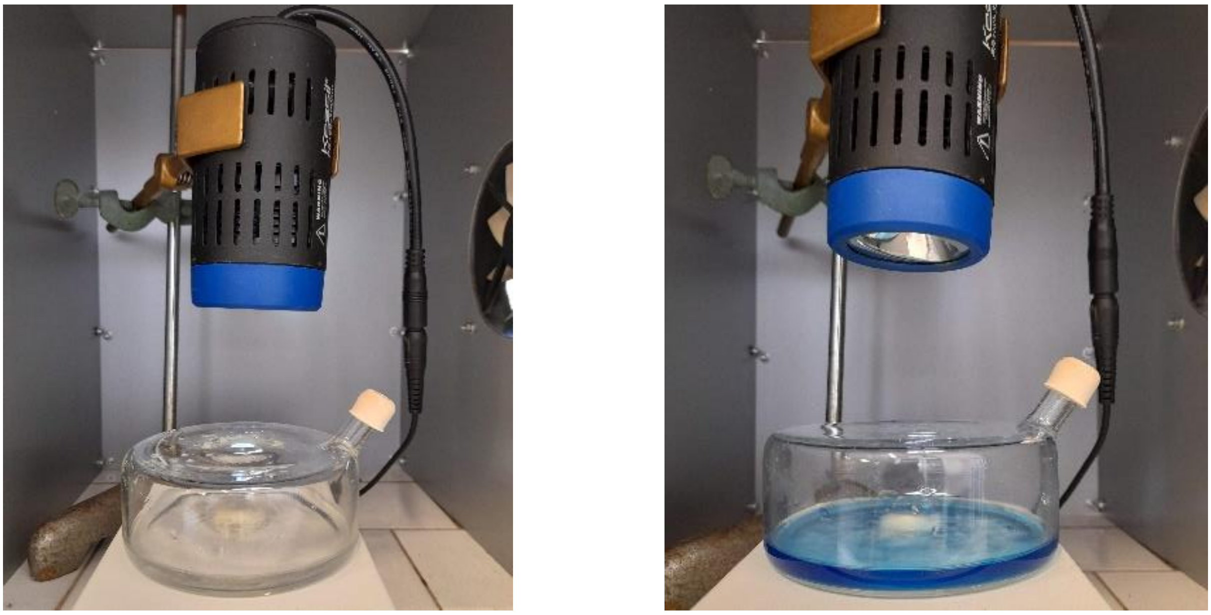

Figure S3. Experimental setup used for photocatalyzed functionalizations under batch conditions before (left part) and after (right part) irradiation.

Flow conditions A solution of fumaric acid **1a** (1 mmol, 0.1M, 116 mg) or citraconic acid **1b** (1 mmol, 0.1M, 130 mg), hydrogen donor **2a-s** (1.5-10 mmol, 0.15-1 M; 1.5-10 equiv.), and TBADT (2 mol%, 66 mg) in 10 mL of a MeCN:H_2O (9:1) mixture or MeCN for **1a** and **1b**, respectively, was deaerated (by argon bubbling for 10 minutes) and charged into a coiled tubing reservoir (PTFE, internal diameter: 1 mm; Figure S4, left part). The reaction mixture was then flown through the channels of the chosen 3D-printed reactor (reactor A or B) by means of a syringe pump (Figure S4, right part) using a flow rate of 2 mL·h^{-1} (except where otherwise noted) upon irradiation with a 370 nm LED lamp (Kessil PR-160L, 40 W; see experimental setup in Figure S5) by applying fan cooling to keep temperature below 30 °C. The progress of the reaction was monitored by GC-FID and, upon completion, the crude mixture was poured into a round-bottom flask and the solvent removed via rotary evaporation. Then, the reaction product was isolated by column chromatography using SiO_2 as stationary phase and mixtures of cyclohexane/ethyl acetate as eluants.

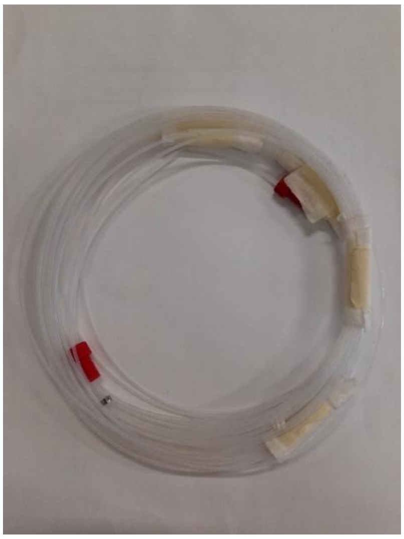

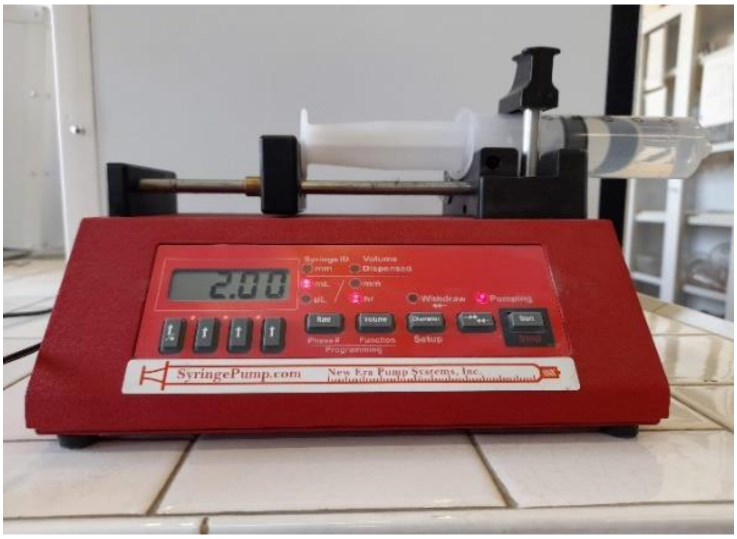

Figure S4. Experimental setup used for photocatalyzed functionalizations under flow conditions: coiled tubing reservoir (left part) and syringe pump (right part).

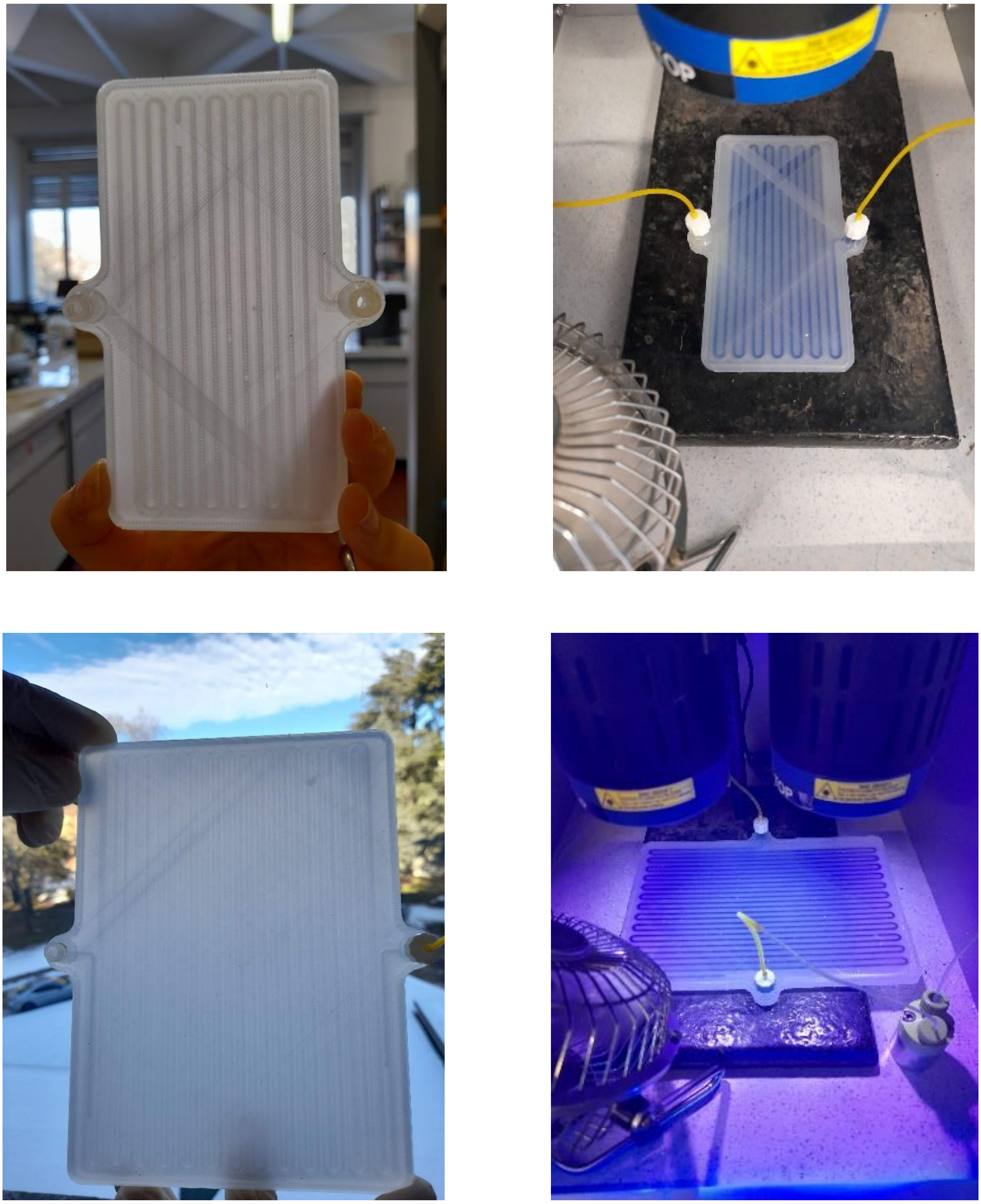

Figure S5. Experimental setup used for photocatalyzed functionalizations under flow conditions: 3D-printed reactor A (top part) and 3D-printed reactor B (bottom part).

2. Reactors performance evaluation

The performance offered by batch and flow conditions in terms of productivity of the desired compounds has been assessed by calculating the amounts of product that can be obtained in one day.

The following equations have been adopted for batch conditions:

$$\textbf{BATCH Productivity [mmol·day}^{-1}\textbf{]} = \frac{2.5\ \text{mmol} \cdot \text{Reaction Yield [\%]} \cdot \text{Irradiation Time [h]}}{24\ ^{\text{hours}}/_{\text{day}}}$$

$$\textbf{BATCH Productivity [g·day}^{-1}\textbf{]} =$$

$$\frac{0.025\ \text{mol} \cdot \text{MW}_{\text{Product}}\ [\text{g·mol}^{-1}] \cdot \text{Reaction Yield [\%]} \cdot \text{Irradiation Time [h]}}{24\ ^{\text{hours}}/_{\text{day}}}$$

The following equations have been adopted for flow conditions:

$$\textbf{FLOW Productivity [mmol·day}^{-1}\textbf{]} =$$

$$\textbf{1}\ \text{Concentration [mmol·mL}^{-1}] \cdot \text{Flow Rate [mL·h}^{-1}] \cdot \text{Reaction Yield [\%]} \cdot 24\ ^{\text{hours}}/_{\text{day}}$$

$$\textbf{FLOW Productivity [g·day}^{-1}\textbf{]} =$$

$$\textbf{1}\ \text{Concentration [mmol·mL}^{-1}] \cdot \text{Flow Rate [mL·h}^{-1}] \cdot \text{MW}_{\text{Product}}\ [\text{g·mol}^{-1}] \cdot \text{Reaction Yield [\%]} \cdot 24\ ^{\text{hours}}/_{\text{day}}$$

Space-time Yield (STY) values for the different experimental setups have been calculated according to the following equation:

$$\textbf{STY [mmol·L}^{-1}\textbf{·h}^{-1}\textbf{]} = \frac{\text{Amount of } \textbf{3} \text{ obtained [mmol]}}{\text{Reactor volume [L]} \cdot \text{Irradiation Time [h]}}$$

Figures S6 and S7 depict the data obtained upon application of the above-mentioned equations to the reactions gathered in Schemes 2 and 3 in the main text.

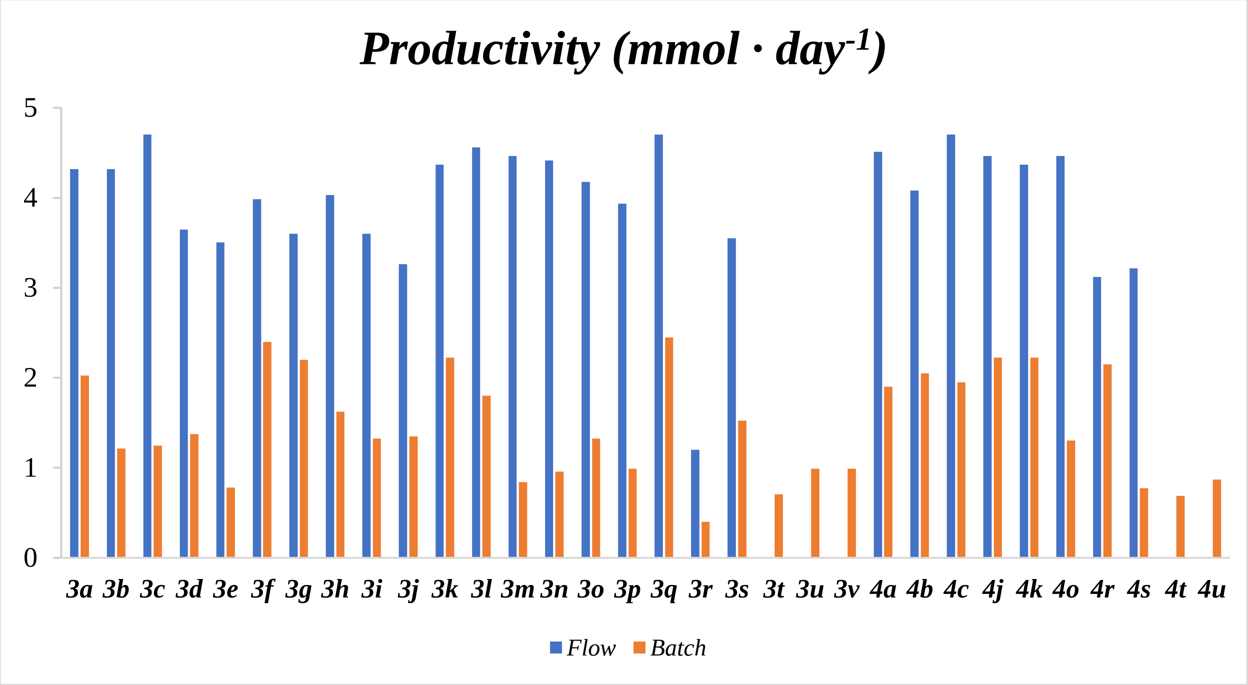

Figure S6. Productivity [mmol·day^{-1}] comparison between batch and flow conditions.

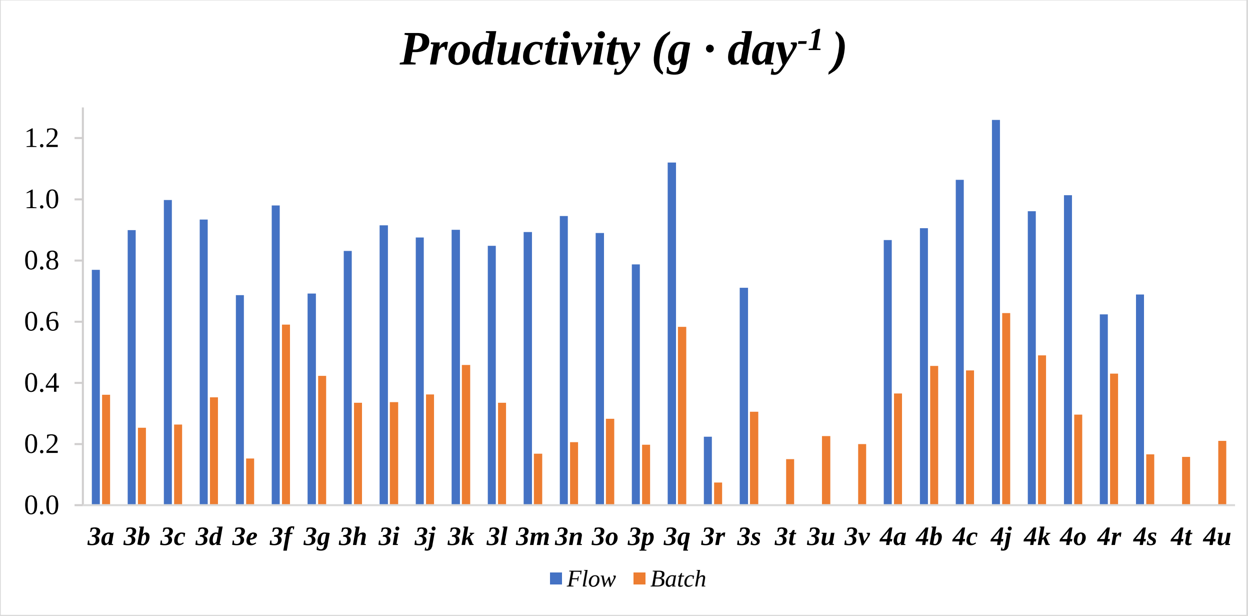

Figure S7. Productivity [g·day^{-1}] comparison between batch and flow conditions.

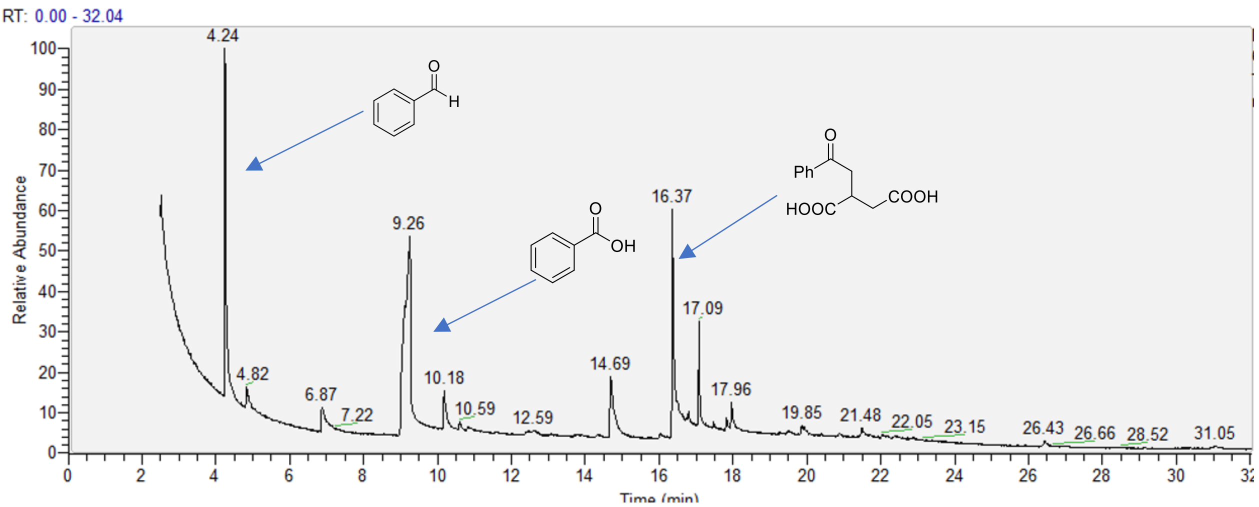

Figure S8. GC-MS analysis of a mixture of itaconic acid **II** and benzaldehyde irradiated at 370 nm for 24h in the presence of TBADT (2 mol%). The desired acylated product was detected in a low amount, being benzoic acid the main product.

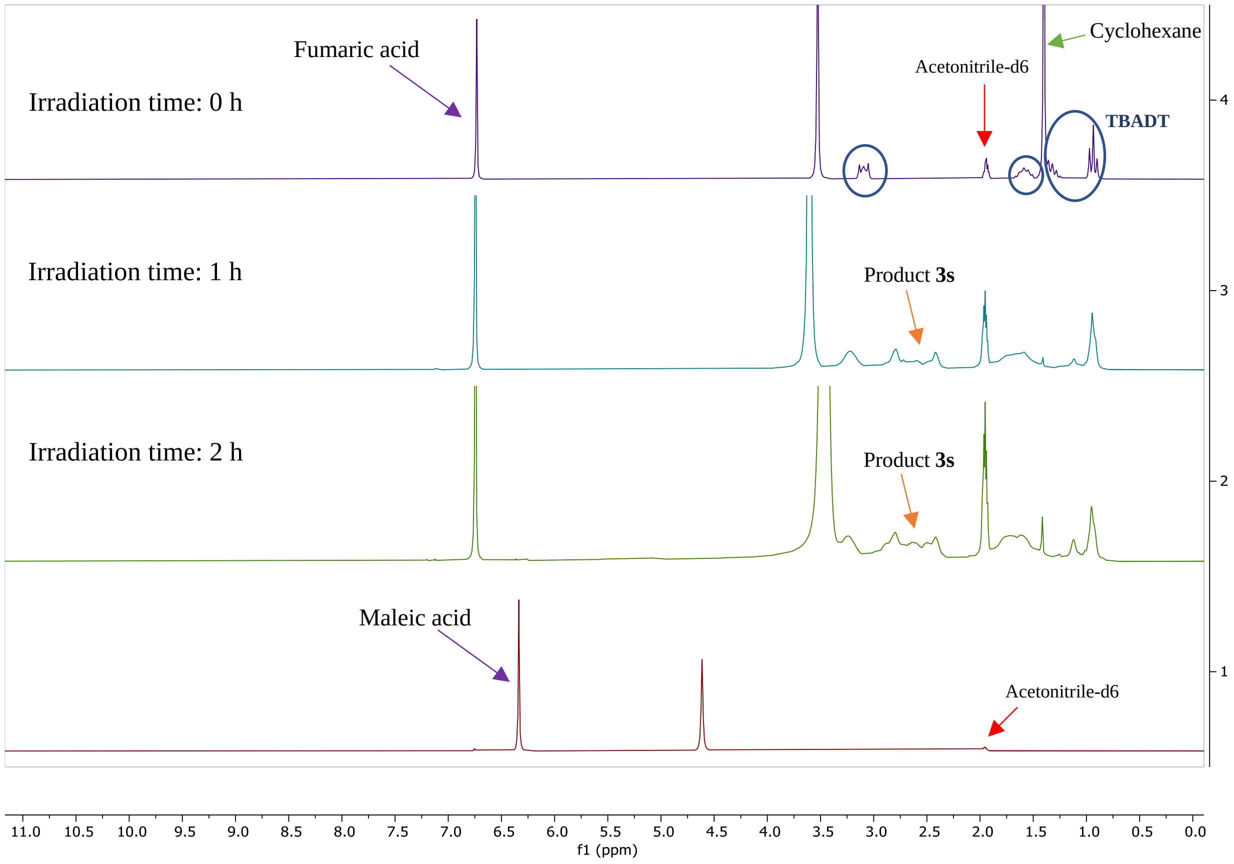

Figure S9. NMR monitoring of the preparation of **3s** starting from fumaric acid **1a**. No *E/Z* isomerization to maleic acid has been observed upon irradiation of the reaction mixture.

3. Characterization of products

4-oxo-4-phenylbutanoic acid (3a)

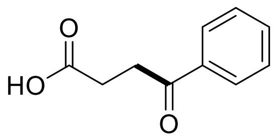

Prepared from fumaric acid **1a** (batch: 2.5 mmol, 290 mg; flow: 1 mmol, 116 mg) and benzaldehyde **2a** (1.5 equiv.; batch: 3.75 mmol, 380 μL; flow: 1.5 mmol, 153 μL, ρ = 1.044 g·mL^{-1}) according to the general procedure (24 h irradiation). The crude mixture was purified through column chromatography (SiO_2; cyclohexane/ethyl acetate 7:3) to afford **3a** (batch: 369 mg, 81% yield; flow: 160 mg; 90% yield) as a white solid (mp: 111-114 °C; *Lit.*[S3] 112-114 °C). Spectroscopic data of **3a** (^{1}H NMR and ^{13}C NMR) are in accordance with the literature.[S3]

^{1}H NMR (300 MHz, $CDCl_3$): δ 10.05 (bs, 1H), 8.00 (dt, *J* = 7.1, 1.4 Hz, 2 H), 7.65-7.56 (m, 1H), 7.49 (dt, *J* = 7.0, 1.2 Hz, 2H), 3.34 (t, *J* = 6.5 Hz, 2H), 2.84 (t, *J* = 6.5 Hz, 2H); ^{13}C NMR (75 MHz, $CDCl_3$): δ 197.5, 178.3, 136.1, 133.1, 128.4, 127.8, 32.9, 27.7. HRMS (ESI) m/z: [M-H]$^-$ calcd. for $C_{10}H_9O_3$ 177.0557, found 177.0553. IR (*neat*, ν/cm^{-1}): 2960, 1694, 1340, 1230, 612.

4-(4-methoxyphenyl)-4-oxobutanoic acid (3b)

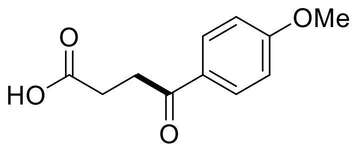

Prepared from fumaric acid **1a** (batch: 2.5 mmol, 290 mg; flow:1 mmol, 116 mg) and *p*-anisaldehyde **2b** (1.5 equiv.; batch: 3.75 mmol, 455 μL; flow: 1.5 mmol, 182 μL, ρ = 1.12 g·mL^{-1}) according to the general procedure (40 h irradiation). The crude mixture was purified through column chromatography (SiO_2; cyclohexane/ethyl acetate 7:3) to afford **3b** (batch: 369 mg, 81% yield; flow: 187 mg; 90% yield) as a white solid (mp: 64-66 °C; *Lit.*[S4] 64-65 °C). Spectroscopic data of **3b** (^{1}H NMR, ^{13}C NMR and IR) are in accordance with the literature.[S4]

^{1}H NMR (300 MHz, *acetone-d$_6$*): δ 10.12 (bs, 1H), 7.97 (d, *J* = 9 Hz, 2H), 7.00 (d, *J* = 9 Hz, 2H), 3.89 (s, 3H), 3.37 (t, *J* = 6.4 Hz, 2H), 2.69 (t, *J* = 6.4 Hz, 2H); ^{13}C NMR (75 MHz, *acetone-d$_6$*): 197.4, 174.5, 164.9, 131.3, 115.0, 56.3, 33.9, 28.7. IR (*neat*, ν/cm^{-1}): 2970, 1713, 1253, 615. HRMS (ESI) m/z: [M-H]$^-$ calcd. for $C_{11}H_{11}O_4$ 207.0663, found 207.0656.

4-(4-chlorophenyl)-4-oxobutanoic acid (3c)

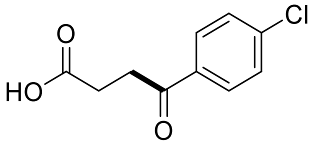

Prepared from fumaric acid **1a** (batch: 2.5 mmol, 290 mg; flow: 1 mmol, 116 mg) and *p*-chlorobenzaldehyde **2c** (1.5 equiv.; batch: 3.75 mmol, 529 mg; flow: 1.5 mmol, 210 mg) according to the general procedure (40 h irradiation). The crude mixture was purified through column chromatography (SiO_2; cyclohexane/ethyl acetate 7:3) to afford **3c** (batch: 440 mg, 83% yield, flow: 207 mg, 98% yield) as

a white solid (mp: 123-126 °C; *Lit.*[S5] 124-126 °C). Spectroscopic data of **3c** (^{1}H NMR, ^{13}C NMR and IR) are in accordance with the literature.[S5]

^{1}H NMR (300 MHz, *acetone-d$_6$*): δ 10.6 (bs, 1H), 7.97 (d, *J* = 9 Hz, 2H), 7.54 (d, *J* = 9 Hz, 2H), 3.31 (t, *J* = 6 Hz, 2H), 2.72 (t, *J* = 6 Hz, 2H), ^{13}C NMR (75 MHz, *acetone-d$_6$*): 197.8, 174.1, 139.6, 136.6, 130.7, 129.8, 34.1, 28.3; IR (*neat*, ν/cm^{-1}): 2970, 1712, 1590, 839. HRMS (ESI) m/z: [M-H]$^{-}$ calcd. for $C_{10}H_8ClO_3$ 211.0167, found 211.0159.

4-(4-bromophenyl)-4-oxobutanoic acid (3d)

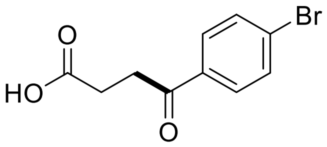

Prepared from fumaric acid **1a** (batch: 2.5 mmol, 290 mg; flow: 1 mmol, 116 mg) and *p*-bromobenzaldehyde **4d** (1.5 equiv.; batch: 3.75 mmol, 693 mg; flow: 1.5 mmol, 268 mg) according to the general procedure (40 h irradiation). The crude mixture was purified through column chromatography (SiO_2; cyclohexane/ethyl acetate 7:3) to afford **3d** (batch: 352 mg, 55% yield, flow: 195 mg, 76% yield) as a white solid (mp: 151-153 °C; *Lit.*[S6] 150-153 °C). Spectroscopic data of **3d** (^{1}H NMR and ^{13}C NMR) are in accordance with the literature.[S6]

^{1}H NMR (300 MHz, *acetone-d$_6$*): δ 10.5 (bs, 1H), 7.97 (d, *J* = 9 Hz, 2H), 7.72 (d, *J* = 9 Hz, 2H), 3.32 (t, *J* = 6.3 Hz, 2H), 2.71 (t, *J* = 6.4 Hz, 2H); ^{13}C NMR (75 MHz, *acetone-d$_6$*): 198.0, 174.1, 136.9, 132.8, 130.7, 128.3, 34.0, 28.2; IR (*neat*, ν/cm^{-1}): 2893 1730, 795. HRMS (ESI) m/z: [M-H]$^{-}$ calcd. for $C_{10}H_8BrO_3$ 254.9662, found 254.9655.

4-(4-fluorophenyl)-4-oxobutanoic acid (3e)

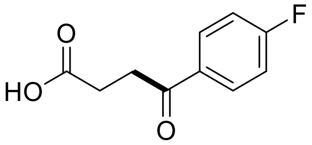

Prepared from fumaric acid **1a** (batch: 2.5 mmol, 290 mg; flow: 1 mmol, 116 mg) and *p*-fluorobenzaldehyde **2e** (1.5 equiv.; batch: 3.75 mmol, 465 mg ; flow: 1,5 mmol, 186 mg) according to the general procedure (24 h irradiation). The crude mixture was purified through column chromatography (SiO_2; cyclohexane/ethyl acetate 7:3) to afford **3e** (batch: 255 mg, 52% yield, flow: 143 mg, 73% yield) as a white solid (mp: 101-102 °C; *Lit.*[S7] 102.5-103 °C). Spectroscopic data of **3e** (^{1}H NMR and ^{13}C NMR) are in accordance with the literature.[S7]

^{1}H NMR (300 MHz, $CDCl_3$): δ 9.98 (bs, 1H), 8.19-8.04 (m, 2H), 7.32-7.12 (m, 2H), 3.41 (t, *J* = 6.5 Hz, 2H), 2.93 (t, *J* = 6.5 Hz, 2H). ^{13}C NMR (75 MHz, $CDCl_3$) δ 196.9, 179.5, 166.4 (d, *J* = 254 Hz), 133.3, 131.3 (d, *J* = 9 Hz), 116.3 (d, *J* = 22 Hz), 33.6, 28.6; IR (*neat*, ν/cm^{-1}): 2969, 1713, 1665, 1605, 1560, 1424, 1255, 1192, 1027, 619. HRMS (ESI) m/z: [M-H]$^{-}$ calcd. for $C_{10}H_8FO_3$ 195.0306, found 195.0302.

4-oxo-4-(4-(trifluoromethyl)phenyl)butanoic acid (3f)

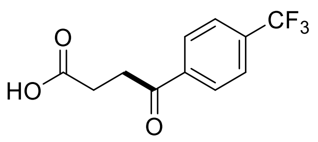

Prepared from fumaric acid **1a** (batch: 2.5 mmol, 290 mg; flow: 1 mmol, 116 mg) and *p*-(trifluoromethyl)benzaldehyde **2f** (1.5 equiv.; batch: 3.75 mmol, 653 mg; flow: 1.5 mmol, 261 mg), according to the general procedure (24 h irradiation). The crude mixture was purified through column chromatography (SiO_2; cyclohexane/ethyl acetate 7:3) to afford **3f** (batch: 590 mg, 96% yield, flow: 204 mg, 83% yield) as a white solid (mp: 128−130 °C). Spectroscopic data of **3f** (^{1}H NMR and ^{13}C NMR) are in accordance with the literature.[S8]

^{1}H NMR (300 MHz, *acetone-d$_6$*) δ 10.5 (bs, 1H), 8.24 (d, *J* = 8.2 Hz, 2H), 7.89 (d, *J* = 8.2 Hz, 2H), 3.40 (t, *J* = 6.3 Hz, 2H), 2.77 (dd, *J* = 6.8, 5.9 Hz, 2H); ^{13}C NMR (75 MHz, *acetone-d$_6$*) δ 198.3, 174.0, 134.4 (q, *J* = 32 Hz), 127 (q, *J* = 3.7 Hz), 125.4 (q, *J* = 338 Hz), 34.4, 28.3; IR (*neat*, ν/cm^{-1}): 3087, 1712, 686. HRMS (ESI) m/z: [M-H]$^-$ calcd. for $C_{11}H_8F_3O_3$ 245.9655, found 245.0420.

4-oxo-4-(4-tolyl)butanoic acid (3g)

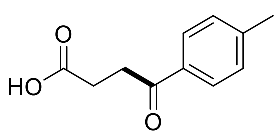

Prepared from fumaric acid **1a** (batch: 2.5 mmol, 290 mg; flow: 1 mmol, 116 mg) and *p*-tolualdehyde **2g** (1.5 equiv.; batch: 3.75 mmol, 442 mg; flow: 1.5 mmol, 180 mg) according to the general procedure (24 h irradiation). The crude mixture was purified through column chromatography (SiO_2; cyclohexane/ethyl acetate 7:3) to afford **3g** (batch: 422 mg, 88% yield, flow: 144 mg, 75%) as a white solid (mp: 130-131 °C; *Lit.*[S6] 130-132 °C). Spectroscopic data of **3g** (^{1}H NMR) are in accordance with the literature.[6]

^{1}H NMR (300 MHz, $CDCl_3$): δ 7.88 (d, *J* = 7.0 Hz, 2H), 7.22 (d, *J* = 7.0 Hz, 2H), 3.30 (t, *J* = 6.6 Hz, 2H), 2.82 (t, *J* = 6.6 Hz, 2H), 2.42 (s, 3H); ^{13}C NMR (75 MHz, $CDCl_3$): 197.2, 178.1, 143.9, 133.7, 129.1, 127.9, 32.8, 27.8, 21.4. IR (*neat*, ν/cm^{-1}): 3012, 1745, 1713, 1450, 1030, 630. HRMS (ESI) m/z: [M-H]$^-$ calcd. for $C_{11}H_{11}O_3$ 191.0714, found 191.0708.

4-(4-ethylphenyl)-4-oxobutanoic acid (3h)

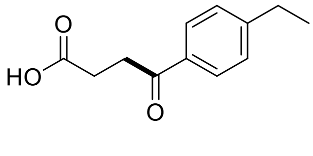

Prepared from fumaric acid **1a** (batch: 2.5 mmol, 290 mg; flow: 1 mmol, 116 mg) and *p*-ethylbenzaldehyde **2h** (1.5 equiv.; batch: 3.75 mmol, 502 μL; flow: 1.5 mmol, 180 μL, ρ = 1.001 g·mL^{-1}) according to the general procedure (24 h irradiation). The crude mixture was purified through column chromatography (SiO_2; cyclohexane/ethyl acetate 7:3) to afford **3h** (batch: 433 mg, 84% yield, flow: 134 mg, 65% yield) as a white solid (mp: 109-112 °C; *Lit.*[S9] 110-112 °C). Spectroscopic data of **3h** (^{1}H NMR, ^{13}C NMR and IR) are in accordance with the literature.[S9]

^{1}H NMR (300 MHz, $CDCl_3$): δ 10.65 (bs, 1H), 7.89 (d, *J* = 7.9 Hz, 2H), 7.27 (d, *J* = 8.0 Hz, 2H), 3.28 (t, *J* = 6.5 Hz, 2H), 2.87-2.61 (m, 4H), 1.24 (t, *J* = 7.6 Hz, 3H); ^{13}C NMR (75 MHz, $CDCl_3$): δ

197.2, 178.7, 150.0, 133.8, 128.0, 127.8, 32.7, 28.6, 27.8, 14.9. IR (*neat*, ν/cm^{-1}): 3008,1765, 1467, 1246, 997, 625. HRMS (ESI) m/z: [M-H]$^{-}$ calcd for $C_{12}H_{13}O_3$ 205.0870, found 205.0864.

4-([1,1'-biphenyl]-4-yl)-4-oxobutanoic acid (3i)

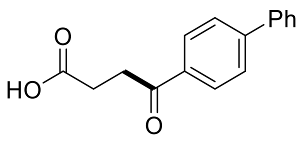

Prepared from fumaric acid **1a** (batch: 2.5 mmol, 290 mg; flow: 1 mmol, 116 mg) and biphenyl-4-carboxaldehyde **2i** (1.5 equiv.; batch: 3.75 mmol, 683 mg; flow: 1.5 mmol, 273 mg) according to the general procedure (24 h irradiation). The crude mixture was purified through column chromatography (SiO_2; cyclohexane/ethyl acetate 8:2) to afford **3i** (batch: 347 mg, 53% yield, flow: 191 mg, 75%) as a yellowish oil. Spectroscopic data of **3i** (^{1}H NMR and ^{13}C NMR) are in accordance with the literature.[S10]

^{1}H NMR (300 MHz, *acetone-d$_6$*): δ 10.64 (bs, 1H), 8.13 (d, *J* = 8.7 Hz, 2H), 7.82 (dd, *J* = 9.0 Hz, 2H), 7.74 (d, *J* = 8.8 Hz, 2H), 7.56 (t, *J* = 6.0 Hz, 2H), 7.43 (t, *J* = 6.0 Hz, 1H), 3.37 (t, *J* = 6.7 Hz, 2H), 2.75 (t, *J* = 6.9, Hz, 2H); ^{13}C NMR (75 MHz, Acetone-*d$_6$*): δ 198.4, 174.2, 146.3, 140.8, 136.8, 130.0, 129.6, 129.2, 128.1, 128.0, 34.1, 28.4. IR (*neat*, ν/cm^{-1}): 3013 ,1733, 623. HRMS (ESI) m/z: [M-H]$^{-}$ calcd. for $C_{16}H_{13}O_3$ 253.0870, found 253.0863.

4-oxo-4-(3,4,5-trimethoxyphenyl)butanoic acid (3j)

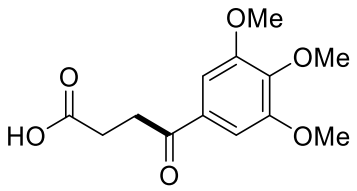

Prepared from fumaric acid **1a** (batch: 2.5 mmol, 290 mg; flow: 1 mmol, 116 mg) and 3,4,5-trimethoxybenzaldehyde **2j** (1.5 equiv.; batch: 3.75 mmol, 538 μL; flow: 1.5 mmol, 215 μL, ρ = 1.37 g·mL^{-1}) according to the general procedure (24 h irradiation). The crude mixture was purified through column chromatography (SiO_2; cyclohexane/ethyl acetate 7:3) to afford **3j** (batch: 268 mg, 54% yield; flow: 182 mg; 68% yield) as a white solid (mp:116-118 °C; *Lit.*[S11] 117-118 °C). Spectroscopic data of **2j** (IR) are in accordance with the literature.[11]

^{1}H NMR (300 MHz, *acetone-d$_6$*): δ 7.34 (s, 2H), 3.90 (s, 6H), 3.80 (s, 3H), 3.30 (t, *J* = 6.8 Hz, 2H), 2.69 (t, *J* = 6.8 Hz, 2H); ^{13}C NMR (75 MHz, *acetone-d$_6$*): 197.6, 174.2, 154.2, 143.6, 133.1, 106.5, 60.7, 56.6, 33.8, 28.4. IR (*neat*, ν/cm^{-1}): 3023 ,1706, 1640, 1325. HRMS (ESI) m/z: [M-H]$^{-}$ calcd. for $C_{13}H_{15}O_6$ 267.0874, found 267.0863.

4-oxo-6-phenylhexanoic acid (3k)

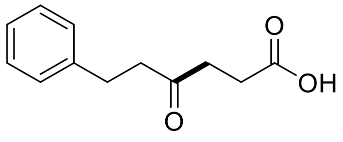

Prepared from fumaric acid **1a** (batch: 2.5 mmol, 290 mg; flow: 1 mmol, 116 mg) and hydrocinnamaldehyde **2k** (1.5 equiv.; batch: 3.75 mmol, 493 μL; flow: 1.5 mmol, 197 μL, ρ = 1.019 g·mL^{-1}) according to the general procedure (24 h irradiation). The crude mixture was purified through column chromatography (SiO_2; cyclohexane/ethyl acetate 7:3) to afford **3k** (batch: 438 mg, 89% yield; flow: 187 mg; 91%

yield) as a yellowish solid (mp: 87-88 °C; *Lit.*[12] 87.3-88.0 °C). Spectroscopic data of **3k** (^{1}H NMR, ^{13}C NMR and IR) are in accordance with the literature.[S12]

^{1}H NMR (300 MHz, $CDCl_3$): δ 7.31-7.22 (m, 2H), 7.20-7.12 (m, 3H), 2.95-2.86 (m, 2H), 2.82-2.73 (m, 2H), 2.70-2.69 (m, 2H), 2.64-2.58 (m, 2H); ^{13}C NMR (75 MHz, $CDCl_3$): δ 207.5, 177.8, 140.6, 128.2, 128.0, 125.9, 43.9, 36.7, 29.4, 27.4. IR (*neat*, ν/cm^{-1}): 2964, 2920, 2851, 1694, 1374, 673. HRMS (ESI) m/z: [M-H]$^-$ calcd. for $C_{12}H_{13}O_3$ 205.0870, found 205.0860.

4-oxodecanoic acid (3l)

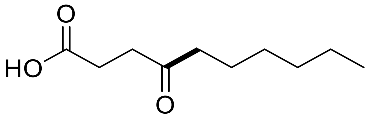

Prepared from fumaric acid **1a** (batch: 2.5 mmol, 290 mg; flow: 1 mmol, 116 mg) and heptaldehyde **3l** (1.5 equiv.; batch: 3.75 mmol, 523 μL; flow: 1.5 mmol, 209 μL, ρ = 0.817 g·mL^{-1}) according to the general procedure (24 h irradiation). The crude mixture was purified through column chromatography (SiO_2; cyclohexane/ethyl acetate 7:3) to afford **3l** (batch: 320 mg, 72% yield; flow: 178 mg; 95% yield) as a white solid (mp: 65-67 °C; *Lit.*[S13] 66-67 °C). Spectroscopic data of **3l** (^{1}H NMR and ^{13}C NMR) are in accordance with the literature.[S14]

^{1}H NMR (300 MHz, $CDCl_3$): δ 2.76-2.67 (m, 2H), 2.65-2.55 (m, 2H), 2.44 (t, J = 7.5 Hz, 2H), 1.62-1.51 (m, 2H), 1.38-1.13 (m, 6H), 0.87 (t, J = 7.5 Hz, 3H); ^{13}C NMR (75 MHz, $CDCl_3$): δ 208.9, 178.3, 42.6, 36.6, 31.4, 28.7, 27.6, 23.7, 22.4, 13.9. IR (*neat*, ν/cm^{-1}): 3050, 1770, 1420, 1265, 750. HRMS (ESI) m/z: [M-H]$^-$ calcd. for $C_{10}H_{17}O_3$ 185.1183, found 185.1179.

4-oxoundecanoic acid (3m)

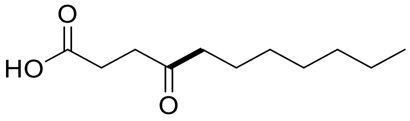

Prepared from fumaric acid **1a** (batch: 2.5 mmol, 290 mg; flow: 1 mmol, 116 mg) and octanal **2m** (1.5 equiv.; batch: 3.75 mmol, 591 μL; flow: 1.5 mmol, 234 μL, ρ = 0.821 g·mL^{-1}) according to the general procedure (40 h irradiation). The crude mixture was purified through column chromatography (SiO_2; cyclohexane/ethyl acetate 7:3) to afford **2m** (batch: 280 mg, 56% yield; flow: 186 mg; 93% yield) as a white solid (mp: 78-80 °C; *Lit.*[13] 79-80 °C). Spectroscopic data of **3m** (^{1}H NMR and ^{13}C NMR) are in accordance with the literature.[S14]

^{1}H NMR (300 MHz, $CDCl_3$): δH δ 2.76-2.68 (m, 2H), 2.66-2.58 (m, 2H), 2.44 (t, J = 7.5 Hz, 2H), 1.65-1.52 (m, 2H), 1.38-1.17 (m, 8H), 0.84 (t, J = 7.0 Hz, 3H); ^{13}C NMR (75 MHz, $CDCl_3$): δ 209.6, 179.0, 43.3, 37.3, 32.2, 29.7, 29.6, 28.3, 24.4, 23.2, 14.6. IR (*neat*, ν/cm^{-1}): 2950, 2935, 1706, 1370, 1245, 650. HRMS (ESI) m/z: [M-H]$^-$ calcd. for $C_{11}H_{19}O_3$ 199.1340, found 199.1334.

4-oxododecanoic acid (3n)

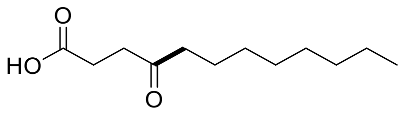

Prepared from fumaric acid **1a** (batch: 2.5 mmol, 290 mg; flow: 1 mmol, 116 mg) and nonanal **2n** (1.5 equiv.; batch: 3.75 mmol, 644 µL; flow: 1.5 mmol, 258 µL, ρ= 0.827 g·mL^{-1}) according to the general procedure (40 h irradiation). The crude mixture was purified through column chromatography (SiO_2; cyclohexane/ethyl acetate 7:3) to afford **3n** (batch: 342 mg, 64% yield; flow: 197 mg; 92% yield) as a white solid (mp: 90-92 °C).

^{1}H NMR (300 MHz, $CDCl_3$): δ 2.75-2.66 (m, 2H), 2.65-2.57 (m, 2H), 2.43 (t, *J* = 7.5 Hz, 2H), 1.63-1.51 (m, 2H), 1.36-1.13 (m, 10H), 0.87 (t, *J* = 7.0 Hz, 3H); ^{13}C NMR (75 MHz, $CDCl_3$): δ 208.7, 178.1, 42.5, 36.5, 31.5, 29.1, 28.9, 28.8, 27.5, 23.5, 22.4, 13.8. IR (*neat*, ν/cm^{-1}): 2974, 1710, 1345, 1230, 655. HRMS (ESI) m/z: [M-H]$^-$ calcd. for $C_{12}H_{21}O_3$ 213.1496, found 213.1491.

2-(4-cyano-2-methylbutan-2-yl)succinic acid (3o)

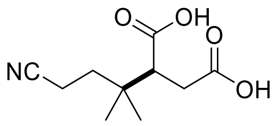

Prepared from fumaric acid **1a** (batch: 2.5 mmol, 290 mg; flow: 1 mmol, 116 mg) and isocapronitrile **2o** (10 equiv.; batch: 25 mmol, 3.03 mL, flow: 10 mmol, 1.23 mL, ρ = 0.8 g·mL^{-1}) according to the general procedure (24 h irradiation). The crude mixture was purified through column chromatography (SiO_2; cyclohexane/ethyl acetate 8:2) to afford **3o** (batch: 282 mg, 53% yield, flow: 185 mg, 87% yield) as a yellowish oil.

^{1}H NMR (300 MHz, *acetone-d*$_6$) δ 9.96 (bs, 2H), 2.79-2.64 (m, 2H), 2.58-2.40 (m, 3H), 1.85-1.64 (m, 2H), 1.04 (s, 3H), 0.98 (s, 3H). ^{13}C NMR (75 MHz, *acetone-d*$_6$): δ 174.3, 173.3, 120.0, 48.4, 35.8, 34.1, 31.4, 23.7, 23.5, 11.3. IR (*neat*, ν/cm^{-1}): 3009, 2350, 1720. HRMS (ESI) m/z: [M-H]$^-$ calcd. for $C_{10}H_{14}NO_4$ 212.0928, found 212.0920.

2-(3-oxocyclopentyl)succinic acid (3p)

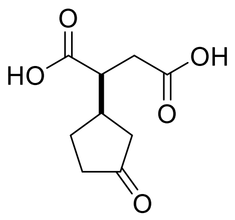

Prepared from fumaric acid **1a** (batch: 2.5 mmol, 290 mg; flow: 1 mmol, 116 mg) and cyclopentanone **2p** (10 equiv.; batch: 25 mmol, 2.21 mL, flow: 10 mmol, 884 mL, ρ = 0.95 g·mL^{-1}) according to the general procedure (40 h irradiation). The crude mixture was purified through column chromatography (SiO_2; cyclohexane/ethyl acetate 8:2) to afford **3p** (*ca*. 1:1 mixture of diastereoisomers; batch: 339 mg, 66% yield, flow: 164 mg, 82% yield) as a white solid (mp: 115-118 °C).

^{1}H NMR (300 MHz, *acetone-d*$_6$): δ 9.06 (bs, 2H), 2.90-2.64 (m, 2H), 2.62-2.00 (m, 7H), 1.87-1.57 (m, 1H). ^{13}C NMR (75 MHz, *acetone-d*$_6$): δ 216.8, 216.7, 175.2, 175.1, 173.3, 173.2, 46.0, 45.9, 43.2, 42.9, 39.5, 39.2, 38.6, 38.5, 35.0, 34.4, 27.9, 27.7. IR (*neat*, ν/cm^{-1}): 2982 ,1712, 1290. HRMS (ESI) m/z: [M-H]$^-$ calcd. for $C_9H_{11}O_5$ 199.0612, found 199.0604.

2-(1,3-benzodioxol-2-yl)succinic acid (3q)

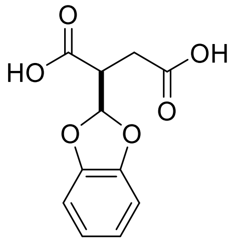

Prepared from fumaric acid **1a** (batch: 2.5 mmol, 290 mg; flow: 1 mmol, 116 mg) and 1,3-benzodioxole **2q** (1.5 equiv.; batch: 3.75 mmol, 0.429 mL, flow: 1.5 mmol, 172 mL, ρ = 1.064 g·mL^{-1}) according to the general procedure (24 h irradiation). The crude mixture was purified through column chromatography (SiO_2; cyclohexane/ethyl acetate 8:2) to afford **3q** (batch: 583 mg, 98% yield; flow: 233 mg, 98% yield) as a white solid (mp: 180-183 °C).

^{1}H NMR (300 MHz, *acetone-d*$_6$) δ 10.9 (bs, 2H), 6.85 (s, 4H), 6.54 (d, *J* = 3.9 Hz, 1H), 3.51 (dt, *J* = 9.5, 4.0 Hz, 1H), 2.83 (dd, *J* = 17.0, 9.5 Hz, 1H), 2.65 (dd, *J* = 17.0, 4.1 Hz, 1H); ^{13}C NMR (75 MHz, *acetone-d*$_6$): δ 172.6, 170.9, 148.2, 122.7, 110.6, 109.3, 47.1, 29.7. IR (*neat*, ν/cm^{-1}): 3012,1750, 630. HRMS (ESI) m/z: [M-H]$^-$ calcd. for $C_{11}H_9O_6$ 237.0405, found 237.0398.

2-cyclopentylsuccinic acid (3r)

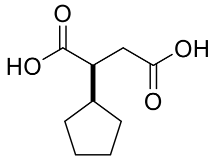

Prepared from fumaric acid **1a** (batch: 2.5 mmol, 290 mg; flow: 1 mmol, 116 mg) and cyclopentane **2r** (5 equiv.; batch: 12.5 mmol, 1.17 mL; flow: 5 mmol, 467 mL, ρ = 0.75 g·mL^{-1}) according to the general procedure (24 h irradiation). The crude mixture was purified through column chromatography (SiO_2; cyclohexane/ethyl acetate 7:3) to afford **3r** (batch: 74 mg, 16% yield, flow: 47 mg, 25% yield) as a yellowish solid (mp: 111-113 °C; *Lit.*[S15] 112.5-115 °C). Spectroscopic data of **3r** (^{1}H NMR and ^{13}C NMR) are in accordance with the literature.[S16]

^{1}H NMR (300 MHz, $CDCl_3$): δ 10.28 (bs, 2H), 2.74-2.60 (m, 2H), 2.58-2.41 (m, 1H), 2.12-1.94 (m, 2H), 1.87-1.16 (m, 7H). ^{13}C NMR (75 MHz, $CDCl_3$): δ 176.1, 173.6, 46.4, 43.0, 35.6, 31.0, 30.9, 25.6, 25.5. IR (*neat*, ν/cm^{-1}): 2998, 1726, 684. HRMS (ESI) m/z: [M-H]$^-$ calcd. for $C_9H_{13}O_4$ 185.0819, found 185.0813.

2-cyclohexylsuccinic acid (3s)

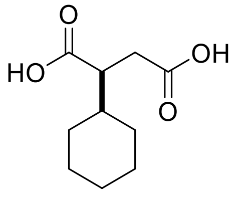

Prepared from fumaric acid **1a** (batch: 2.5 mmol, 290 mg; flow: 1 mmol, 116 mg) and cyclohexane **2s** (5 equiv.; batch: 12.5 mmol, 1.34 mL; flow: 10 mmol, 1.08 mL, ρ = 0.78 g·mL^{-1}) according to the general procedure (24 h irradiation). The crude mixture was purified through column chromatography (SiO_2; cyclohexane/ethyl acetate 7:3) to afford **3s** (batch: 305 mg, 61% yield; flow: 148 mg, 74% yield) as a yellowish solid (mp: 142-144 °C).

^{1}H NMR (300 MHz, *acetonitrile-d*$_3$): δ 8.89 (bs, 2H), 2.69-2.34 (m, 3H), 1.79-1.52 (m, 6H), 1.35-0.97 (m, 5H).^{13}C NMR (75 MHz, *acetonitrile-d*$_3$): δ 176.1, 174.3, 47.4, 40.6, 33.4, 31.3, 30.7, 27.1,

26.9. IR (*neat*, ν/cm^{-1}): 3015, 1735, 889. HRMS (ESI) m/z: [M-H]$^{-}$ calcd. for $C_{10}H_{15}O_4$ 199.0976, found 199.0969.

2-cycloheptylsuccinic acid (3t)

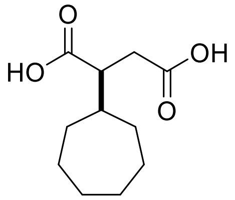

Prepared from fumaric acid **1a** (batch: 2.5 mmol, 290 mg) and cycloheptane **2t** (10 equiv.; batch: 25 mmol, 3.02 mL, ρ = 0.81 g·mL^{-1}) according to the general procedure (40 h irradiation), while adopting a MeCN/CH_2Cl_2 5:1 mixture as reaction medium. The crude mixture was purified through column chromatography (SiO_2; cyclohexane/ethyl acetate 7:3) to afford **3t** (batch: 251 mg, 47% yield) as a white solid (mp: 119-120 °C).

^{1}H NMR (300 MHz, *acetone-d$_6$*) δ 10.75 (bs, 2H), 2.80 (dt, *J* = 10.6, 3.9 Hz, 1H), 2.67 (dd, *J* = 16.6, 10.6 Hz, 1H), 2.40 (dd, *J* = 16.6, 3.5 Hz, 1H), 1.99-1.86 (m, 1H), 1.78-1.24 (m, 12H). ^{13}C NMR (75 MHz, *acetone-d$_6$*): δ 175.7, 174.0, 48.1, 42.0, 33.0, 32.8, 31.8, 28.7, 28.6, 27.7. IR (*neat*, ν/cm^{-1}): 2922, 1692, 913. HRMS (ESI) m/z: [M-H]$^{-}$ calcd. for $C_{11}H_{17}O_4$ 213.1132, found 213.1123.

2-cyclooctylsuccinic acid (3u)

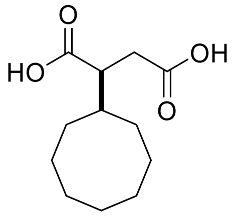

Prepared from fumaric acid **1a** (batch: 2.5 mmol, 290 mg) and cyclooctane **2u** (10 equiv.; batch: 25 mmol, 3.33 mL, ρ = 0.83 g·mL^{-1}) according to the general procedure (40 h irradiation), while adopting a MeCN/CH_2Cl_2 5:1 mixture as reaction medium. The crude mixture was purified through column chromatography (SiO_2; cyclohexane/ethyl acetate 7:3) to afford **3u** (batch: 376 mg, 66% yield) as a white solid (mp: 126-128 °C).

^{1}H NMR (300 MHz, *acetone-d$_6$*) δ 9.45 (bs, 2H), 2.85-2.61 (m, 2H), 2.41 (dd, *J* = 16.1, 3.7 Hz, 1H), 2.05-1.95 (m ,1H), 1.72-1.40 (m, 14 H); ^{13}C NMR (75 MHz, *acetone-d$_6$*): δ 175.8, 174.0, 48.5, 39.6, 32.8, 31.7, 30.4, 27.1, 27.1, 27.0, 26.6, 26.6. IR (*neat*, ν/cm^{-1}): 2956, 1703. HRMS (ESI) m/z: [M-H]$^{-}$ calcd. for $C_{12}H_{19}O_4$ 227.1289, found 227.1278.

2-(2,3-dimethylbutan-2-yl)succinic acid (3v)

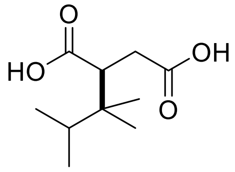

Prepared from fumaric acid **1a** (batch: 2.5 mmol, 290 mg) and 2,3 -dimethylbutane **2v** (20 equiv.; batch: 50 mmol, 6.50 mL, ρ = 0.66 g·mL^{-1}) according to the general procedure (34 h irradiation), while adopting a MeCN/CH_2Cl_2 5:1 mixture as reaction medium. The crude mixture was purified through column chromatography (SiO_2; cyclohexane/ethyl acetate 5:5) to afford **3v** (batch: 335 mg, 66% yield) as a white solid (mp: 125-127 °C; *Lit.*[S17]130 °C).

^{1}H NMR (300 MHz, *acetone-d$_6$*): δ 10.32 (bs, 2H), 2.84 (dd, *J* = 11.5, 3.1 Hz, 1H), 2.60 (dd, *J* = 16.7, 11.5 Hz, 1H), 2.41 (dd, *J* = 16.8, 3.1 Hz, 1H), 0.84 (d, *J* = 6.7 Hz, 3H), 0.81 (s, 3H), 0.80 (s, 3H),

0.77 (d, J=6.5 Hz, 3H); ^{13}C NMR (75 MHz, *acetone-d$_6$*): δ 175.9, 174.2, 48.6, 37.8, 34.8, 21.1, 17.8, 17.7. IR (*neat*, ν/cm^{-1}): 2959, 1697, 924, 682. HRMS (ESI) m/z: [M-H]$^-$ calcd. for $C_{10}H_{17}O_4$ 201.1132, found 202.1131.

2-methyl-4-oxo-4-phenylbutanoic acid (4a)

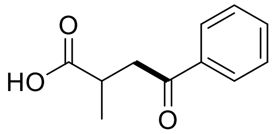

Prepared from citraconic acid **1b** (batch: 2.5 mmol, 325 mg; flow: 1 mmol, 130 mg) and benzaldehyde **2a** (1.5 equiv.; batch: 3.75 mmol, 380 mL; flow: 1.5 mmol, 153 mL, ρ = 1.044 g·mL^{-1}) according to the general procedure (24 h irradiation). The crude mixture was purified through column chromatography (SiO_2; cyclohexane/ethyl acetate 7:3) to afford **4a** (batch: 366 mg, 76% yield, flow: 180 mg, 94% yield) as a white solid (mp: 132-134 °C). Spectroscopic data of **4a** (^{1}H NMR and ^{13}C NMR) are in accordance with the literature.[S18]

^{1}H NMR (300 MHz, $CDCl_3$) δ 10.33 (bs, 1H), 7.97 (d, *J* = 7.2 Hz, 2H), 7.57 (t, *J* = 7.1 Hz, 1H), 7.47 (t, *J* = 7.2 Hz, 2H), 3.48 (dd, *J* = 17.5, 7.5 Hz, 1H), 3.23-3.13 (m, 1H), 3.06 (dd, *J* = 17.6, 5.2 Hz, 1H), 1.32 (d, *J* = 7.1 Hz, 3H); ^{13}C NMR (75 MHz, $CDCl_3$) δ 198.4, 182.2, 137.1, 133.9, 129.2, 128.6, 42.3, 35.3, 17.7. IR (*neat*, ν/cm^{-1}): 3011, 1705, 1344, 606. HRMS (ESI) m/z: [M-H]$^-$ calcd. for $C_{11}H_{11}O_3$ 191.0714, found 191.0711.

4-(4-methoxyphenyl)-2-methyl-4-oxobutanoic acid (4b)

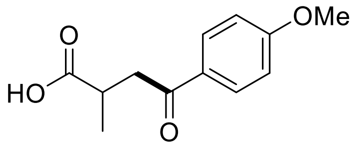

Prepared from citraconic acid **1b** (batch: 2.5 mmol, 325 mg; flow: 1 mmol, 130 mg) and *p*-anisaldehyde **2b** (1.5 equiv; batch: 3.75 mmol, 455 μL; flow: 1.5 mmol, 182 μL, ρ = 1.12 g·mL^{-1}) according to the general procedure (24 h irradiation). The crude mixture was purified through column chromatography (SiO_2; cyclohexane/ethyl acetate 7:3) to afford **4b** (batch: 455 mg, 82% yield; flow: 189 mg, 85% yield) as a white solid (mp:142-144 °C). Spectroscopic data of **4b** (^{1}H NMR and ^{13}C NMR) are in accordance with the literature.[S18]

^{1}H NMR (300 MHz, $CDCl_3$): δ 7.94 (d, *J* = 10.7 Hz, 2H), 6.93 (d, *J* = 10.7 Hz, 2H), 3.87 (s, 3H), 3.41 (dd, *J* = 17.4, 7.6 Hz, 1H), 3.21-3.08 (m, 1H), 3.02 (dd, *J* = 17.4, 5.4 Hz, 1H), 1.31 (d, *J* = 7.1 Hz, 3H); ^{13}C NMR (75 MHz, $CDCl_3$) δ 197.1, 181.5, 164.3, 130.9, 130.2, 114.4, 56.1, 42.0, 35.3, 17.7. IR (*neat*, ν/cm^{-1}): 2976, 1709. HRMS (ESI) m/z: [M-H]$^-$ calcd. for $C_{12}H_{13}O_4$ 221.0819, found 221.0890.

4-(4-chlorophenyl)-2-methyl-4-oxobutanoic acid (4c)

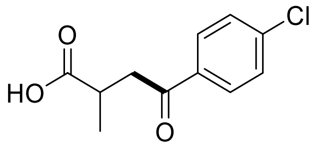

Prepared from citraconic acid **1b** (batch: 2.5 mmol, 325 mg; flow: 1 mmol, 130 mg) and *p*-chlorobenzaldehyde **2c** (1.5 equiv.; batch: 3.75 mmol, 529 mg; flow: 1.5 mmol, 210 mg) according to the general procedure (24 h

irradiation). The crude mixture was purified through column chromatography (SiO_2; cyclohexane/ethyl acetate 7:3) to afford **4c** (batch: 254 mg, 78% yield; flow: 221 mg, 98% yield) as a white solid (mp: 136-138 °C). Spectroscopic data of **4c** (^{1}H NMR and ^{13}C NMR) are in accordance with the literature.[S18]

^{1}H NMR (300 MHz, *acetone-d*$_6$): δ 10.63 (bs, 1H), 8.02 (d, J = 8.9 Hz, 2H), 7.55 (d, J = 8.9 Hz, 2H), 3.57-3.39 (m, 1H), 3.18-2.95 (m, 2H), 1.28 (d, J = 7.0 Hz, 3H); ^{13}C NMR (75 MHz, *acetone-d*$_6$): δ 197.7, 177.1, 139.6, 136.6, 130.7, 129.7, 42.6, 35.5, 17.6. IR (*neat*, ν/cm^{-1}): 3009, 1704, 1645, 645. HRMS (ESI) m/z: [M-H]$^-$ calcd. for $C_{11}H_{10}ClO_3$ 225.0324, found 225.0320.

2-methyl-4-oxo-4-(3,4,5-trimethoxyphenyl)butanoic acid (4j)

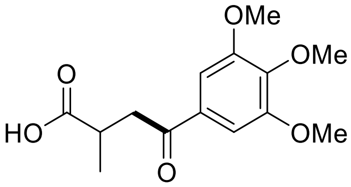

Prepared from citraconic acid **1b** (batch: 2.5 mmol, 325 mg; flow: 1 mmol, 130 mg) and 3,4,5-trimethoxybenzaldehyde **2j** (1.5 equiv.; batch: 3.75 mmol, 538 μL; flow: 1.5 mmol, 215 μL, ρ = 1.37 g·mL^{-1}) according to the general procedure (24 h irradiation). The crude mixture was purified through column chromatography (SiO_2; cyclohexane/ethyl acetate 7:3) to afford **4j** (batch: 627 mg, 89% yield; flow: 262 mg, 93% yield) as a white solid (mp: 49-51 °C).

^{1}H NMR (300 MHz, *acetone-d*$_6$): δ 10.72 (bs, 1H), 7.32 (s, 2H), 3.90 (s, 6H), 3.80 (s, 3H), 3.55-3.40 (m, 1H), 3.14-2.98 (m, 2H), 1.26 (d, J = 7.1 Hz, 3H); ^{13}C NMR (75 MHz, *acetone-d*$_6$): δ 197.5, 177.3, 154.4, 143.7, 133.3, 106.6, 60.8, 56.7, 42.5, 35.6, 17.7. IR (*neat*, ν/cm^{-1}): 3009 ,1704, 1645, 645. HRMS (ESI) m/z: [M-H]$^-$ calcd. for $C_{14}H_{17}O_6$ 181.1031, found 181.1025.

Reaction between hydrocinnamaldehyde and citraconic acid

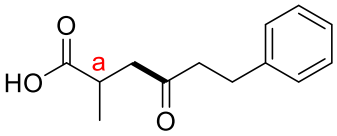

4k

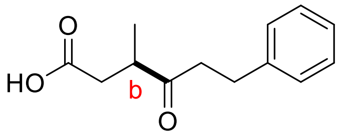

4k'

4k:4k' = 3:1

Prepared from citraconic acid **1b** (batch: 2.5 mmol, 325 mg; flow: 1 mmol, 130 mg) and hydrocinnamaldehyde **2k** (1.5 equiv.; batch: 3.75 mmol, 493 μL; flow: 1.5 mmol, 197 μL, ρ = 1.019 g·mL^{-1}) according to the general procedure (24 h irradiation). The crude mixture was purified through column chromatography (SiO_2; cyclohexane/ethyl acetate 7:3) to afford a mixture of 2-methyl-4-oxo-6-phenylhexanoic acid (**4k**) and 3-methyl-4-oxo-6-phenylhexanoic acid (**4k'**) in a **4k**:**4k'** 3:1 ratio (batch: 438 mg, 89% yield; flow: 200 mg; 91%) as a yellowish oil. DEPT analysis unambiguously allowed to identify the structures of regioisomeric **4k** and **4k'**, based on the chemical shift of carbon atoms labelled as **a** and **b** (see structures and inset in the C-NMR spectrum).

4k: ^{1}H NMR (300 MHz, $CDCl_3$, from the mixture): δ 11.52 (bs, 1H), 7.42-7.22 (m, 5H), 3.15-2.79 (m, 6H), 2.52 (dd, J = 17.6, 5.2 Hz, 1H), 1.27 (d, J = 7.1 Hz, 3H); ^{13}C NMR (75 MHz, $CDCl_3$, from the mixture): 207.5, 181.6, 140.4, 128.1, 127.9 125.7, 45.1, 43.8, 34.1, 29.2, 16.4.

4k': ^{1}H NMR (300 MHz, $CDCl_3$, from the mixture): δ 11.52 (bs, 1H), 7.42-7.22 (m, 5H), 3.15-2.79 (m, 6H) 2.41 (dd, J = 17.0, 5.0 Hz, 1H), 1.16 (d, J = 7.1 Hz, 3H); ^{13}C NMR (75 MHz, $CDCl_3$, from the mixture): 211.4, 178.0, 140.7, 128.0, 127.9, 125.6, 42.2, 41.5, 36.2, 29.1, 16.0.
IR of the mixture (*neat*, ν/cm^{-1}): 3103, 1706, 630.
HRMS of the mixture (ESI) m/z: [M-H]$^{-}$ calcd. for $C_{13}H_{15}O_3$ 219.1027, found 219.1019.

2-(4-cyano-2-methylbutan-2-yl)-3-methylsuccinic acid (4o)

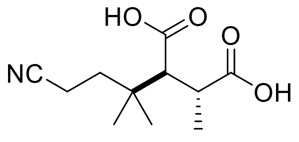

(±)-**4o**

Prepared from citraconic acid **1b** (batch: 2.5 mmol, 325 mg; flow: 1 mmol, 130 mg) and isocapronitrile **2o** (10 equiv.; batch: 25 mmol, 3.03 mL, flow: 10 mmol, 1.23 mL, ρ = 0.8 g·mL^{-1}) according to the general procedure (40 h irradiation). The crude mixture was purified through column chromatography (SiO_2; cyclohexane/ethyl acetate 7:3) to afford **4o** (batch: 494 mg, 87% yield; flow: 211 mg, 93% yield) as a white solid (mp: 135-138 °C). The process delivered a single diastereoisomer, that has been assigned as arising from an *anti*-addition to the C=C double bond of **1b**, in analogy to the stereochemical output observed in the case of **4s** and **4t**.
^{1}H NMR (300 MHz, *acetone-d*$_6$): δ 3.32-3.20 (m, 1H), 2.96 (d, J = 6.5 Hz, 1H), 2.61-2.44 (m, 2H), 1.97 (t, J = 7.5 Hz, 2H), 1.46 (d, J = 7.3 Hz, 3H), 1.12 (s, 3H), 1.10 (s, 3H); ^{13}C NMR (75 MHz, *acetone-d*$_6$): δ 174.7, 172.5, 120.8, 56.5, 38.2, 36.6, 35.8, 23.9, 23.4, 17.2, 12.3. IR (*neat*, ν/cm^{-1}): 2975 ,1723, 1278, 820. HRMS (ESI) m/z: [M-H]$^{-}$ calcd. for $C_{11}H_{16}NO_4$ 226.1058, found 226.1057.

2-cyclopentyl-3-methylsuccinic acid (4r)

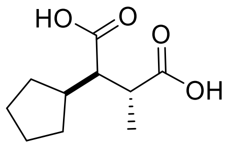

(±)-**4r**

Prepared from citraconic acid **1b** (batch: 2.5 mmol, 325 mg; flow: 1 mmol, 130 mg) and cyclopentane **2r** (5 equiv.; batch: 12.5 mmol, 1.26 mL; flow: 5 mmol, 467 µL, ρ = 0.75 g·mL^{-1}) according to the general procedure (24 h irradiation). The crude mixture was purified through column chromatography (SiO_2; cyclohexane/ethyl acetate 7:3) to afford **4r** (batch: 460 mg, 86% yield, flow: 130 mg, 65% yield) as a white solid (mp: 120-123 °C). The process delivered a single diastereoisomer, that has been assigned as arising from an *anti*-addition to the C=C double bond of **1b**, in analogy to the stereochemical output observed in the case of **4s** and **4t**.
^{1}H NMR (300 MHz, *acetone-d*$_6$): δ 9.81 (bs, 2H), 2.78-2.60 (m, 2H), 2.18-2.02 (m, 1H), 1.89-1.73 (m, 2H), 1.70-1.23 (m, 6H), 1.20 (d, J = 6.4 Hz, 3H); ^{13}C NMR (75 MHz, *acetone-d*$_6$): δ 176.5, 174.7, 53.2, 41.7, 41.7, 31.2, 30.8, 25.6, 25.4, 14.7. IR (*neat*, ν/cm^{-1}): 3009,1704, 1230, 860. HRMS (ESI) m/z: [M-H]$^{-}$ calcd. for $C_{10}H_{15}O_4$ 199.0976, found 199.0966.

2-cyclohexyl-3-methylsuccinic acid (4s)

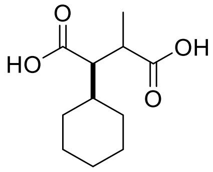

Prepared from citraconic acid **1b** (batch: 2.5 mmol, 325 mg; flow: 1 mmol, 130 mg) and cyclohexane **2s** (5 equiv.; batch: 12.5 mmol, 1.34 mL; flow: 10 mmol, 1.08 mL, ρ = 0.78 g·mL^{-1}) according to the general procedure (24 h irradiation). The crude mixture was purified through column chromatography (SiO_2; cyclohexane/ethyl acetate 7:3) to afford **4s** (*ca*. 1.5:1 mixture of diastereoisomers; batch: 305 mg, 37% yield, flow: 143 mg, 67% yield) as a white solid. The major diastereoisomer has been assigned as arising from an *anti*-addition to the C=C double bond of **1b**, based on the comparison of the chemical shifts of carboxyl groups in the C-NMR spectrum with those of a related succinic acid derivative previously reported in the literature.[S19]

^{1}H NMR (300 MHz, *acetone-d$_6$*): δ 10.59 (bs, 2H), 2.90-2.72 (m, 1H), 2.59-2.48 (m, 1H), 1.86-1.55 (m, 6H), 1.34-0.97 (m, 5H), 1.21 (d, *J* = 6.9 Hz, 3H, only minor isomer), 1.16 (d, *J* = 7.3 Hz, 3H, only major isomer); ^{13}C NMR (75 MHz, *acetone-d$_6$*): δ 177.2, 176.6, 175.2, 174.4, 54.6, 53.9, 39.1, 39.3, 37.4, 32.9, 32.3, 27.5, 27.3, 27.2, 27.2, 27.1, 15.7, 15.4. IR (*neat*, ν/cm^{-1}): 3009, 1704, 1230, 860. HRMS (ESI) m/z: [M-H]$^-$ calcd. for $C_{11}H_{17}O_4$ 213.1132, found 213.1122.

2-cycloheptyl-3-methylsuccinic acid (4t)

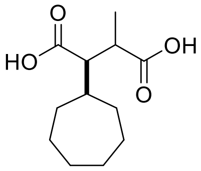

Prepared from citraconic acid **1b** (batch: 2.5 mmol, 325 mg) and cycloheptane **2t** (10 equiv.; batch: 25 mmol, 3.02 mL, ρ = 0.81 g·mL^{-1}) according to the general procedure (40 h irradiation), while adopting a MeCN/CH_2Cl_2 5:1 mixture as reaction medium. The crude mixture was purified through column chromatography (SiO_2; cyclohexane/ethyl acetate 7:3) to afford **4t** (*ca*. 4.5:1 mixture of diastereoisomers; batch: 262 mg, 46% yield) as a white solid. The major diastereoisomer has been assigned as arising from an *anti*-addition to the C=C double bond of **1b**, based on the comparison of the chemical shifts of carboxyl groups in the C-NMR spectrum with those of a related succinic acid derivative previously reported in the literature.[S19]

^{1}H NMR (300 MHz, *acetone-d$_6$*): δ 10.45 (bs, 2H), 2.86-2.72 (m, 1H), 2.64-2.53 (m, 1H), 1.90-1.28 (m, 13H), 1.23 (d, *J* = 7.2 Hz, 3H, only minor isomer), 1.18 (d, *J* = 6.8 Hz, 3H, only major isomer); ^{13}C NMR (75 MHz, *acetone-d$_6$*): 177.7, 176.9, 175.8, 174.8, 55.5, 54.7, 41.2, 40.8, 40.2, 39.2, 35.0, 34.6, 30.7, 29.4, 29.2, 28.9, 28.8, 28.3, 28.0, 27.9, 27.8, 16.3, 16.0. IR (*neat*, ν/cm^{-1}): 3012, 1745, 1279, 832. HRMS (ESI) m/z: [M-H]$^-$ calcd. for $C_{12}H_{19}O_4$ 227.1289, found 227.1284.

2-cyclooctyl-3-methylsuccinic acid (4u)

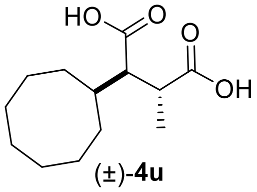

(±)-**4u**

Prepared from citraconic acid **1b** (batch: 2.5 mmol, 325 mg; flow: 0.9 mmol, 117 mg) and cyclooctane **2u** (10 equiv.; batch: 25 mmol, 3.33 mL, ρ = 0.83 g·mL^{-1}) according to the general procedure, (40 h irradiation) while adopting a MeCN/CH_2Cl_2 5:1 mixture as reaction medium. The crude mixture was purified through column chromatography (SiO_2; cyclohexane/ethyl acetate 7:3) to afford **4u** (batch: 211 mg, 58% yield) as a white solid (mp: 130-133 °C). The process delivered a single diastereoisomer, that has been assigned as arising from an *anti*-addition to the C=C double bond of **1b**, in analogy to the stereochemical output observed in the case of **4s** and **4t**.

^{1}H NMR (300 MHz, *acetone-d*$_6$): 2.88-2.75 (m, 1H), 2.53 (dd, *J* = 9.3, 4.8 Hz, 1H), 2.04-1.94 (m, 1H), 1.81-1.26 (m, 14H), 1,23 (d, *J* = 7.2 Hz, 3H); ^{13}C NMR (75 MHz, *acetone-d*$_6$): 177.3, 175.5, 54.9, 39.7, 36.4, 33.4, 29.0, 27.4, 27.3, 27.0, 27.0, 26.3, 15.7. IR (*neat*, ν/cm^{-1}): 2996, 1723, 1306, 825. HRMS (ESI) m/z: [M-H]$^-$ calcd. for $C_{13}H_{21}O_4$ 241.1445, found 241.1436.

4. References

[S1] S. Protti, D. Ravelli, M. Fagnoni, A. Albini, *Chem. Commun.* **2009**, 7351–7353.

[S2] A. J. N. Price, A. J. Capel, R. J. Lee, P. Pradel, S. D. R. Christie, *J. Flow Chem.* **2021**, *11*, 37–51.

[S3] Z. Xu, L. Fu, X. Fang, B. Huang, L. Zhou, J.-P. Wan, *Org. Lett.* **2021**, *23*, 5049–5053.

[S4] T. Lin, J. Mi, L. Song, J. Gan, P. Luo, J. Mao, P. J. Walsh, *Org. Lett.* **2018**, *20*, 1191–1194.

[S5] L. Z. Fekri, M. Nikpassand, *Russ. J. Gen. Chem.* **2014**, *84*, 1825–1829.

[S6] Y. Hua, H. Bin, T. Wei, H. Cheng, Z. Lin, X. Fu, Y.-Q. Li, J.-H. Xie, P.-C. Yan, Q.-L. Zhou, *Org. Lett.* **2020**, *22*, 818–822.

[S7] A. Karmakar, R. Y. Nimje, A. Silamkoti, M. Pitchai, M. Basha, C. Singarayer, D. Ramasamy, G. T. V. Babu, R. Samikannu, S. Subramaniam, et al., *Org. Process Res. Dev.* **2021**, *25*, 1001–1014.

[S8] N. Sakai, S. Horikawa, Y. Ogiwara, *RSC Adv.* **2016**, *6*, 81763–81766.

[S9] B. Sharma, A. Verma, U. K. Sharma, S. Prajapati, *Med. Chem. Res.* **2014**, *23*, 146–157.

[S10] K. Zheng, G. Xiao, T. Guo, Y. Ding, C. Wang, T.-P. Loh, X. Wu, *Org. Lett.* **2020**, *22*, 694–699.

[S11] S. Natori, Y. Kumada, *Chem. Pharm. Bull.* **1965**, *13*, 1472–1475.

[S12] S. Li, J. Zhang, H. Li, L. Feng, P. Jiao, *J. Org. Chem.* **2019**, *84*, 9460–9473.

[S13] A. Takeda, S. Wada, M. Fujii, I. Nakasima, S. Hirata, *Bull. Chem. Soc. Jpn.* **1971**, *44*, 1342–1345.

[S14] M. D'Ambrosio, A. Guerriero, *J. Chem. Res.* **2002**, *2002*, 631–633.

[S15] P. G. Gassman, K. T. Mansfield, *J. Am. Chem. Soc.* **1968**, *90*, 1517–1524.

[S16] S. C. Bergmeier, K. A. Ismail, *Synthesis* **2000**, 1369–1371.

[S17] K. Alder, H. Söll, H. Söll, *Justus Liebigs Ann. Chem.* **1949**, *565*, 73–99.

[S18] X. Zhang, Y. Gao, R. D. Laishram, K. Li, Y. Yang, Y. Zhan, Y. Luo, B. Fan, *Org. Biomol. Chem.* **2019**, *17*, 2174–2181.

[S19] L. Ernst, W. Trowitzsch, *Chem. Ber.* **1974**, *107*, 3771–3779.

5. Copy of ^{1}H and ^{13}C NMR spectra

4-oxo-4-phenylbutanoic acid (3a)

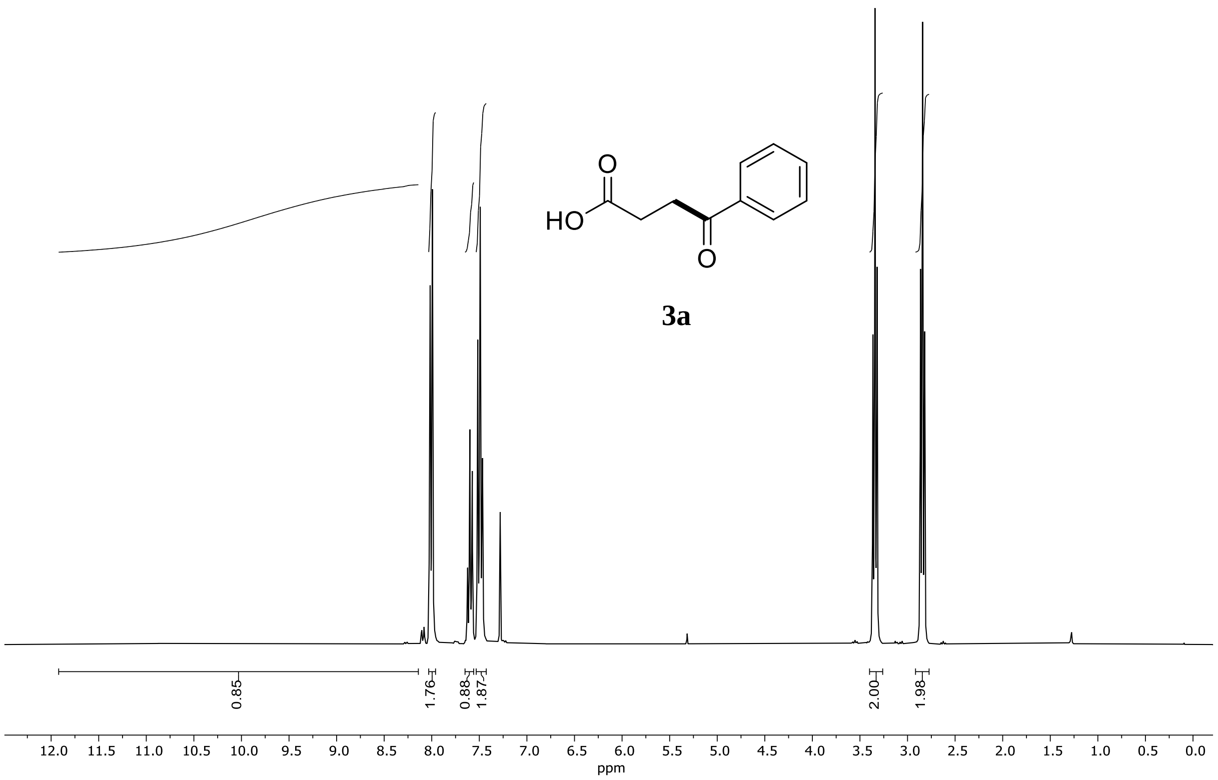

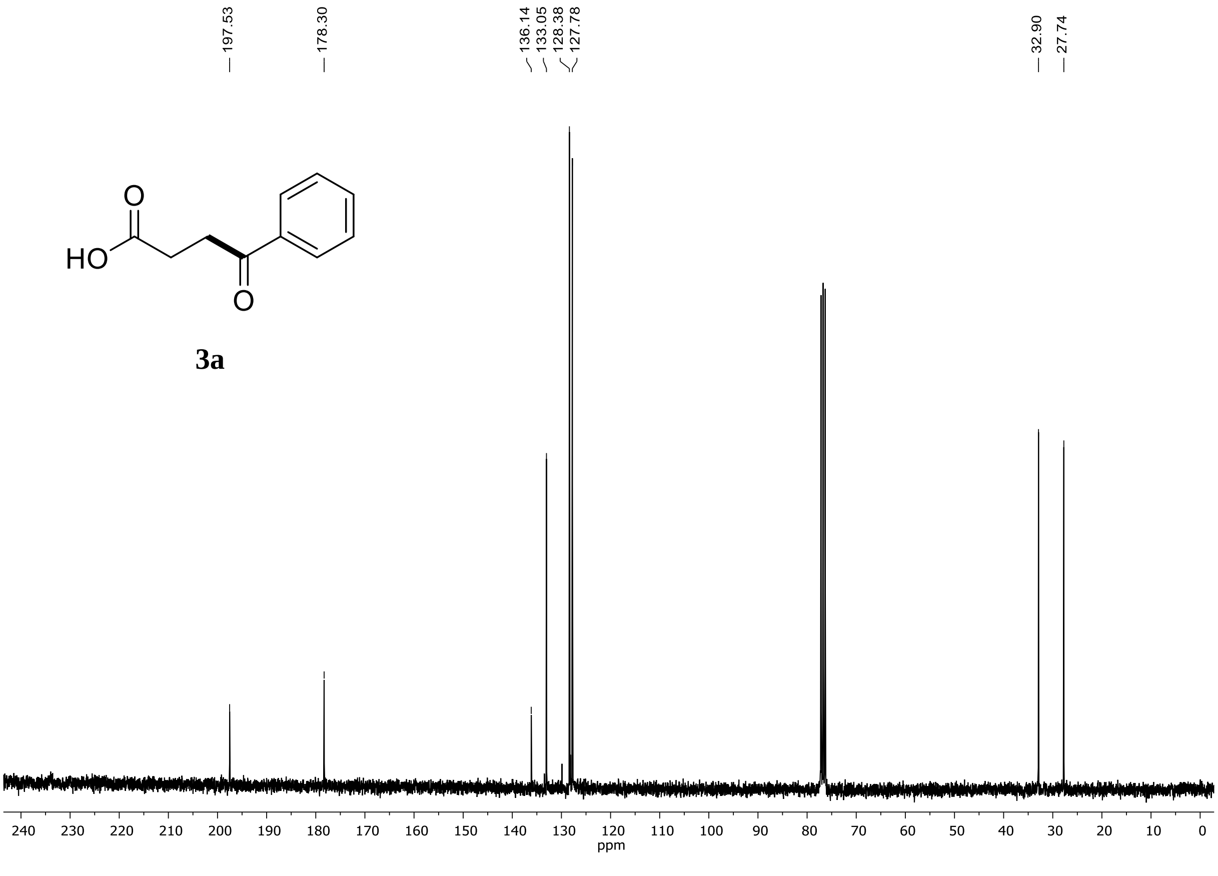

4-(4-methoxyphenyl)-4-oxobutanoic acid (3b)

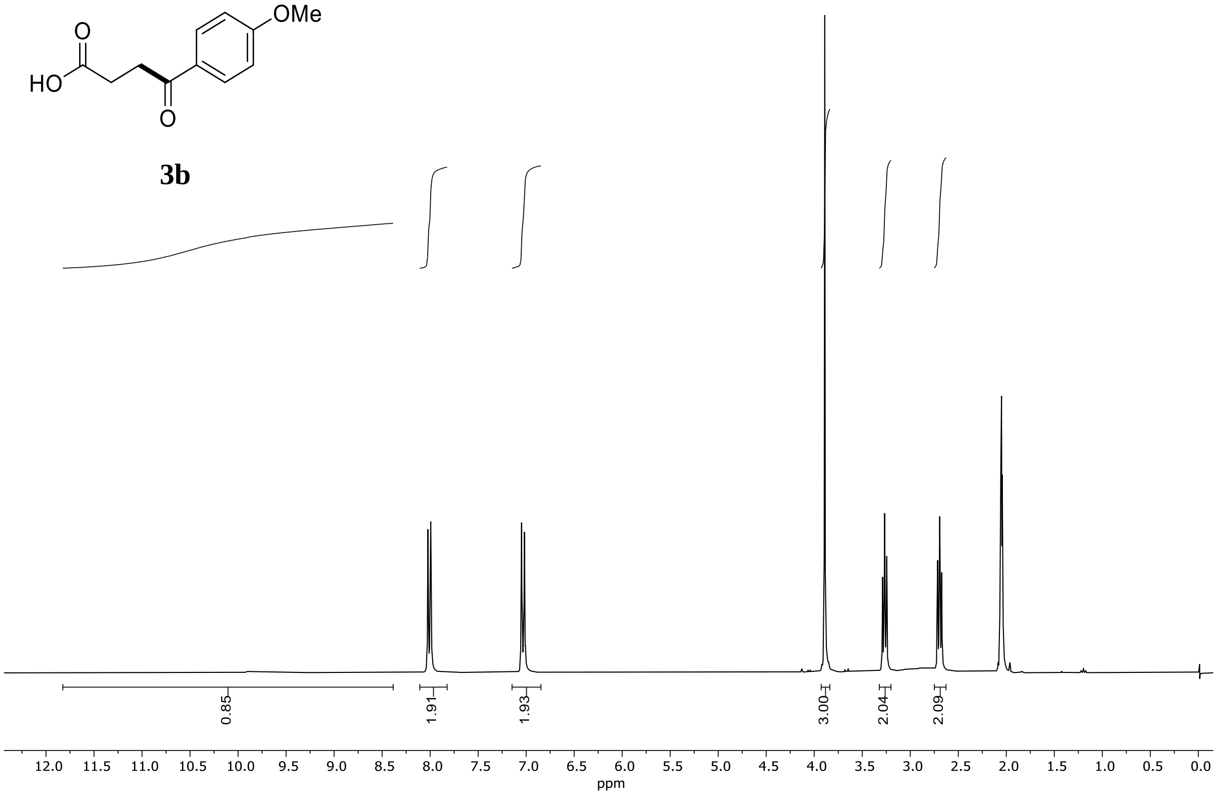

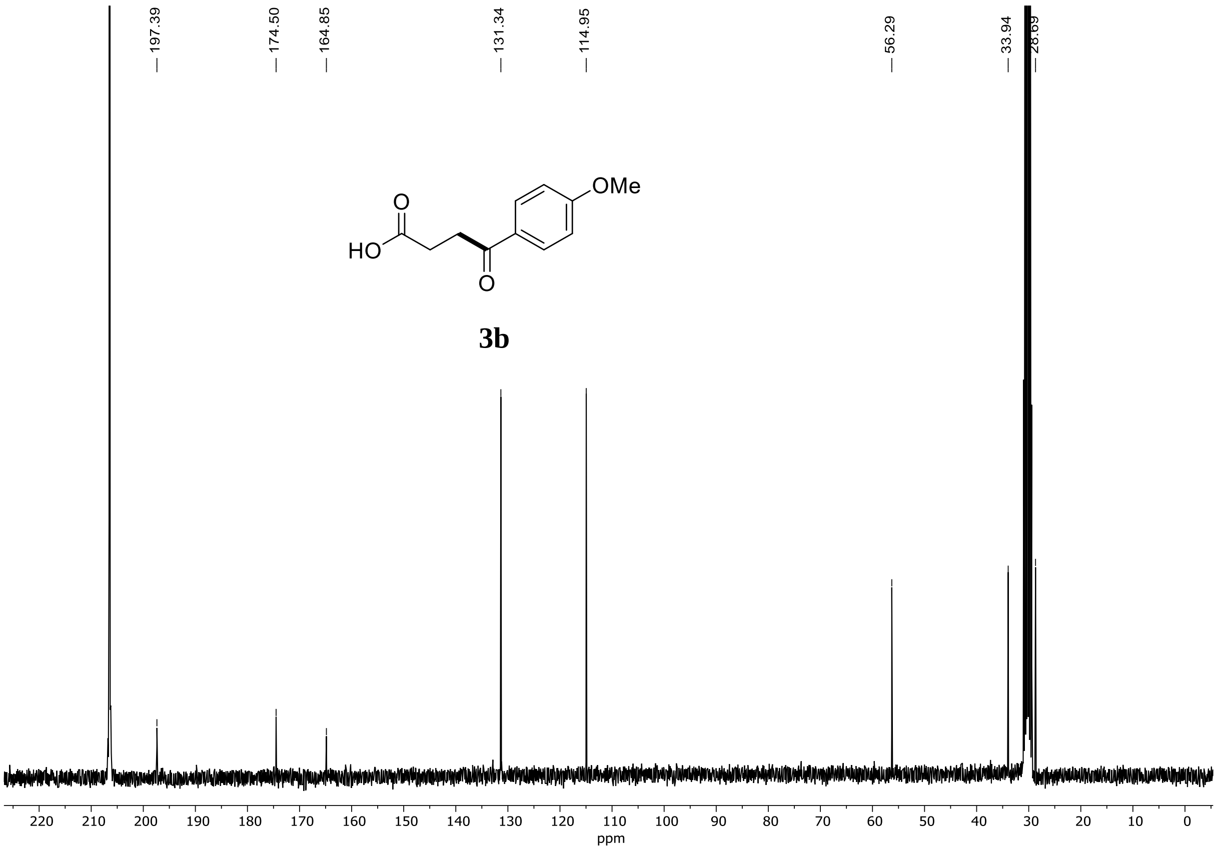

4-(4-chlorophenyl)-4-oxobutanoic acid (3c)

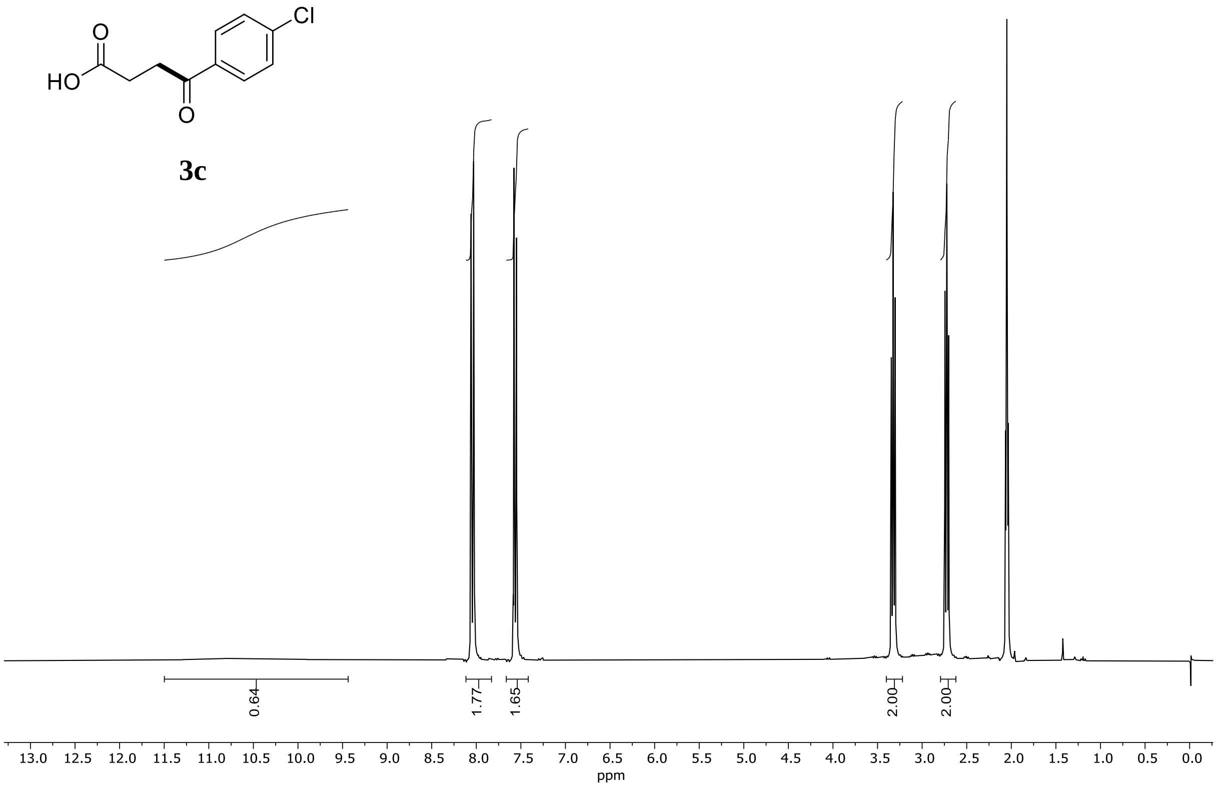

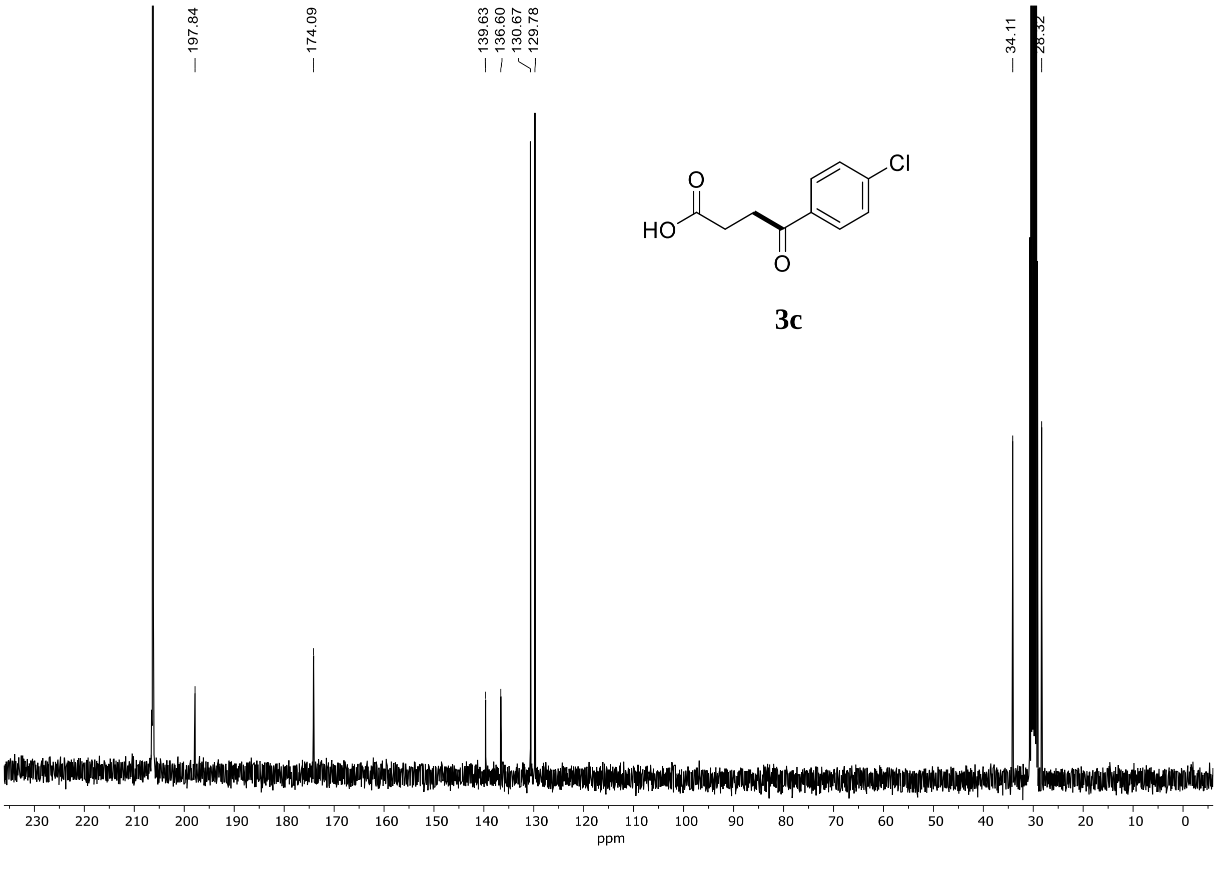

4-(4-bromophenyl)-4-oxobutanoic acid (3d)

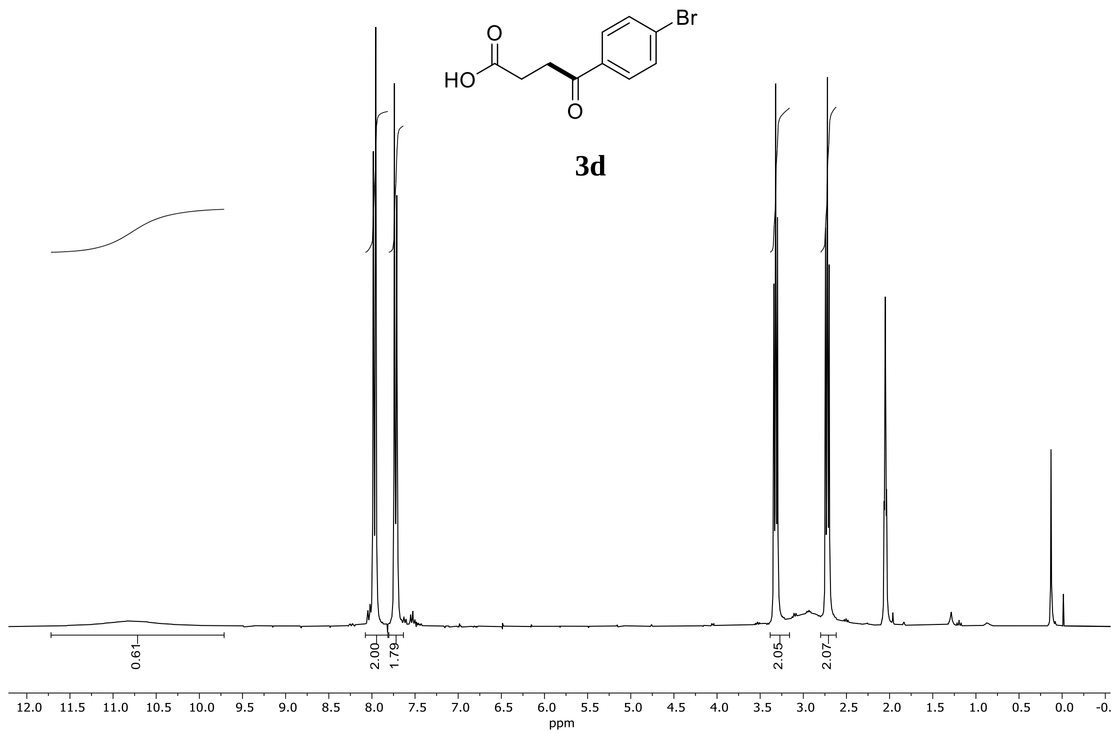

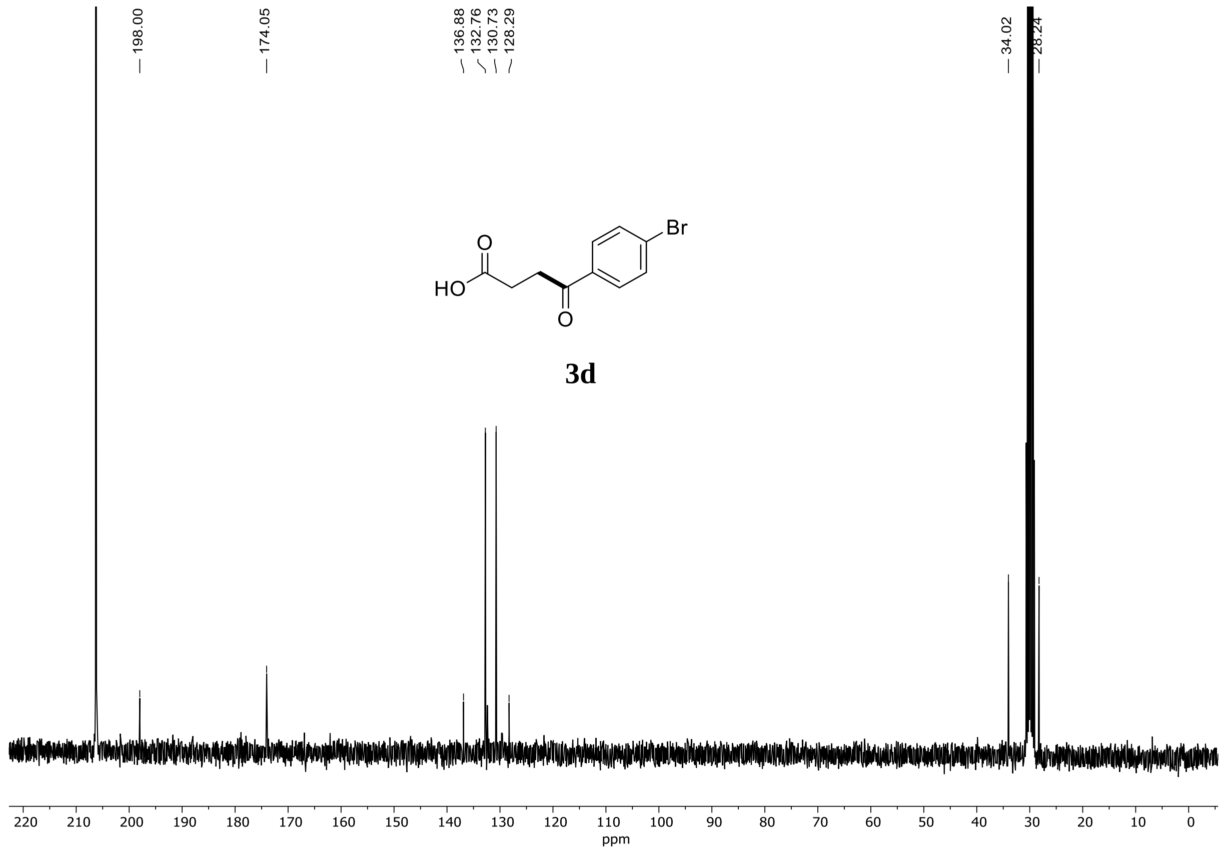

4-(4-fluorophenyl)-4-oxobutanoic acid (3e)

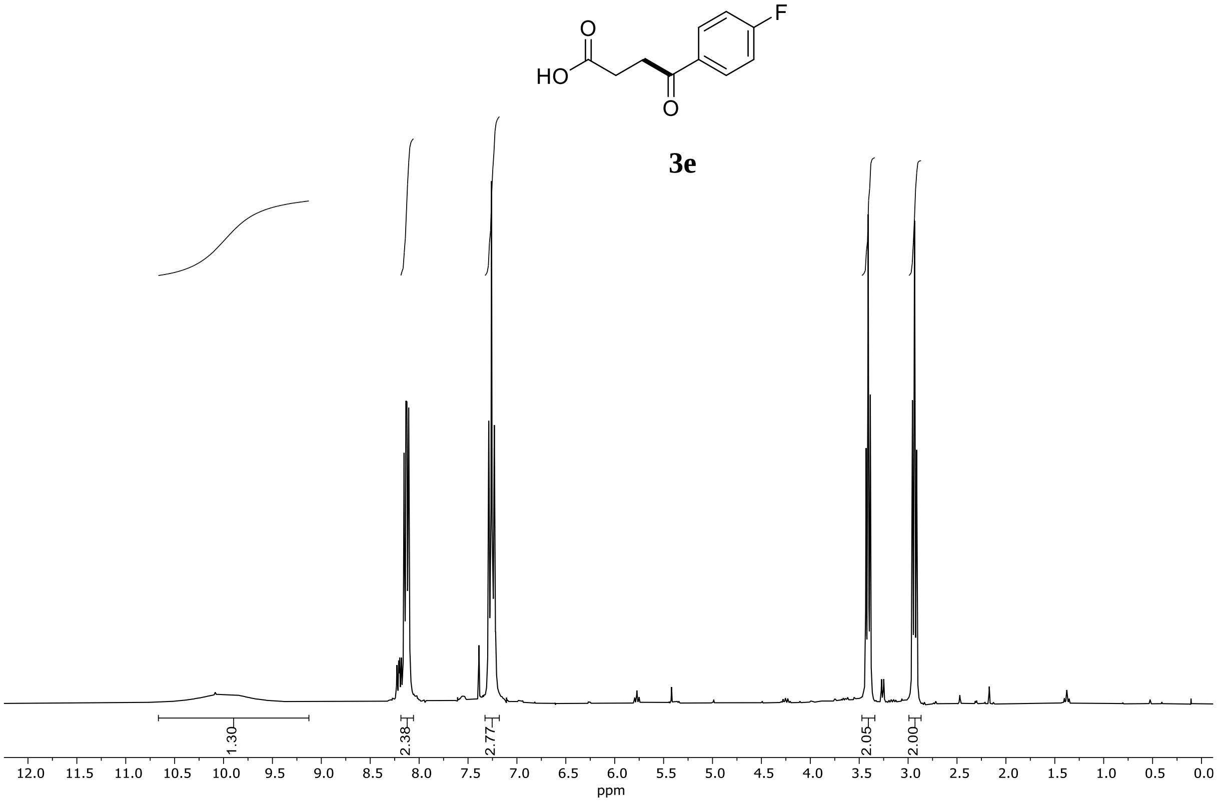

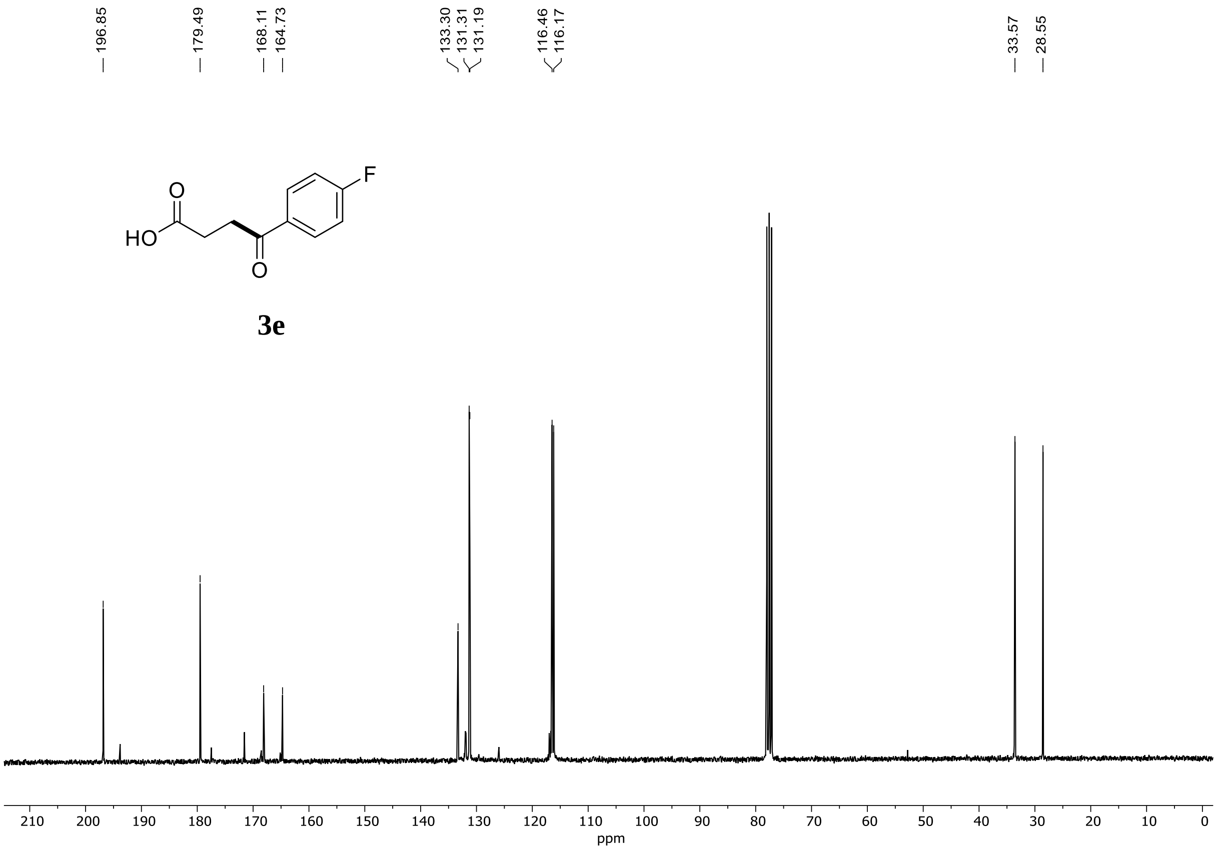

4-oxo-4-(4-(trifluoromethyl)phenyl)butanoic acid (3f)

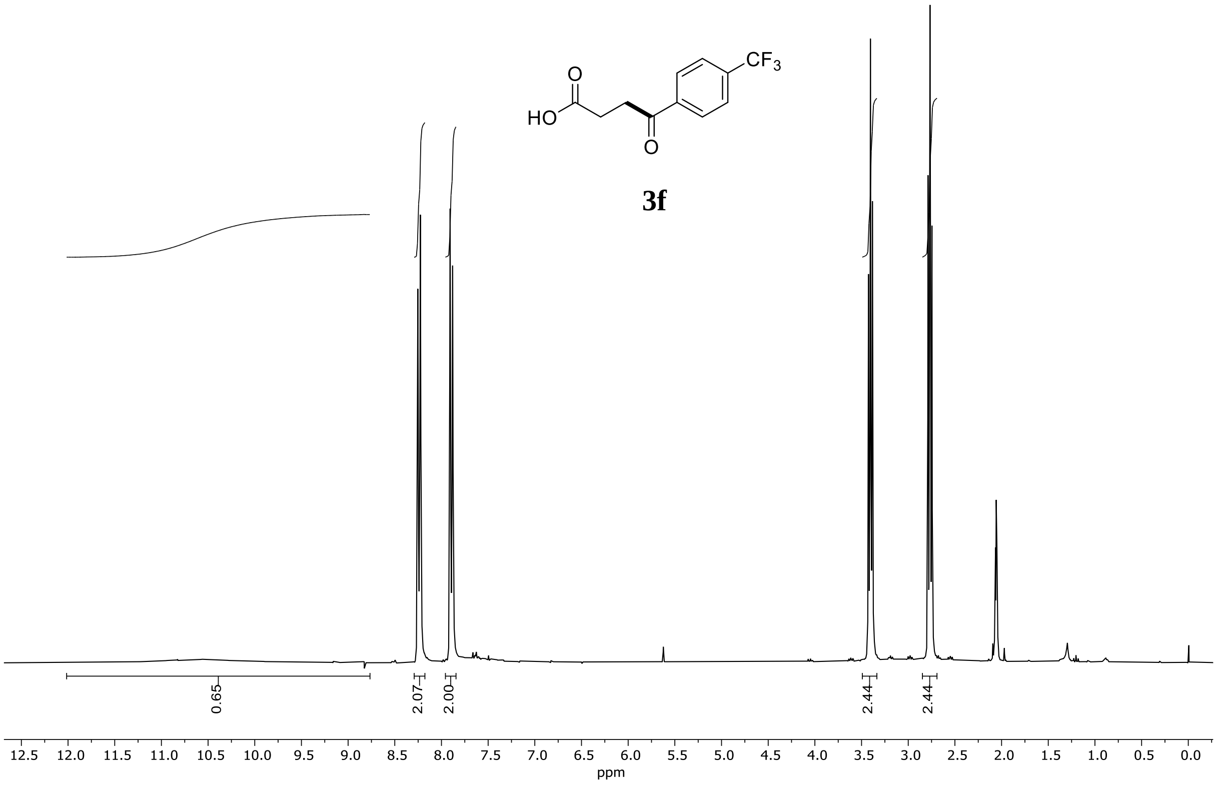

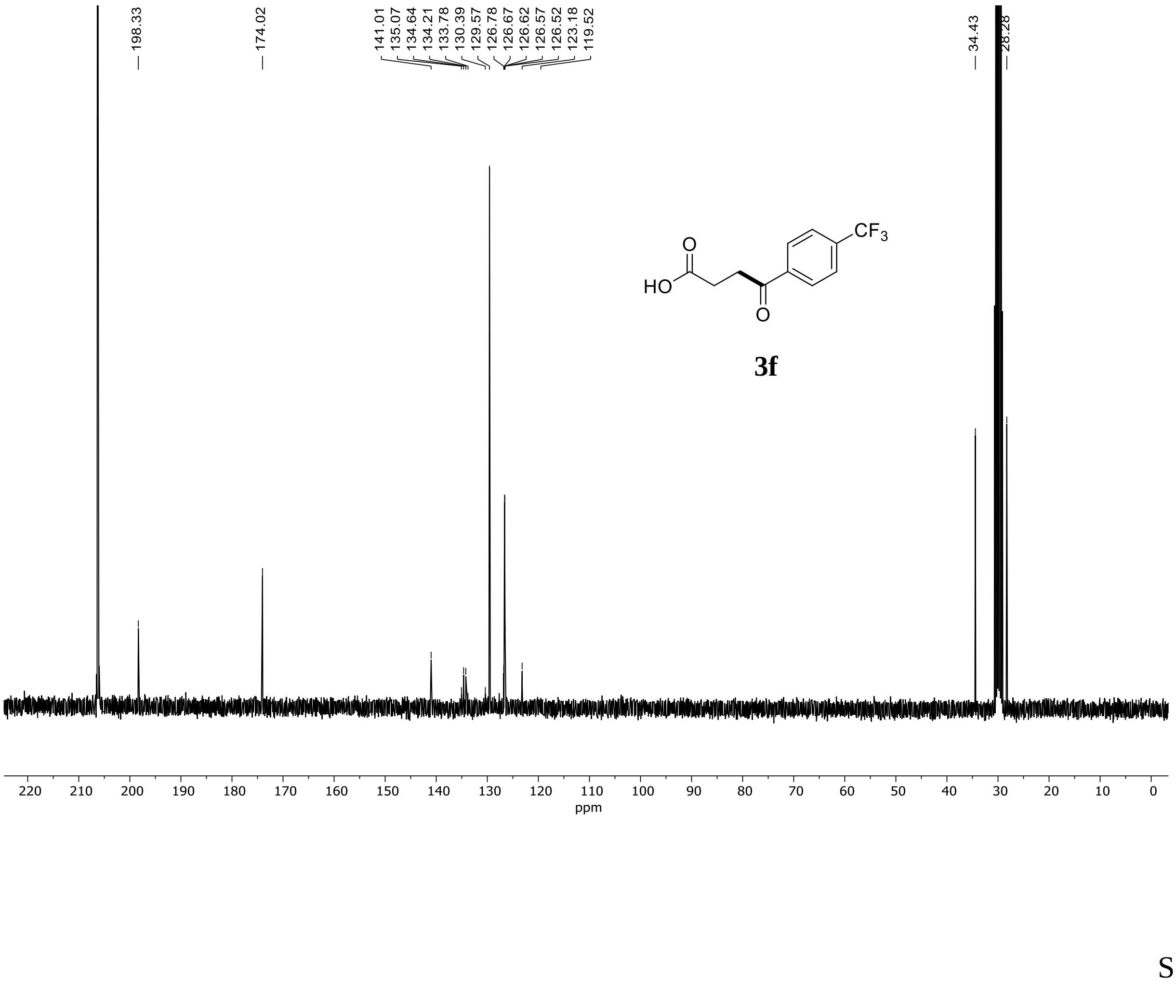

4-oxo-4-(4-tolyl)butanoic acid (3g)

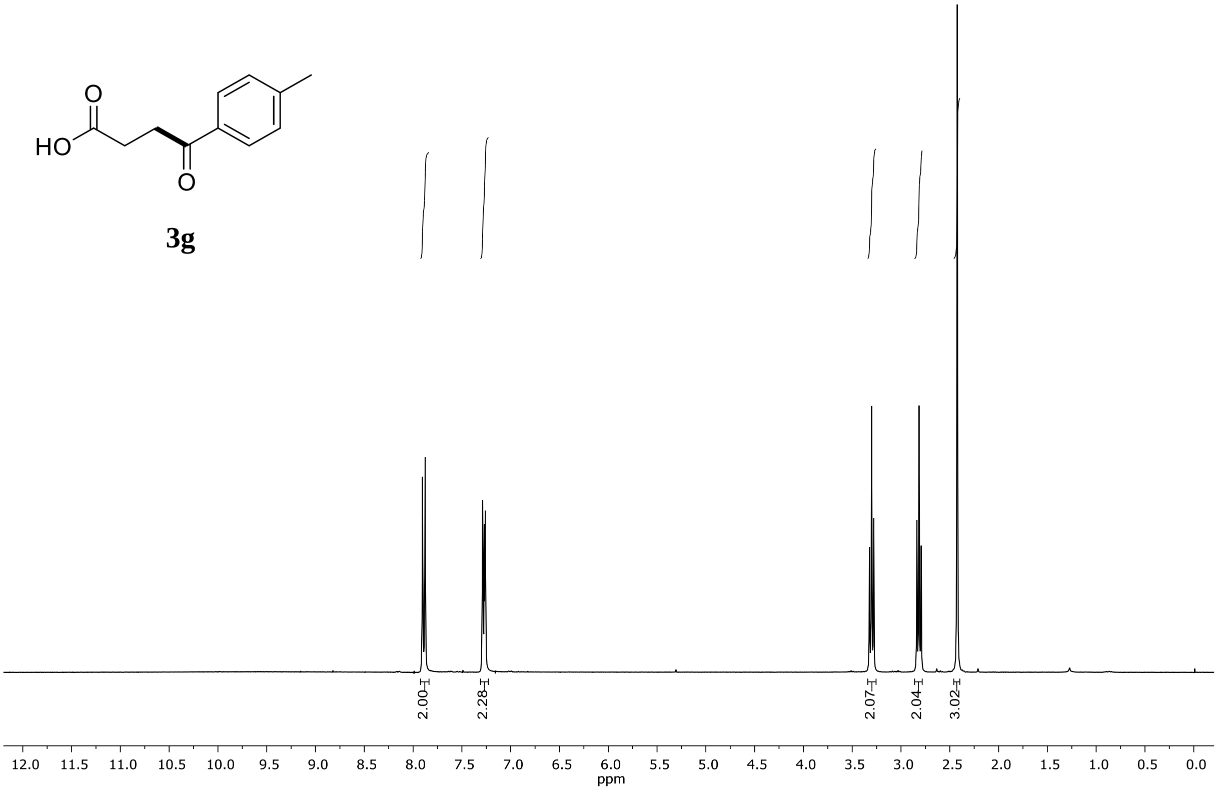

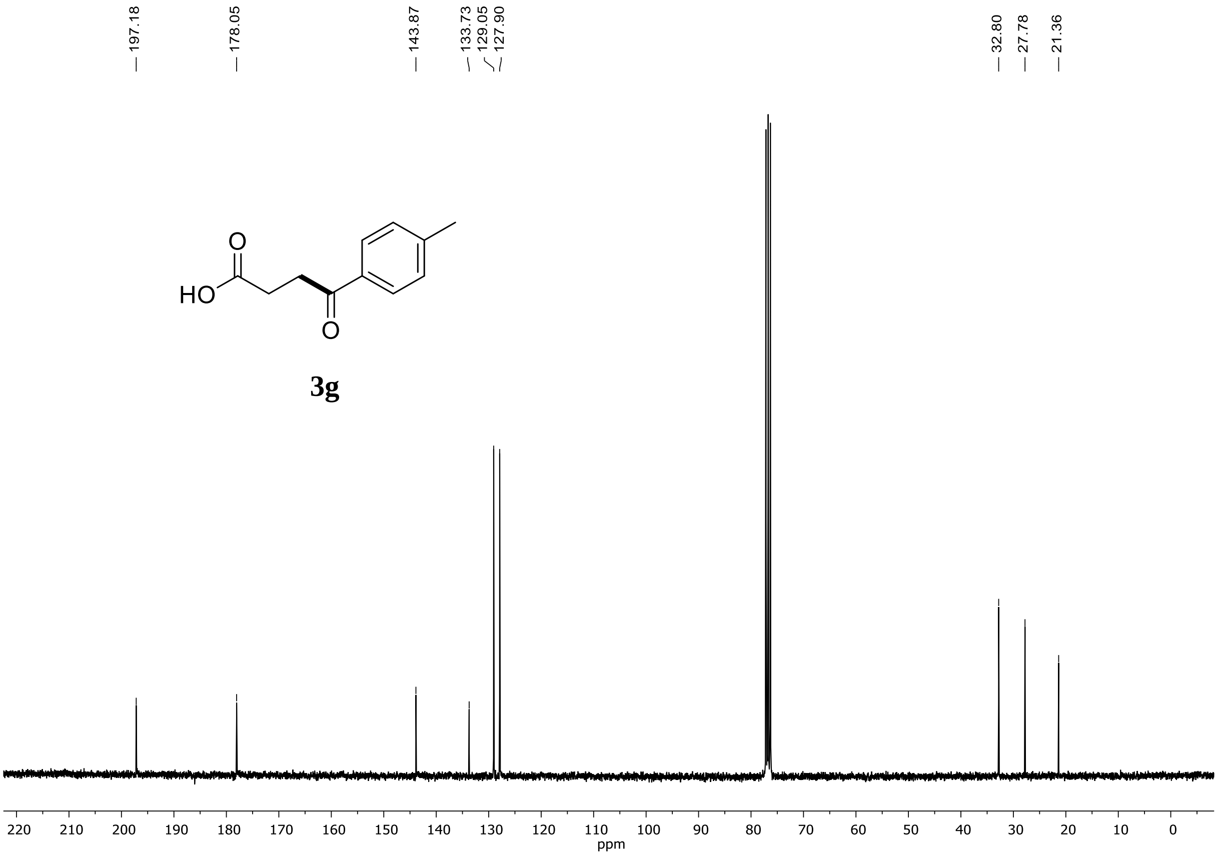

4-(4-ethylphenyl)-4-oxobutanoic acid (3h)

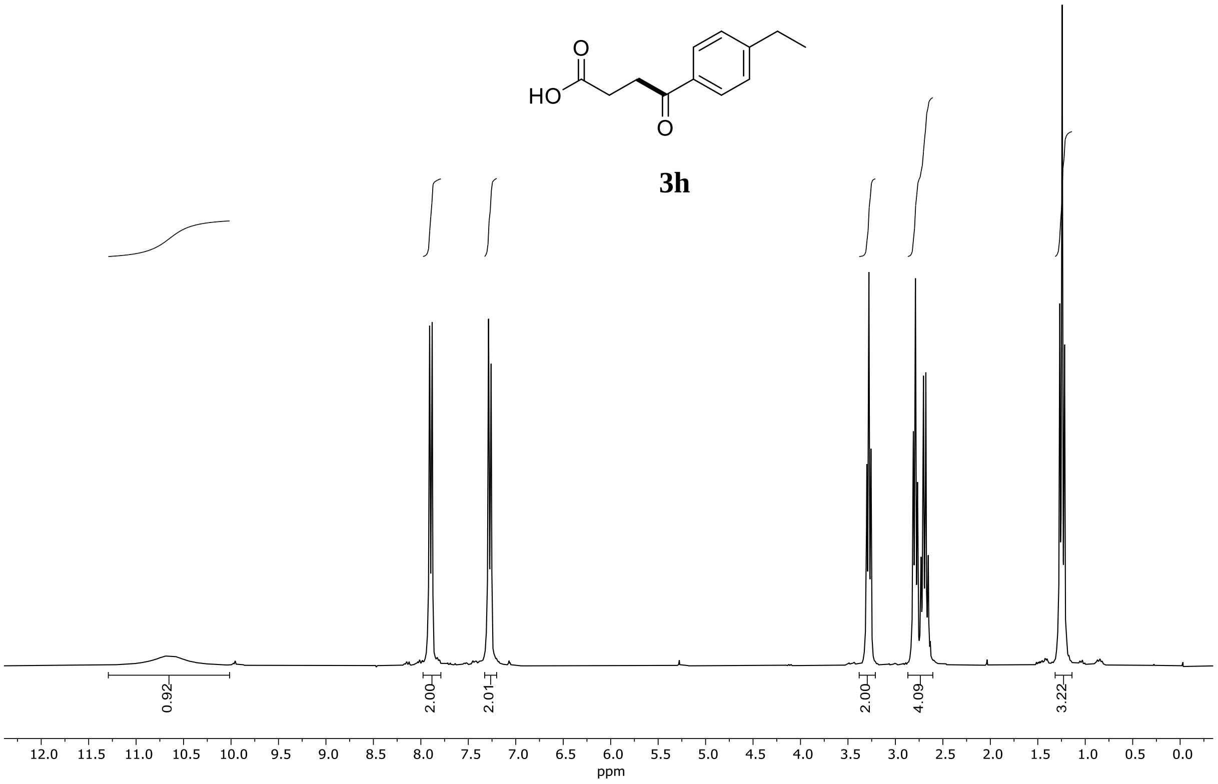

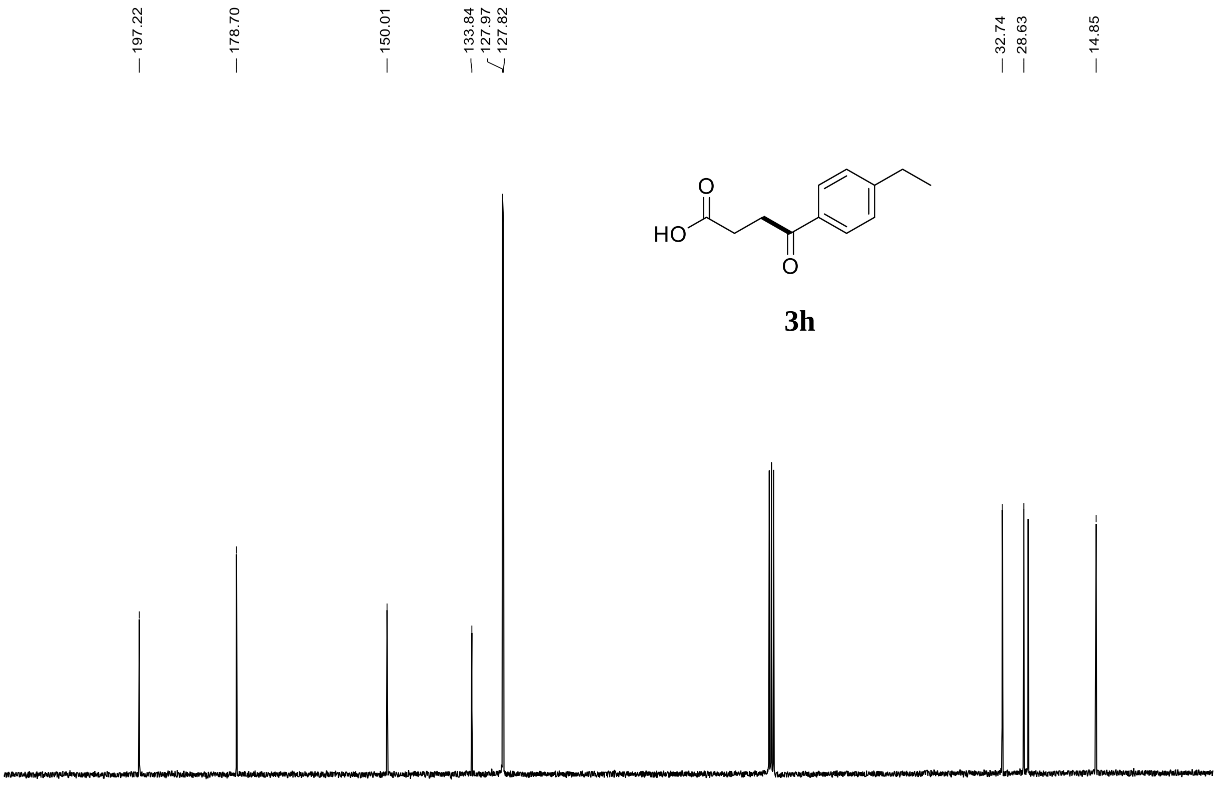

4-([1,1'-biphenyl]-4-yl)-4-oxobutanoic acid (3i)

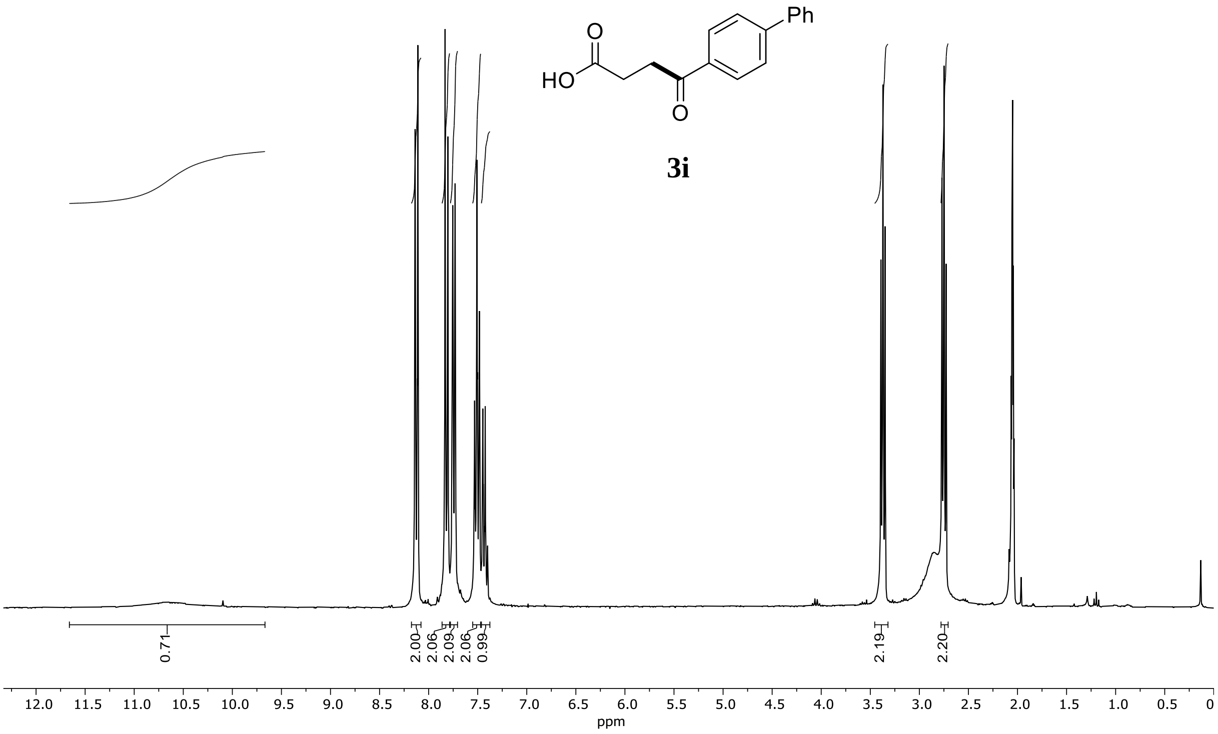

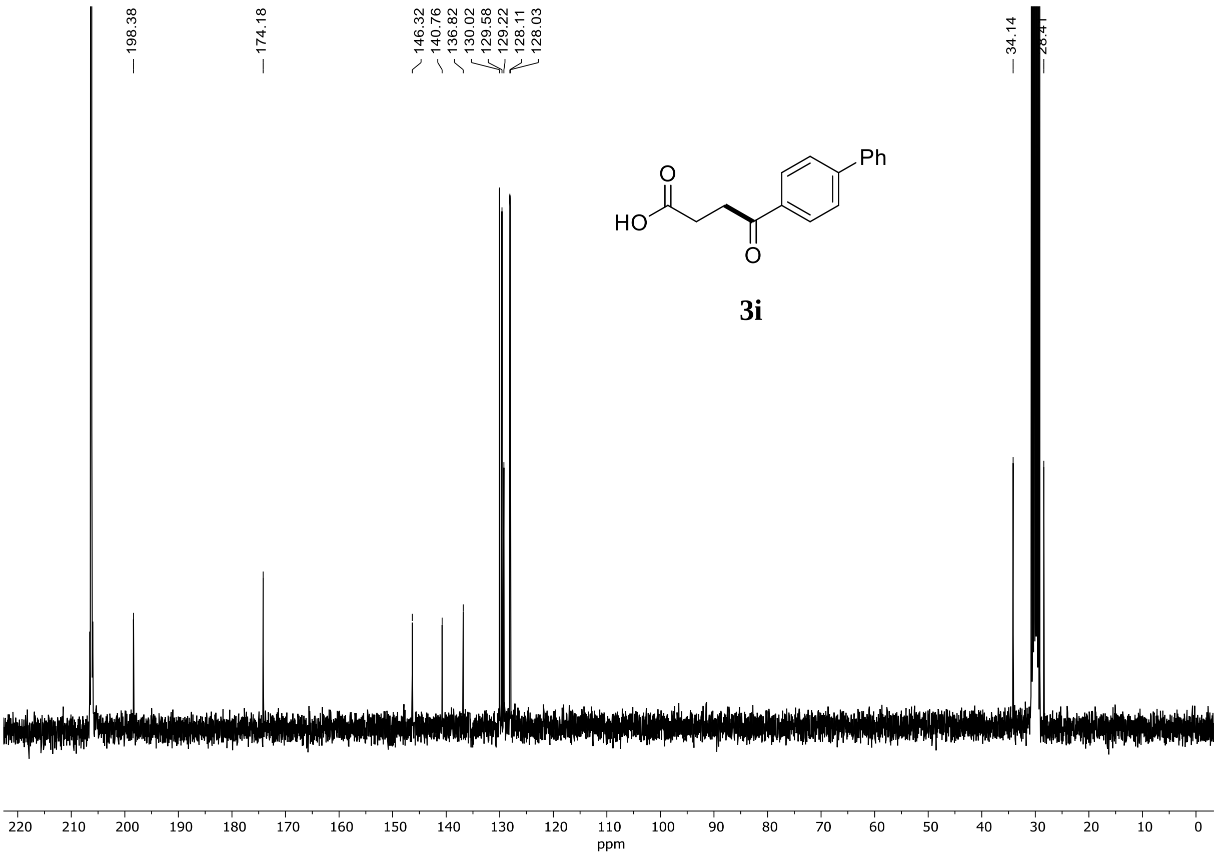

4-oxo-4-(3,4,5-trimethoxyphenyl)butanoic acid (3j)

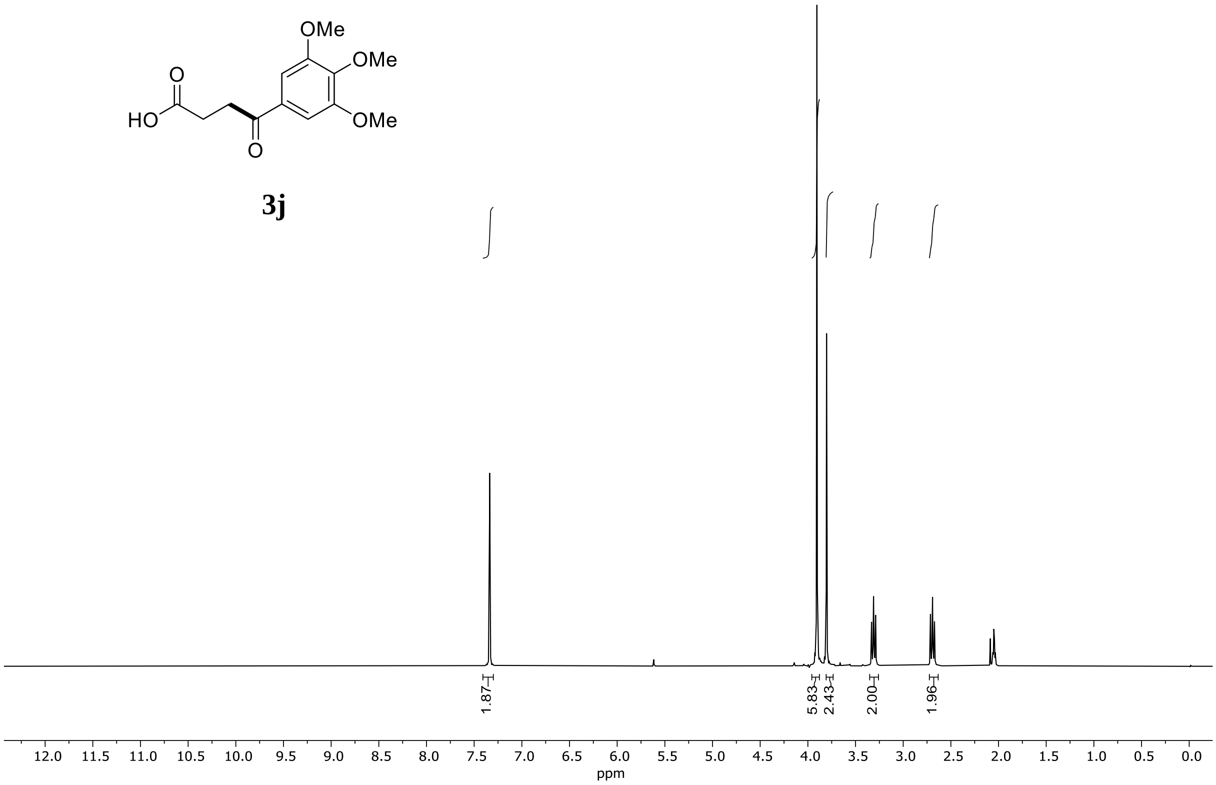

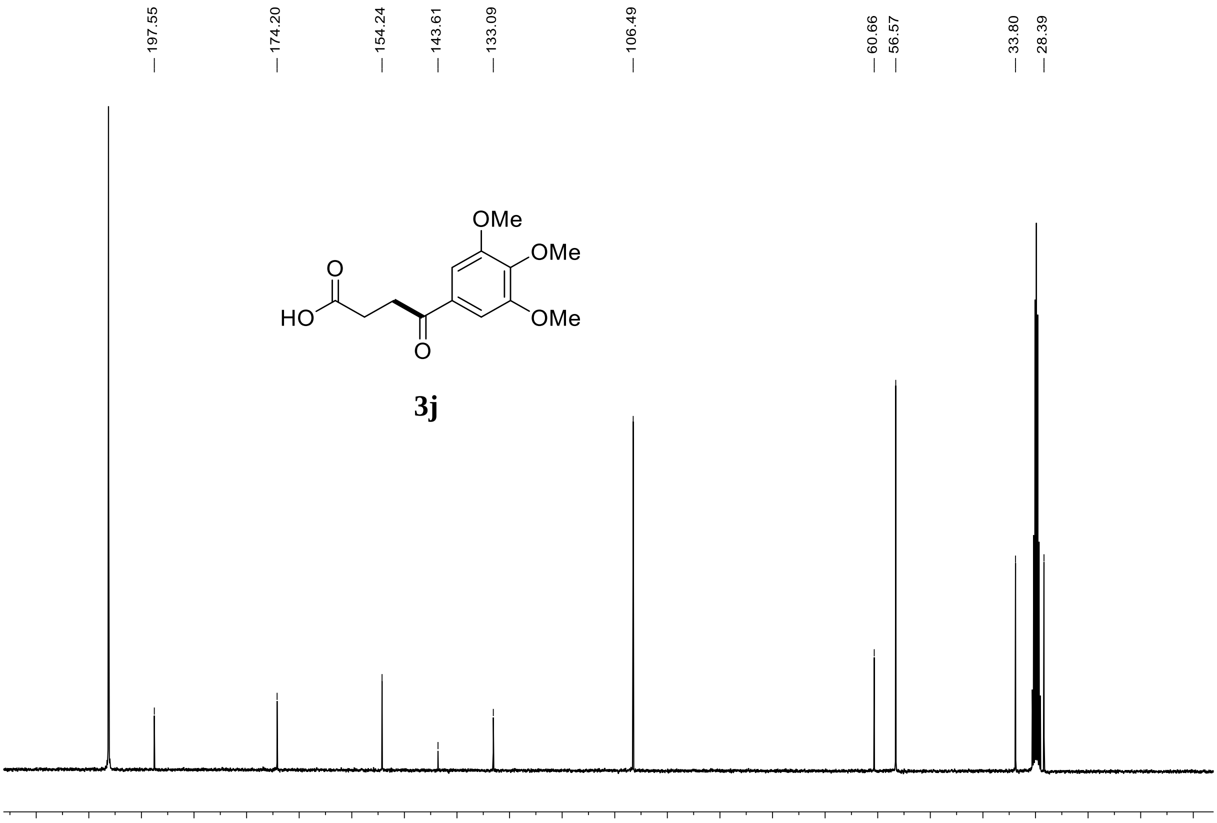

4-oxo-6-phenylhexanoic acid (3k)

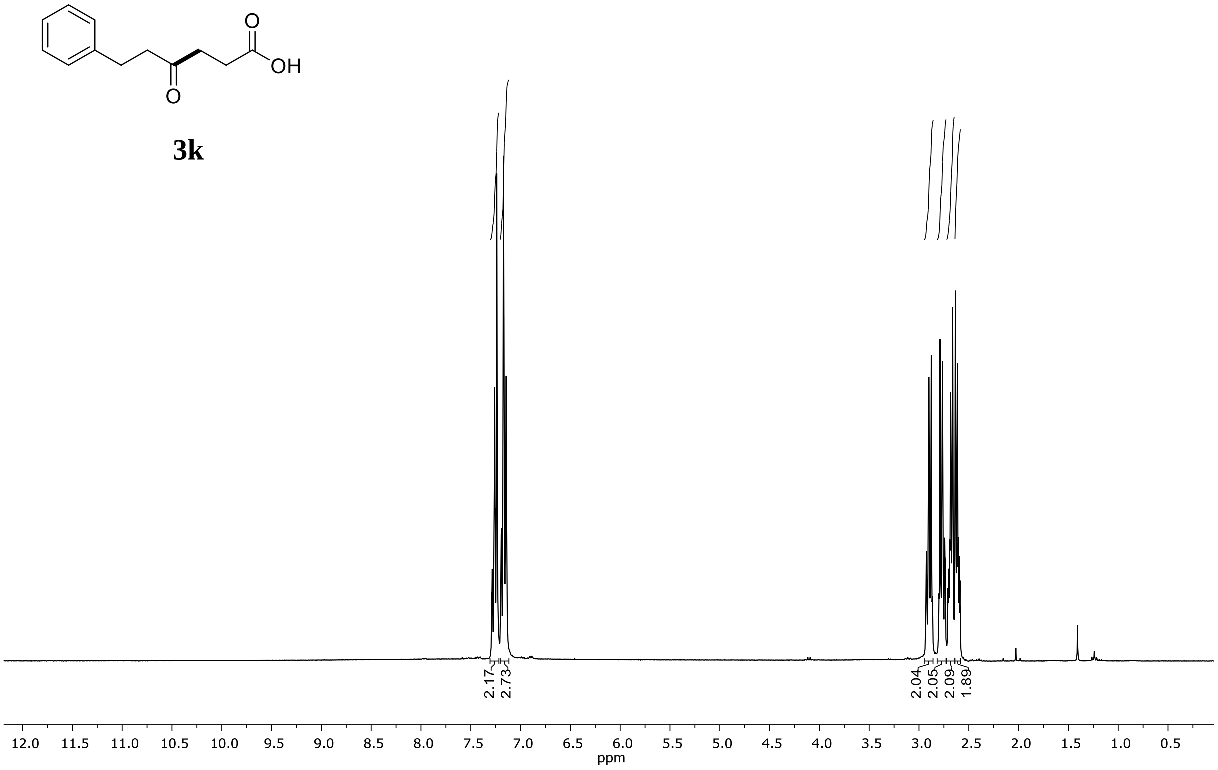

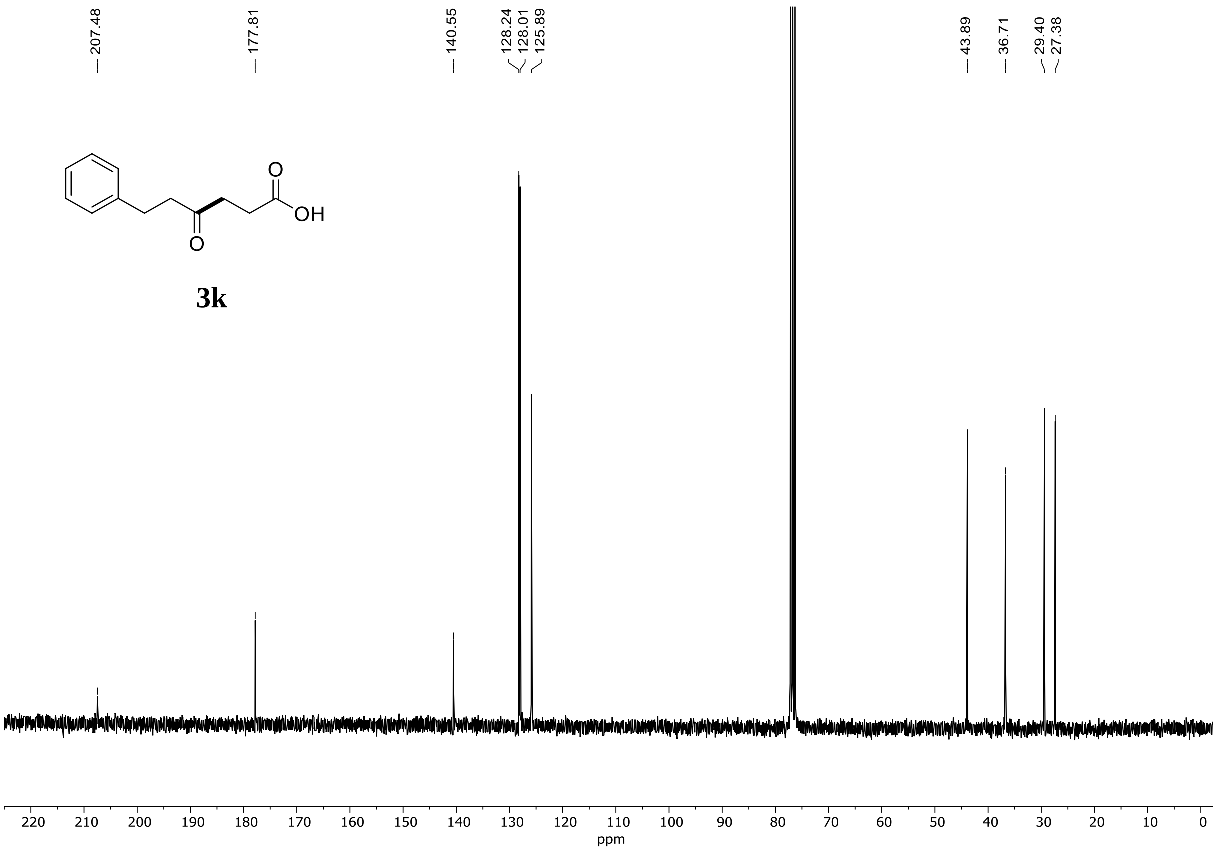

4-oxoundecanoic acid (3l)

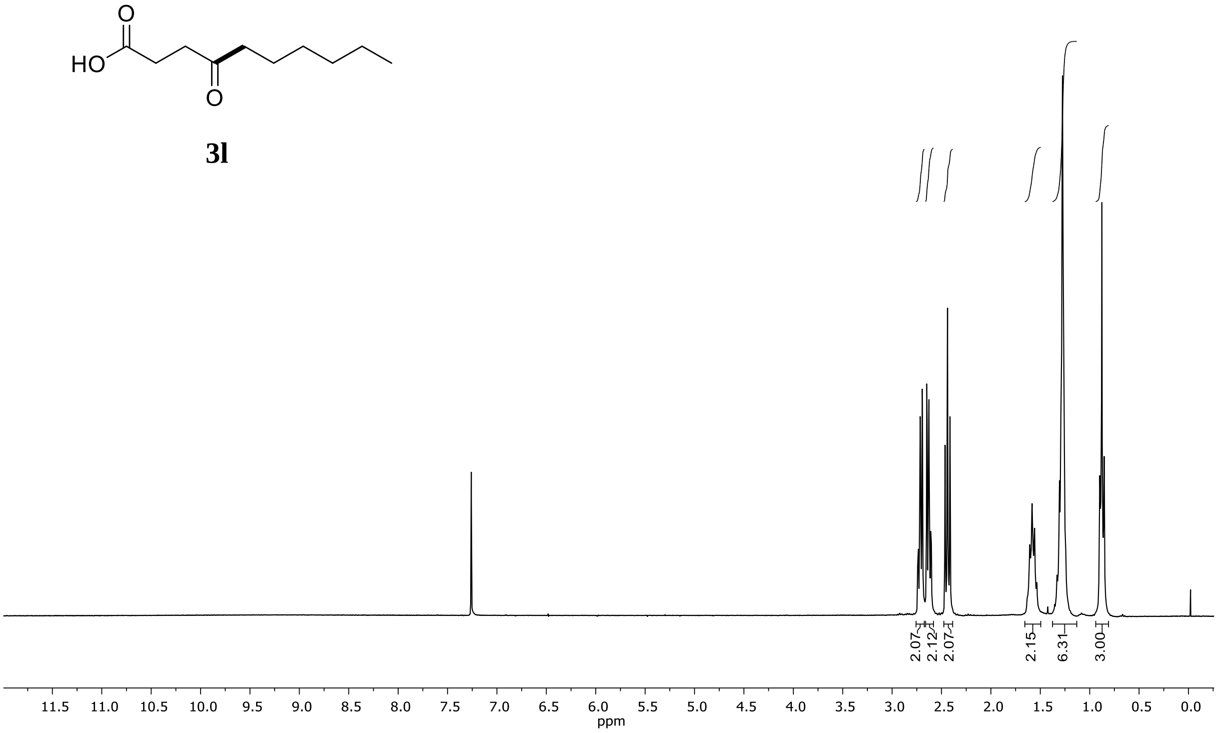

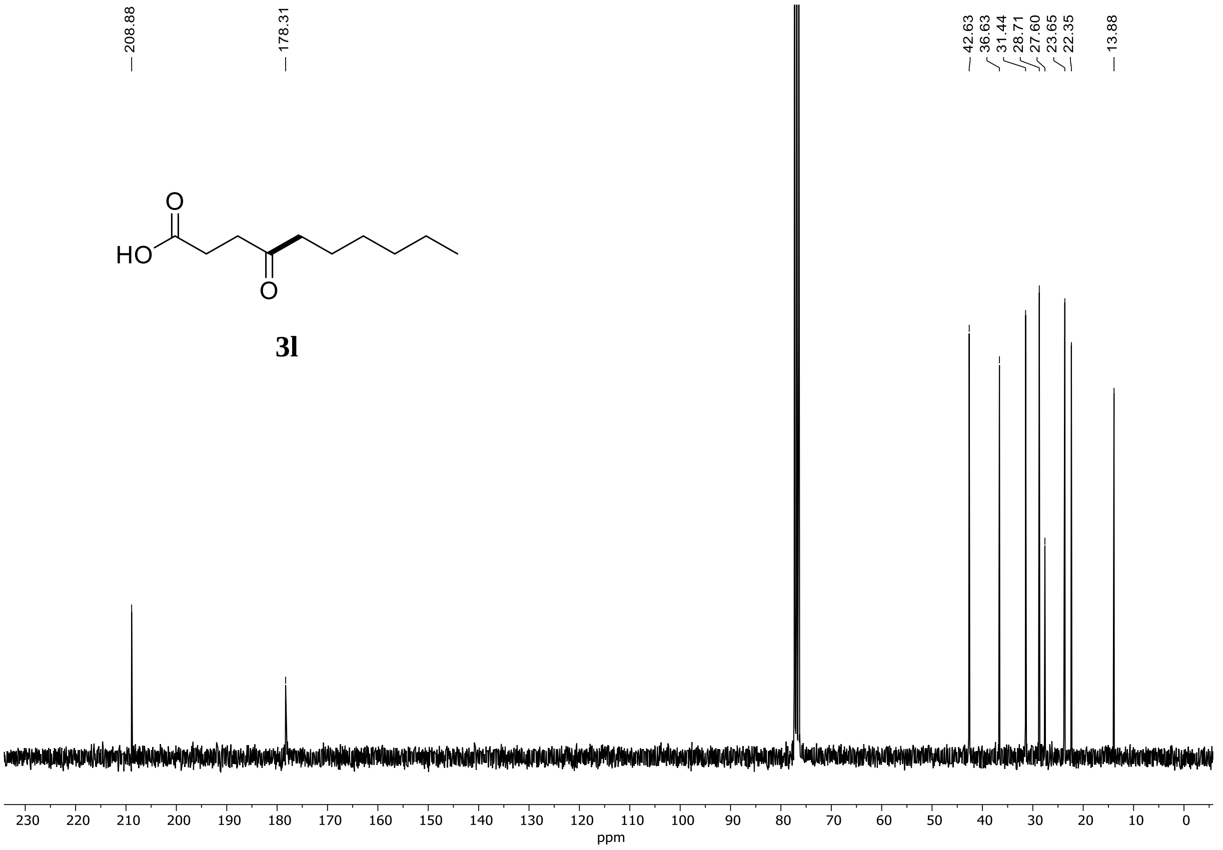

4-oxoundecanoic acid (3m)

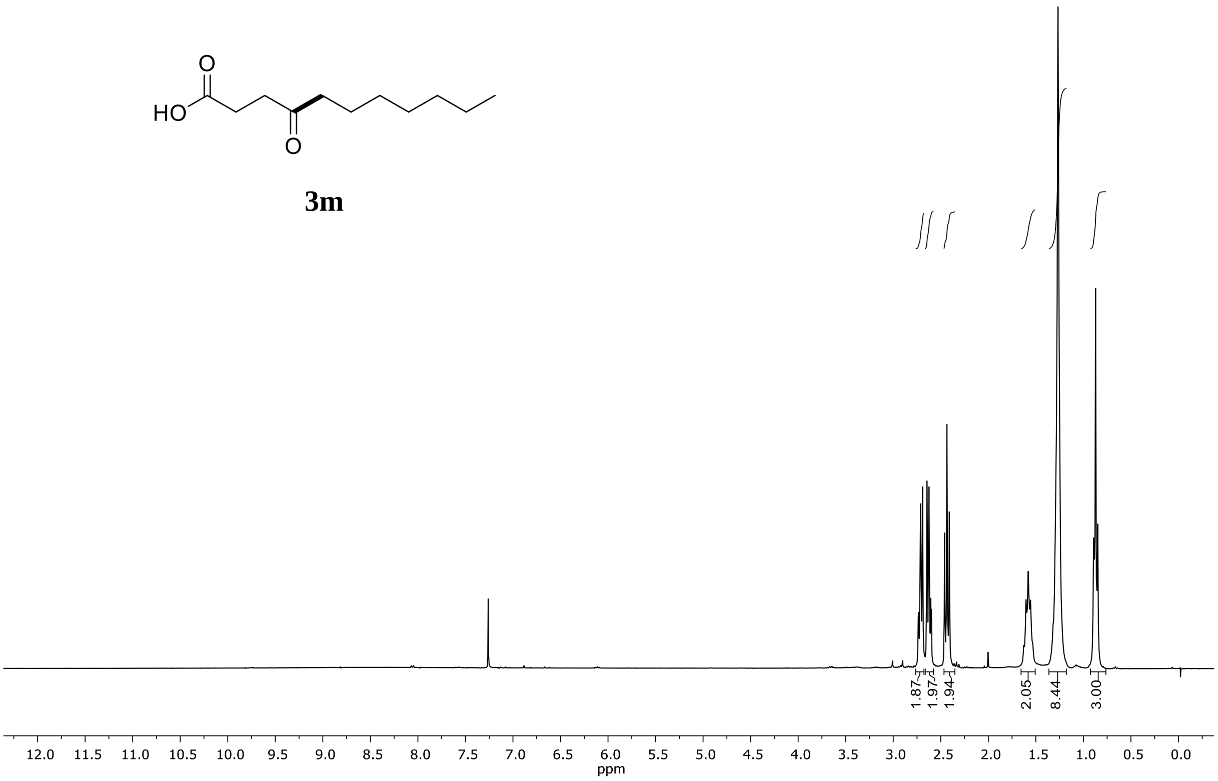

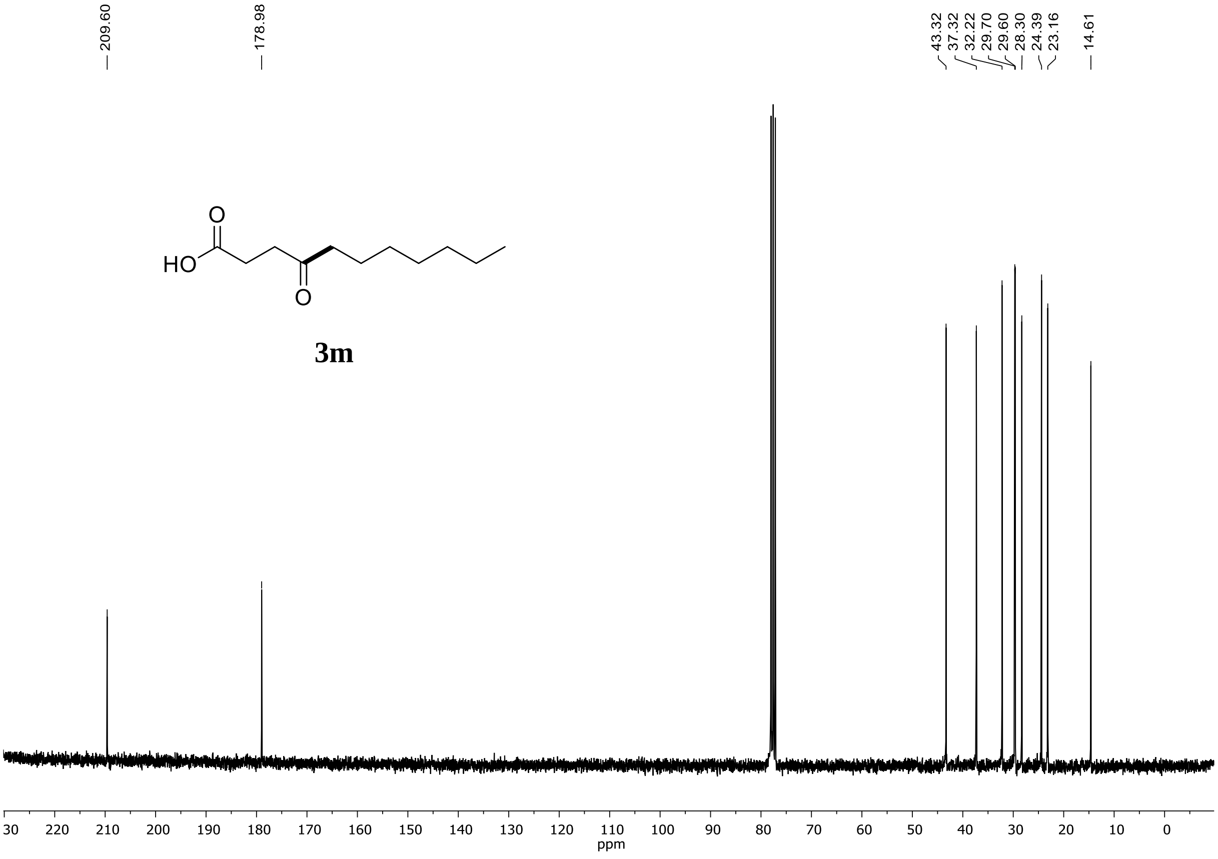

4-oxododecanoic acid (3n)

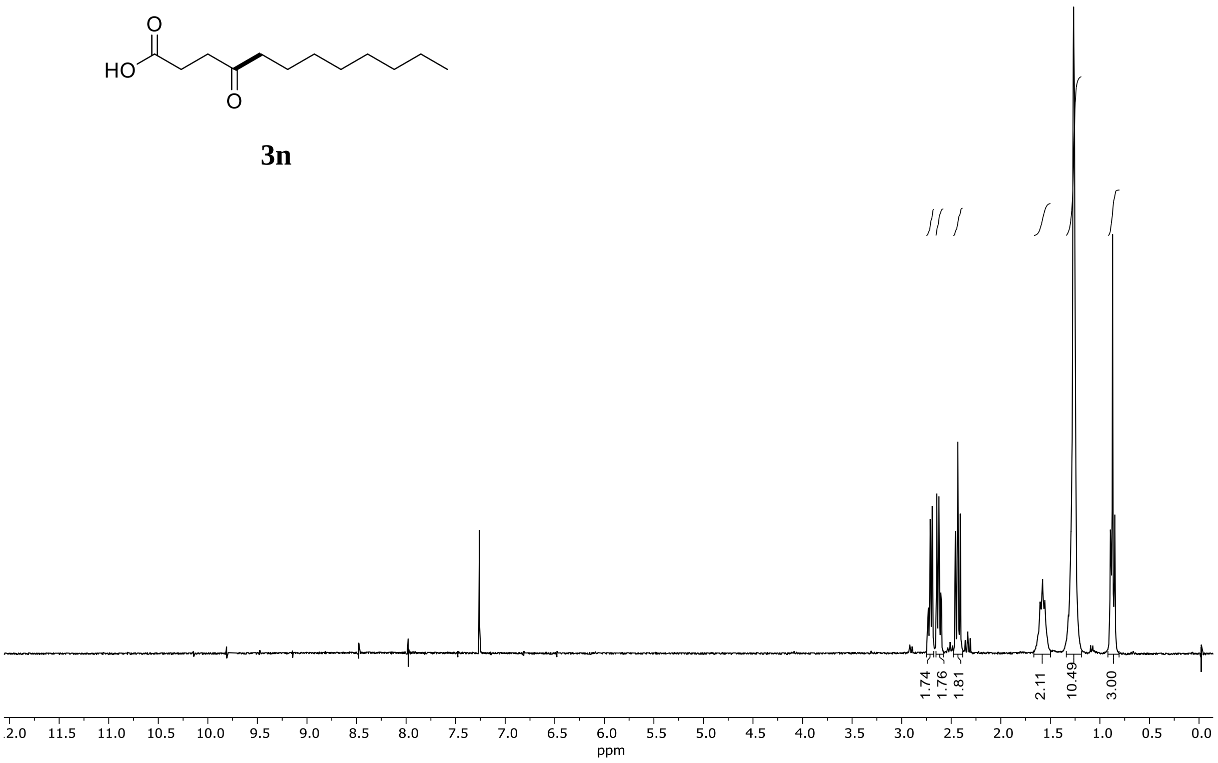

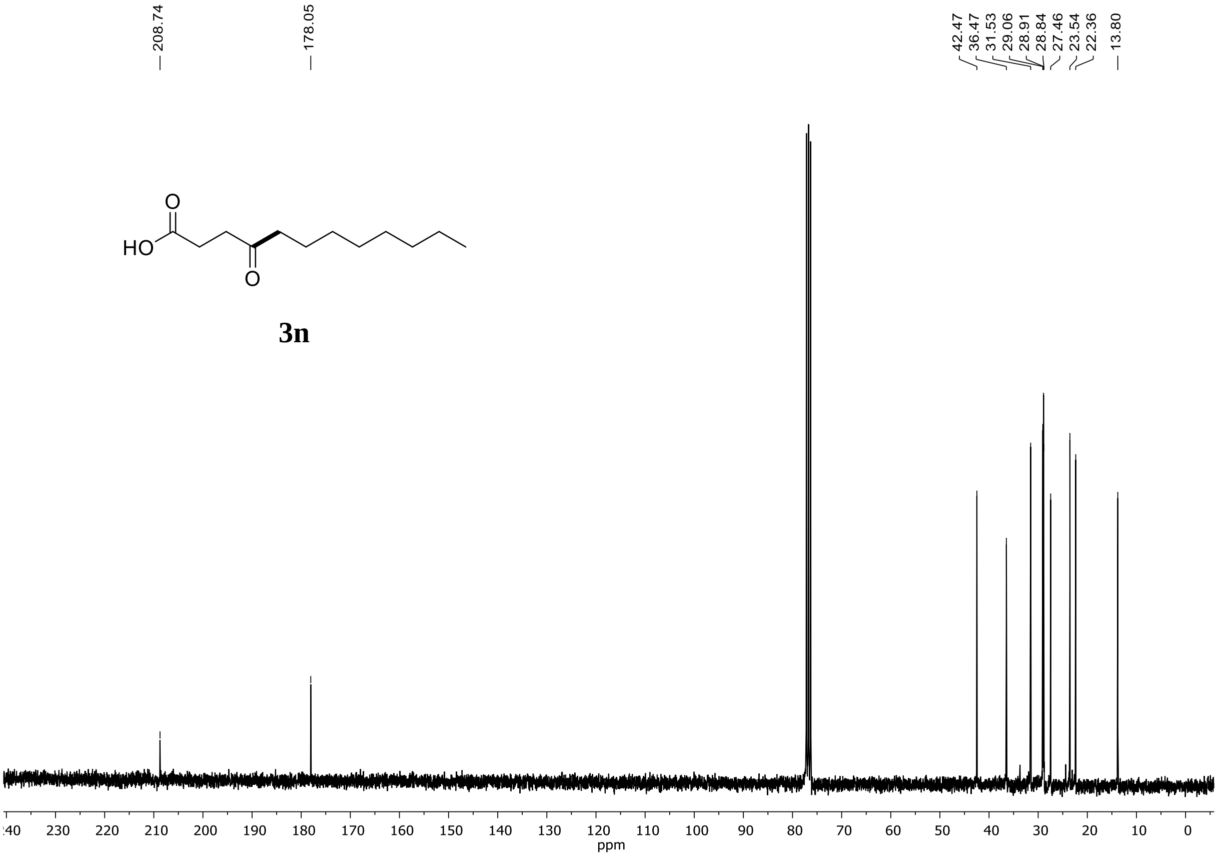

2-(4-cyano-2-methylbutan-2-yl)succinic acid (3o)

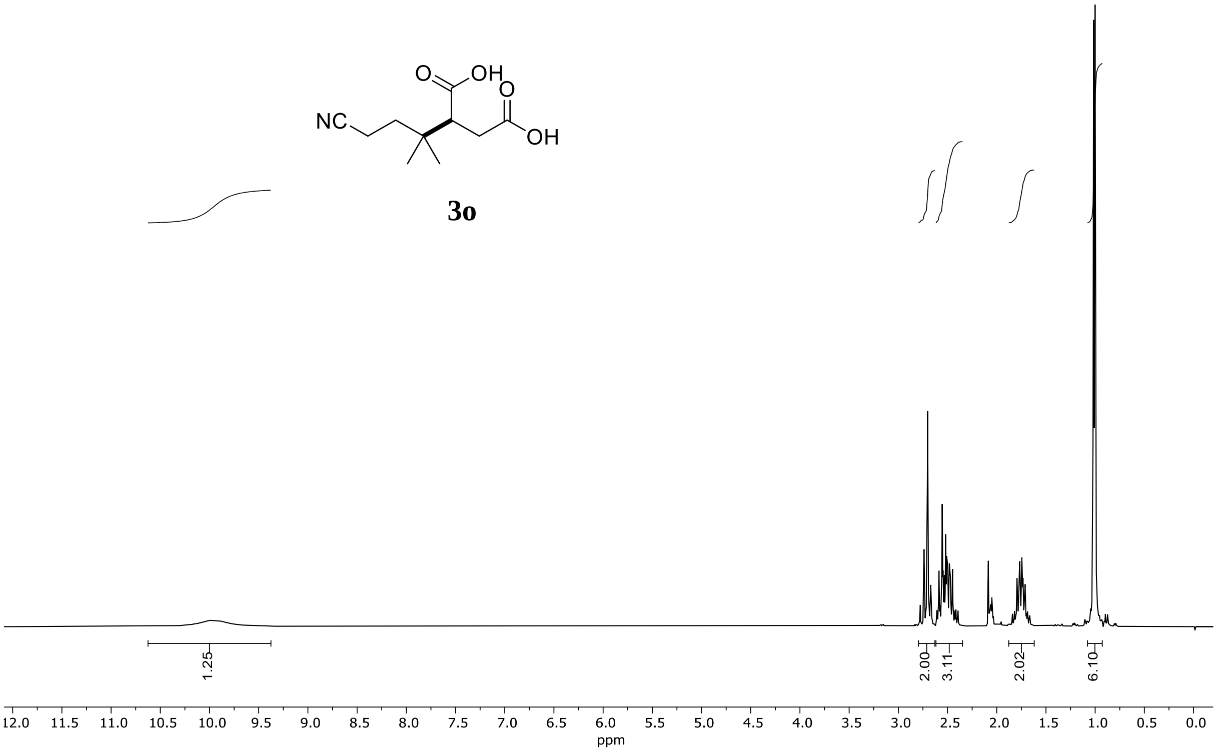

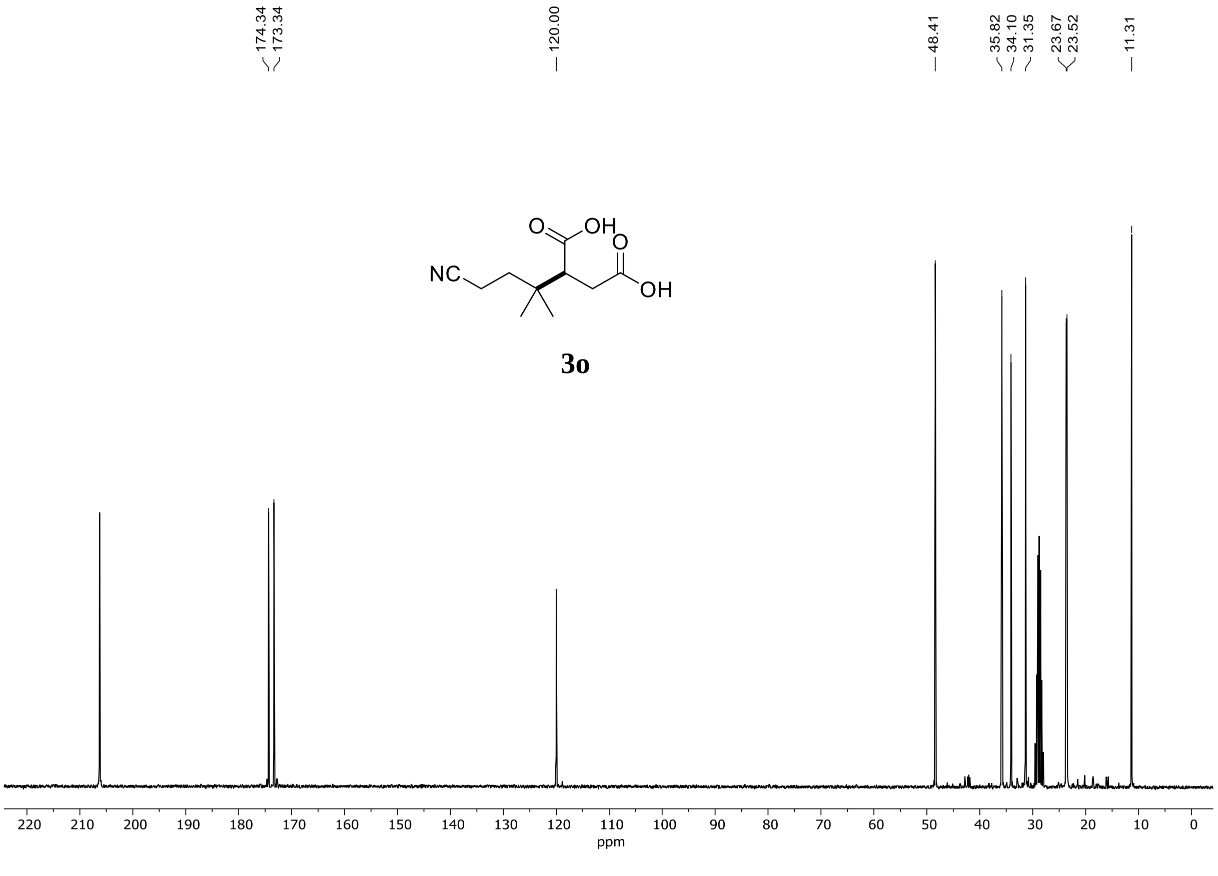

2-(3-oxocyclopentyl)succinic acid (3p)

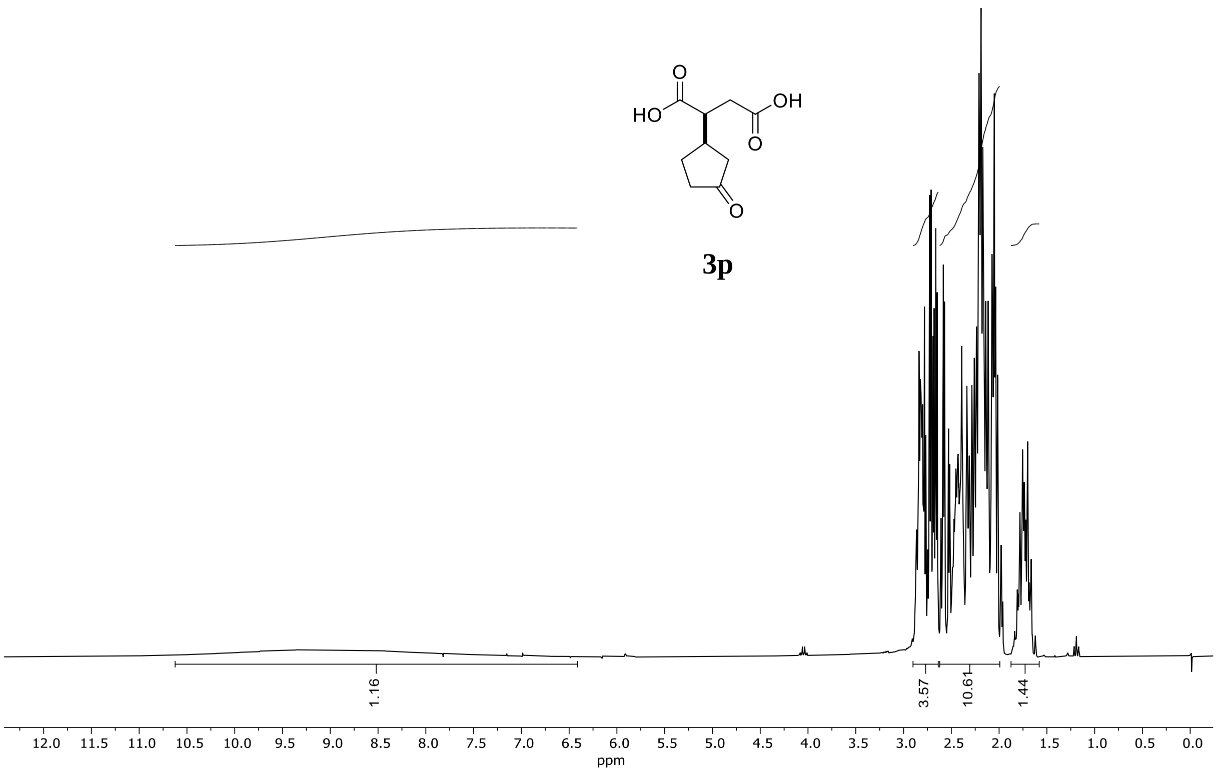

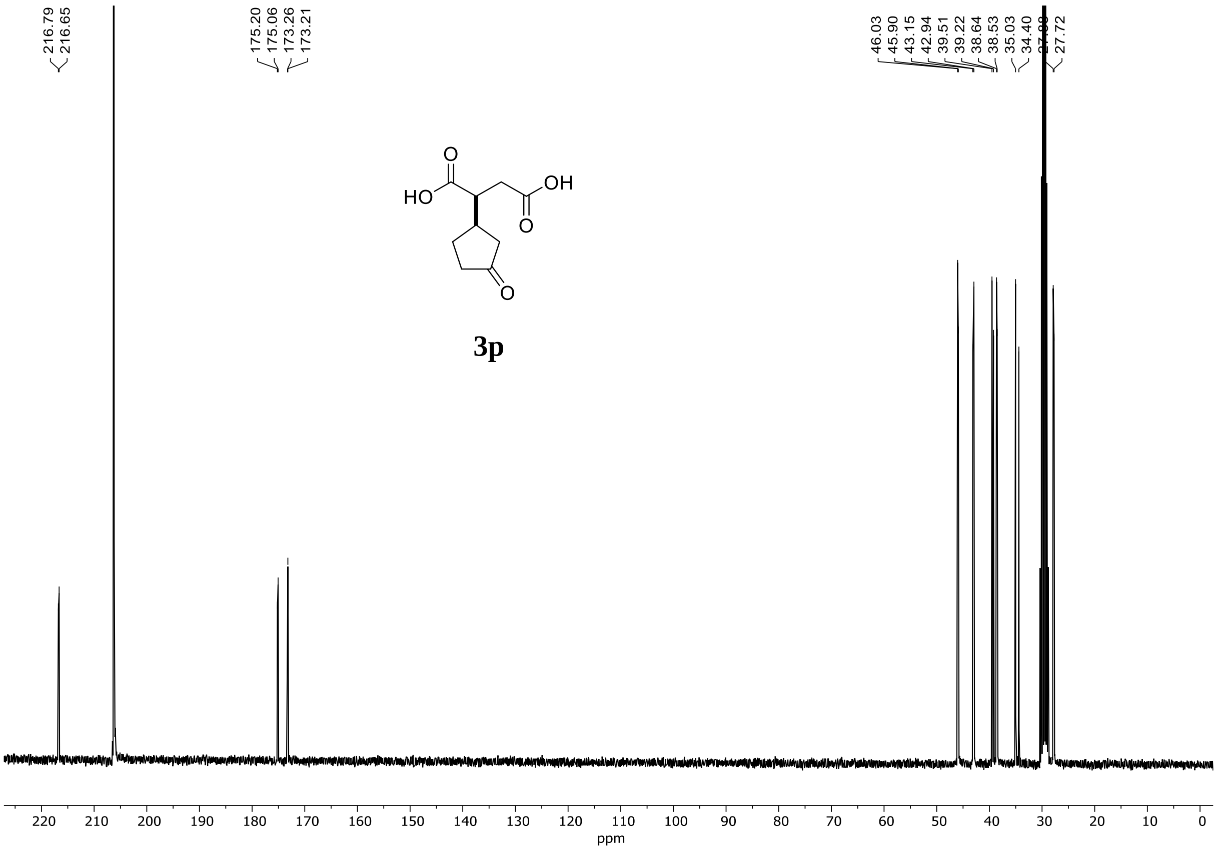

2-(1,3-benzodioxol-2-yl)succinic acid (3q)

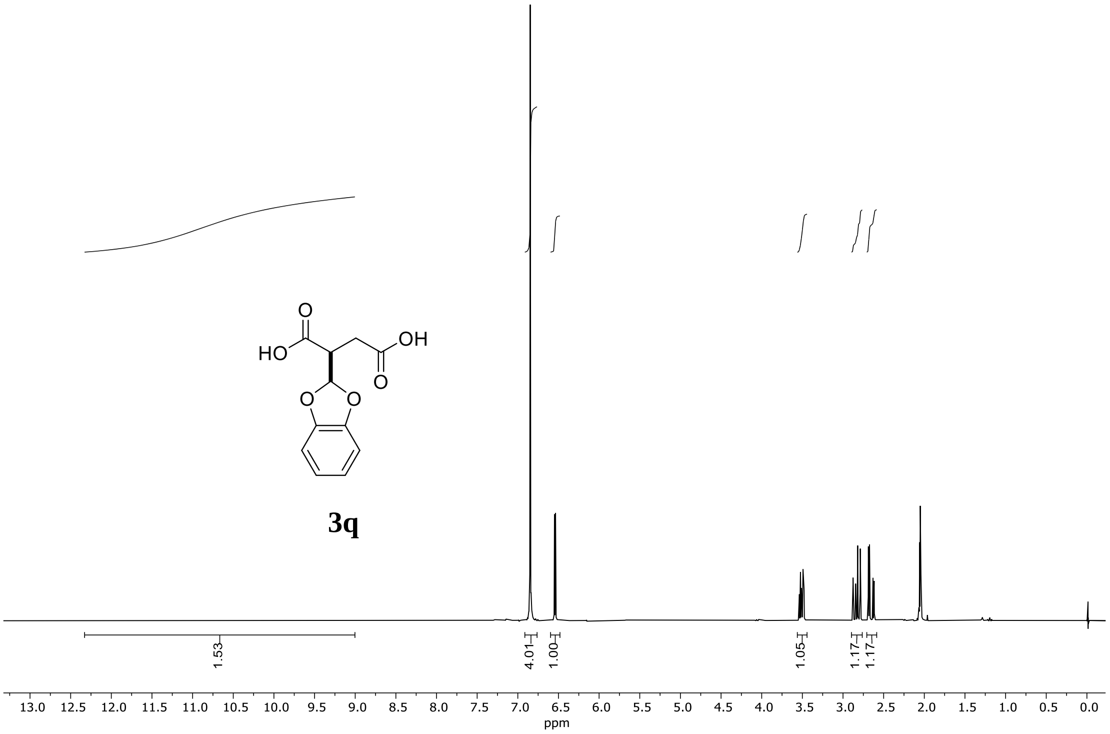

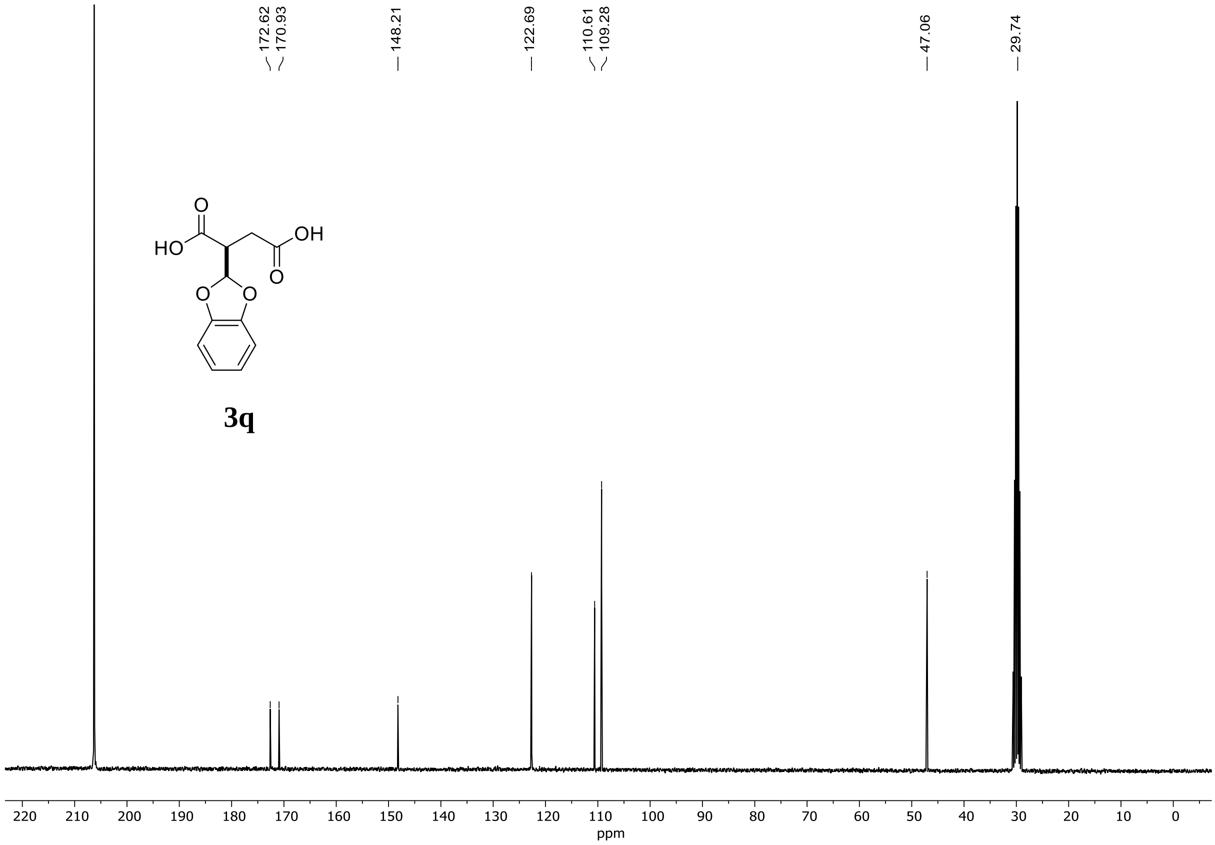

2-cyclopentylsuccinic acid (3r)

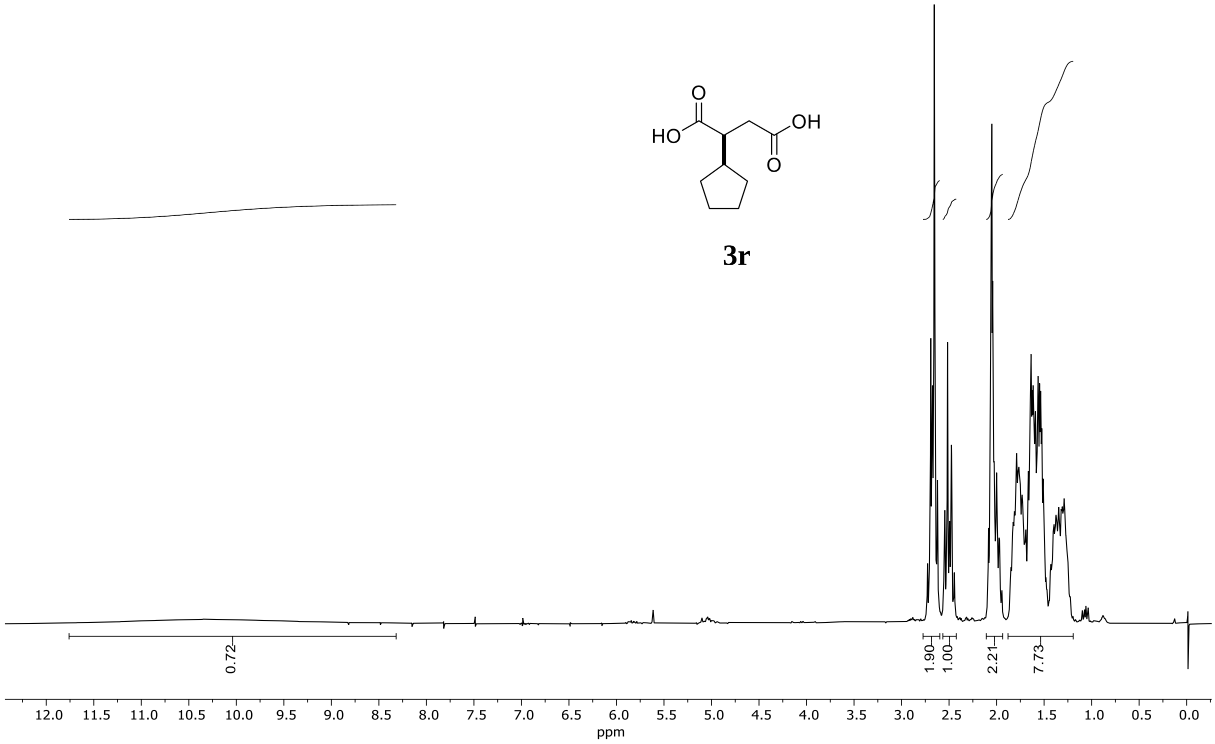

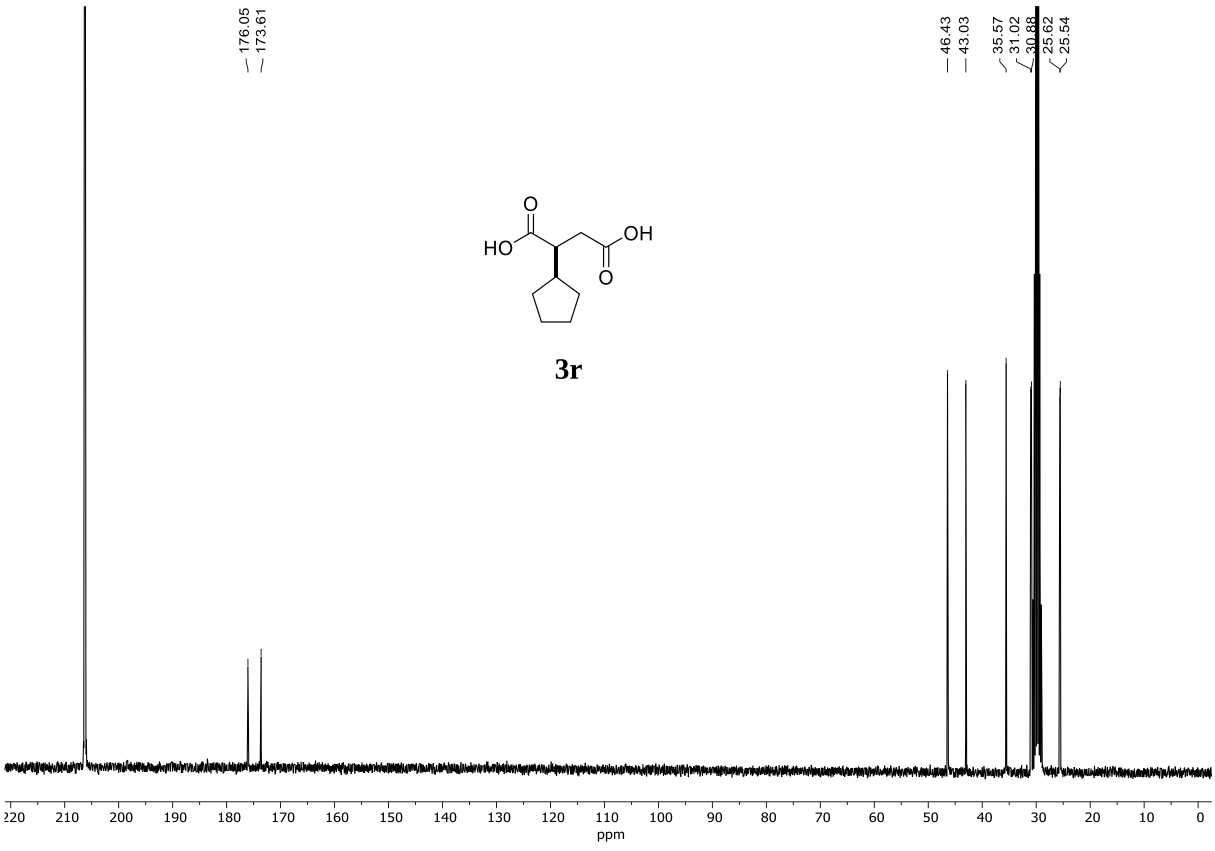

2-cyclohexylsuccinic acid (3s)

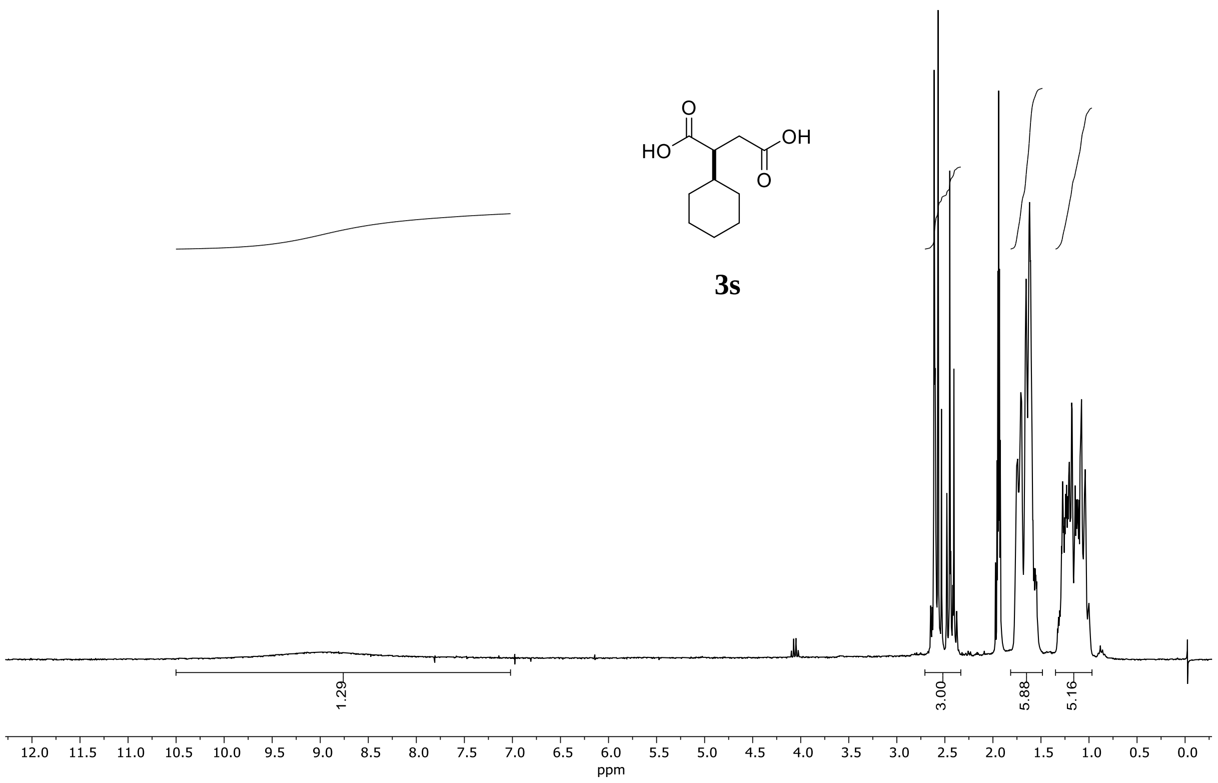

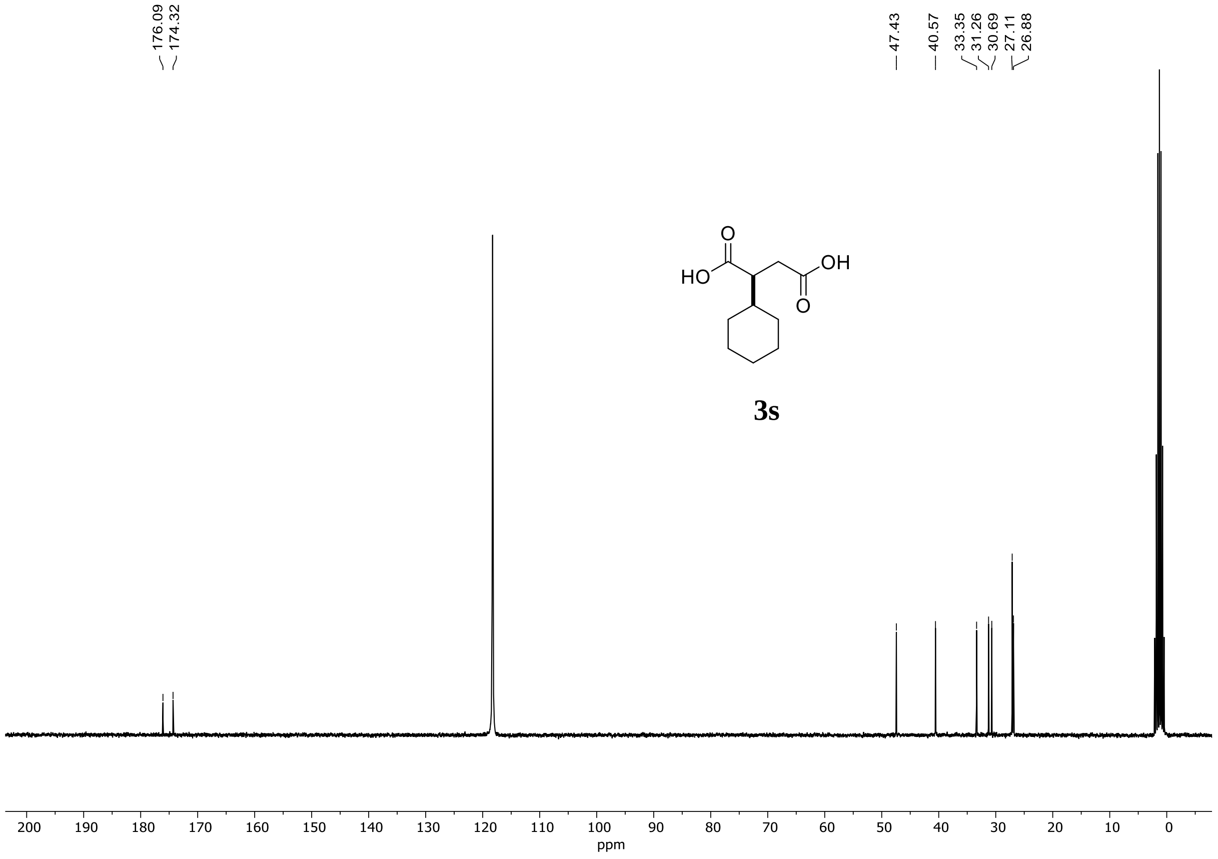

2-cycloheptylsuccinic acid (3t)

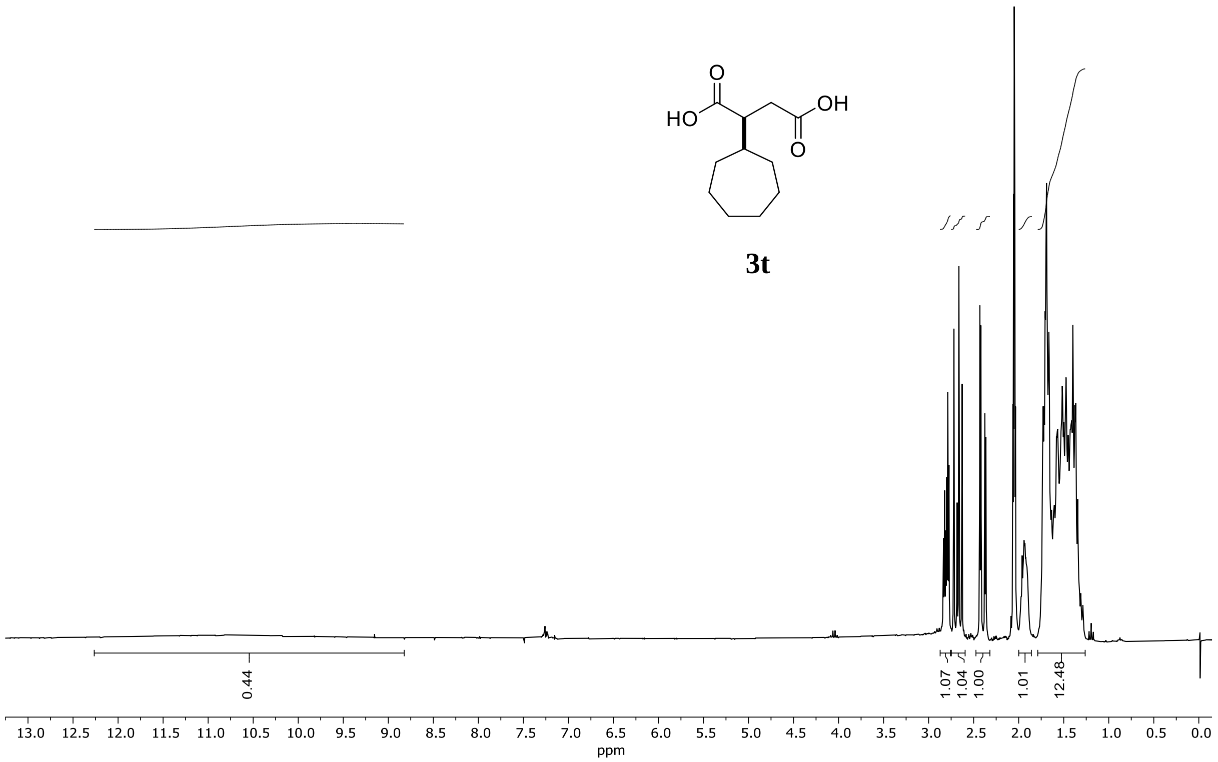

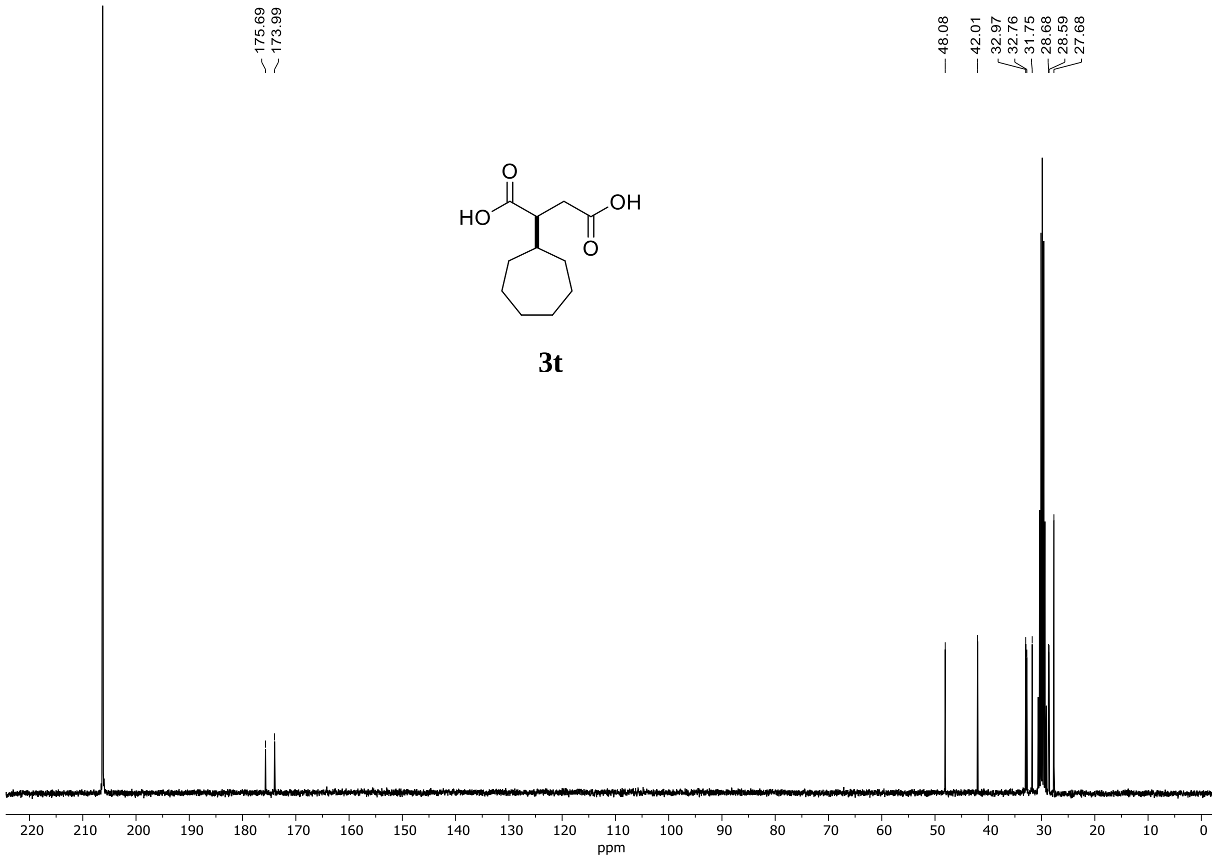

2-cyclooctylsuccinic acid (3u)

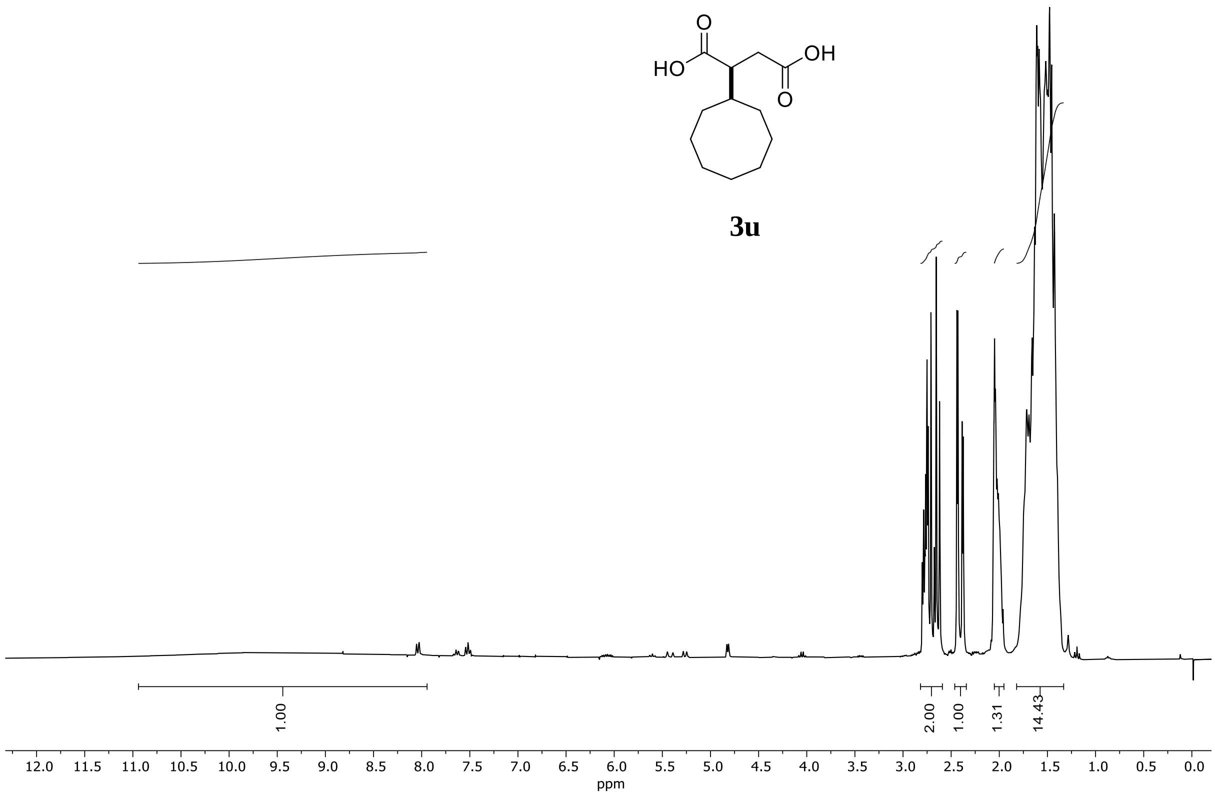

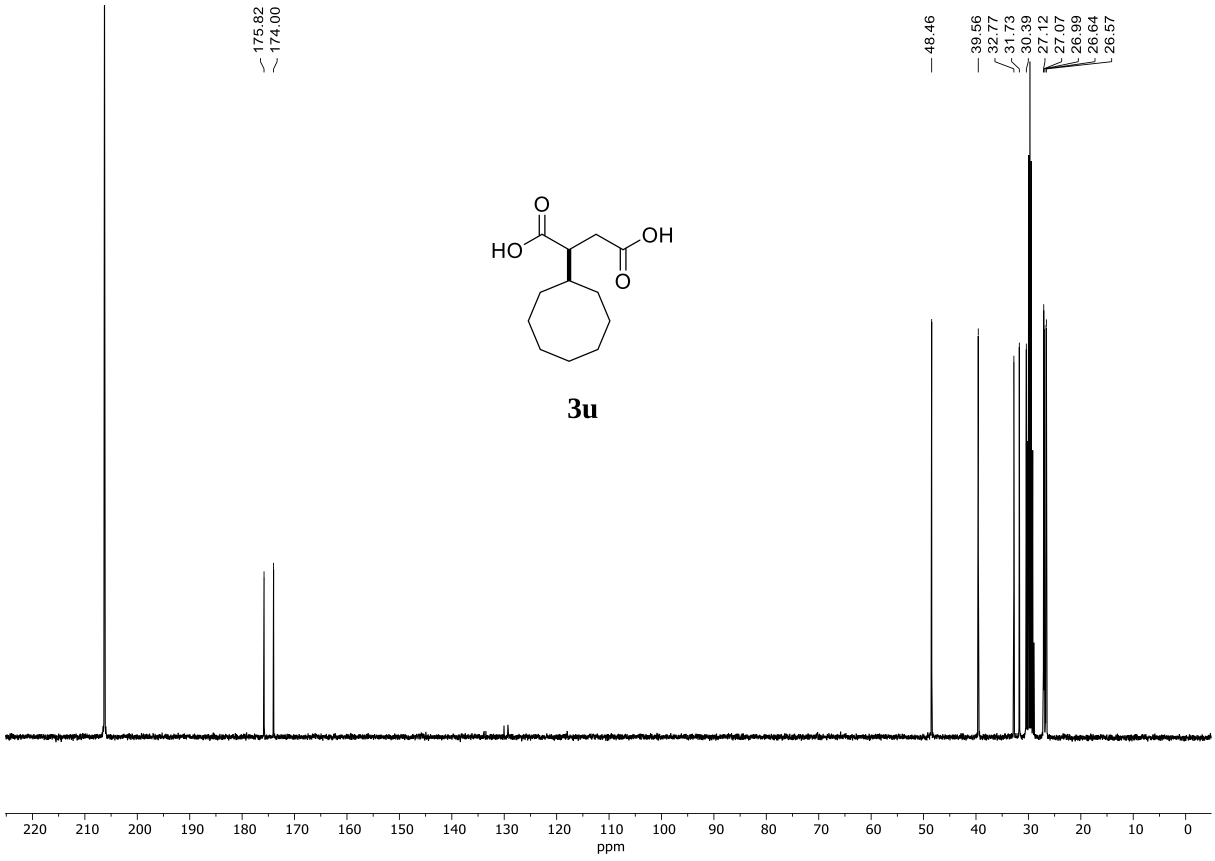

2-(2,3-dimethylbutan-2-yl)succinic acid (3v)

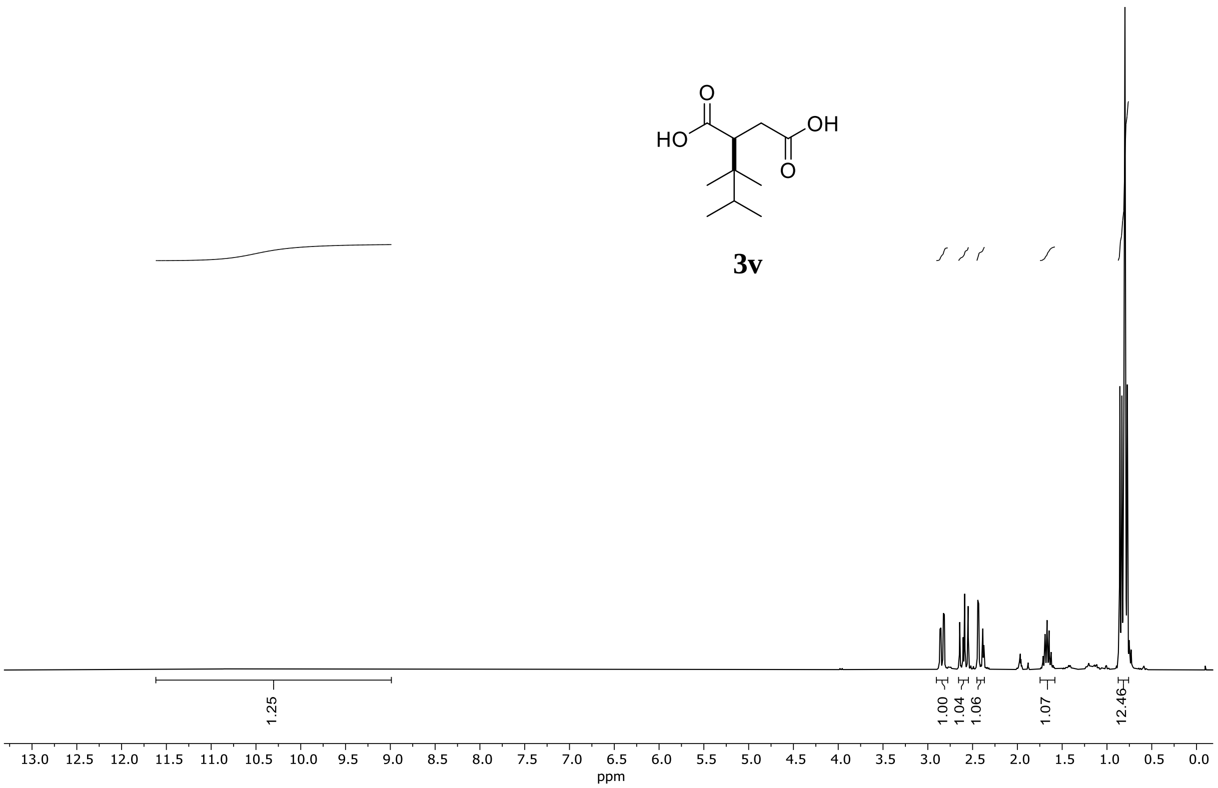

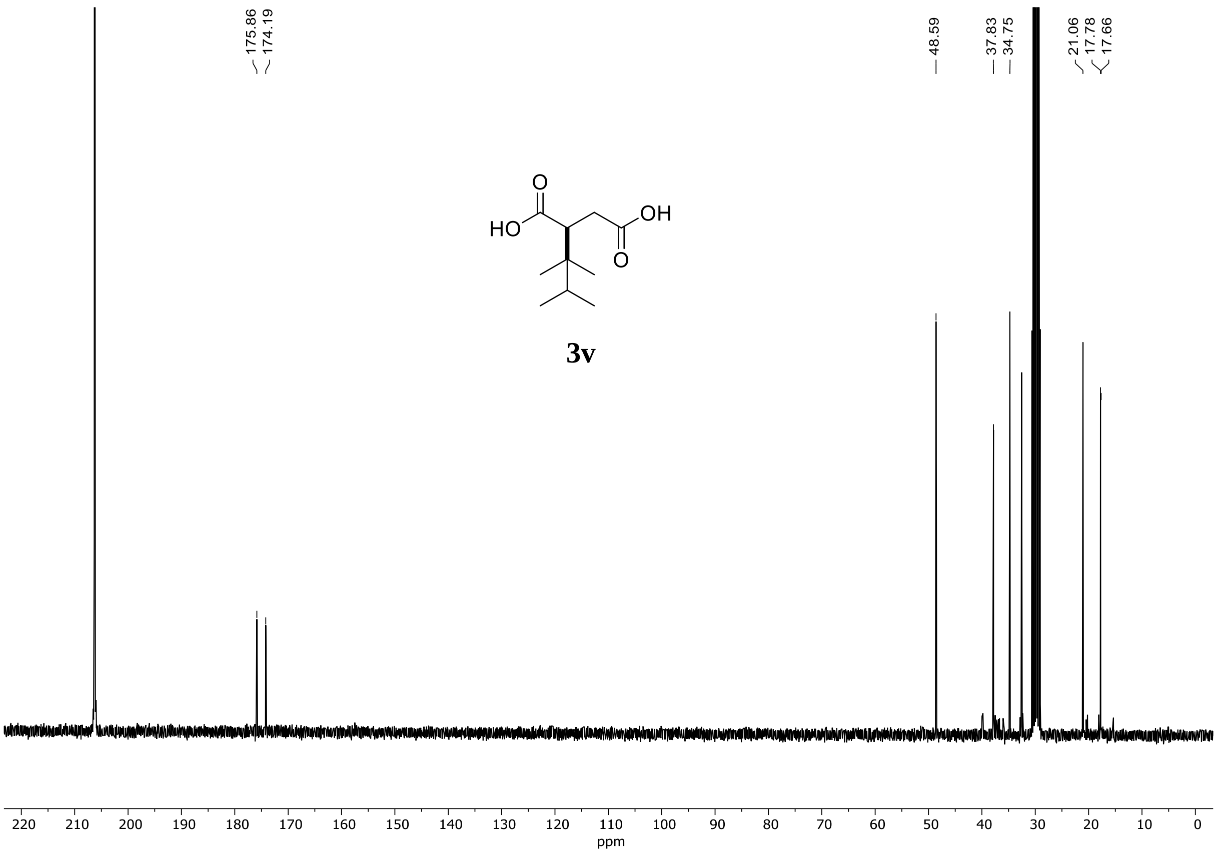

2-methyl-4-oxo-4-phenylbutanoic acid (4a)

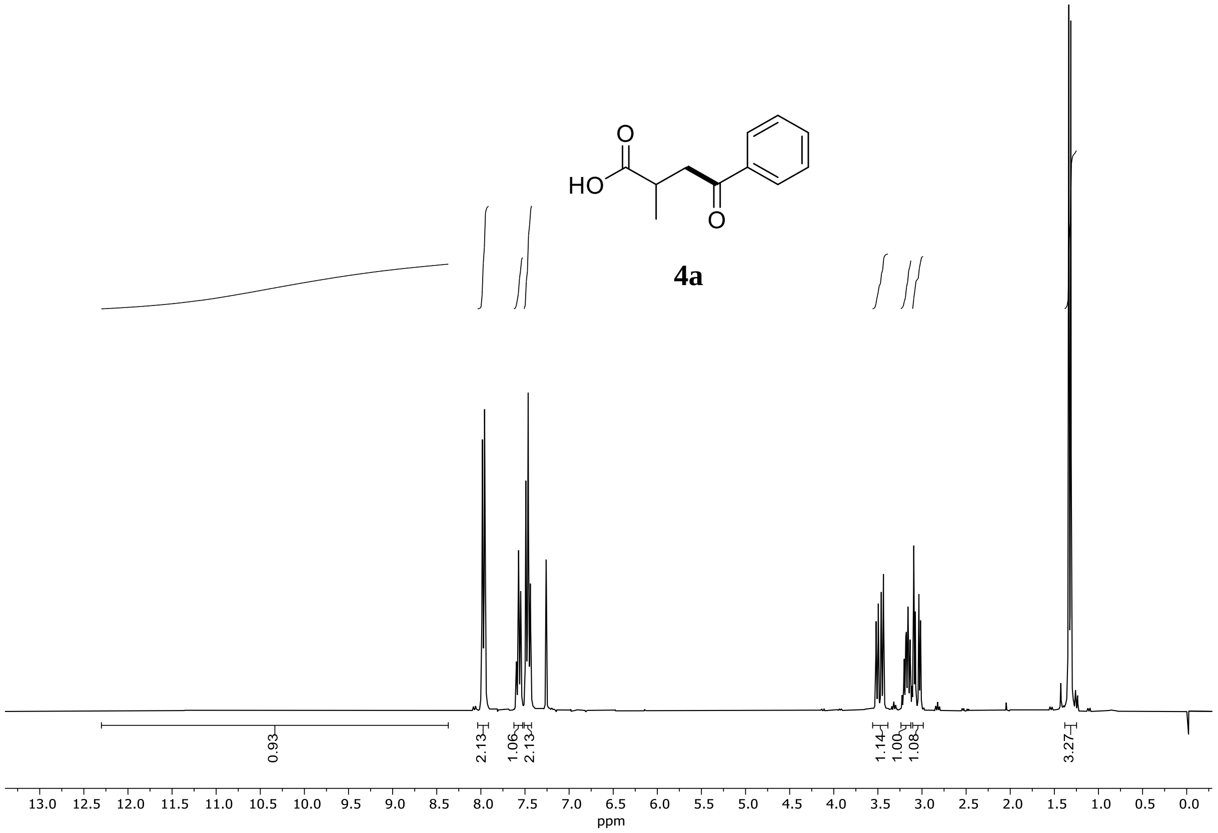

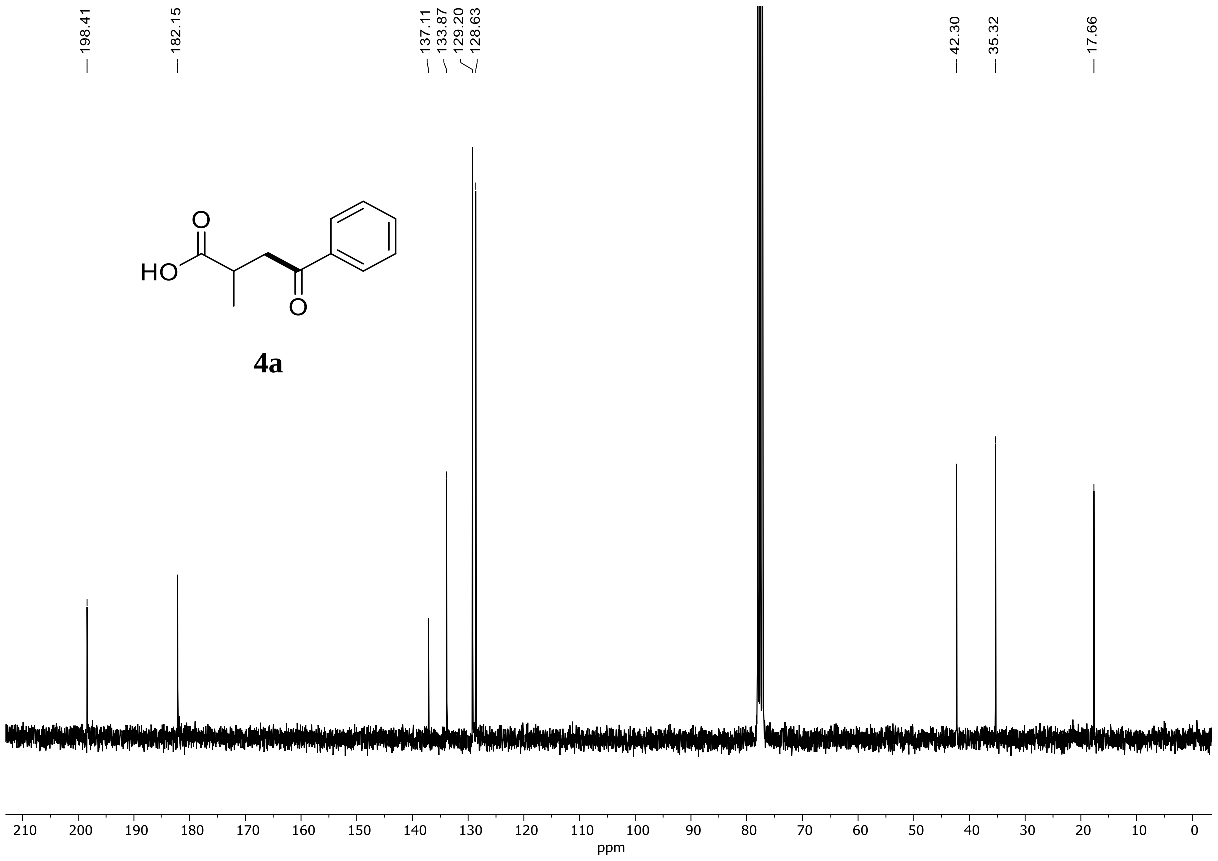

4-(4-methoxyphenyl)-2-methyl-4-oxobutanoic acid (4b)

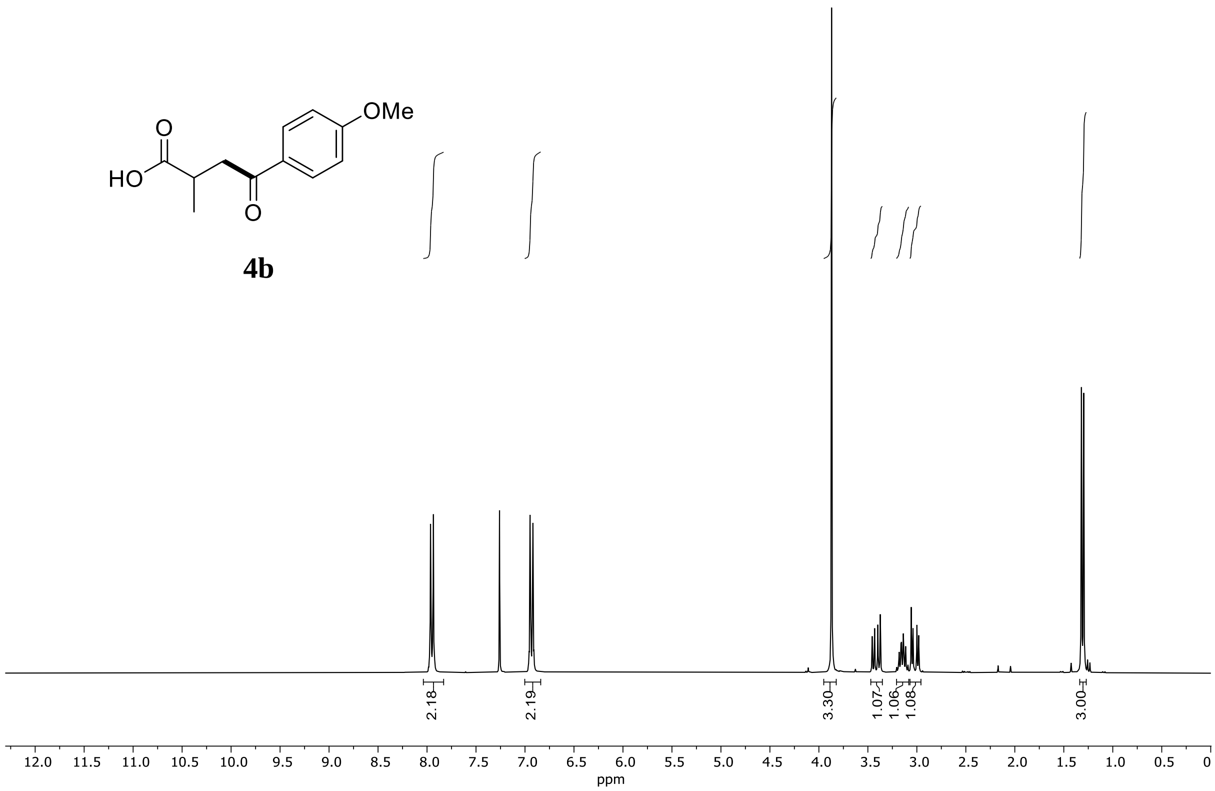

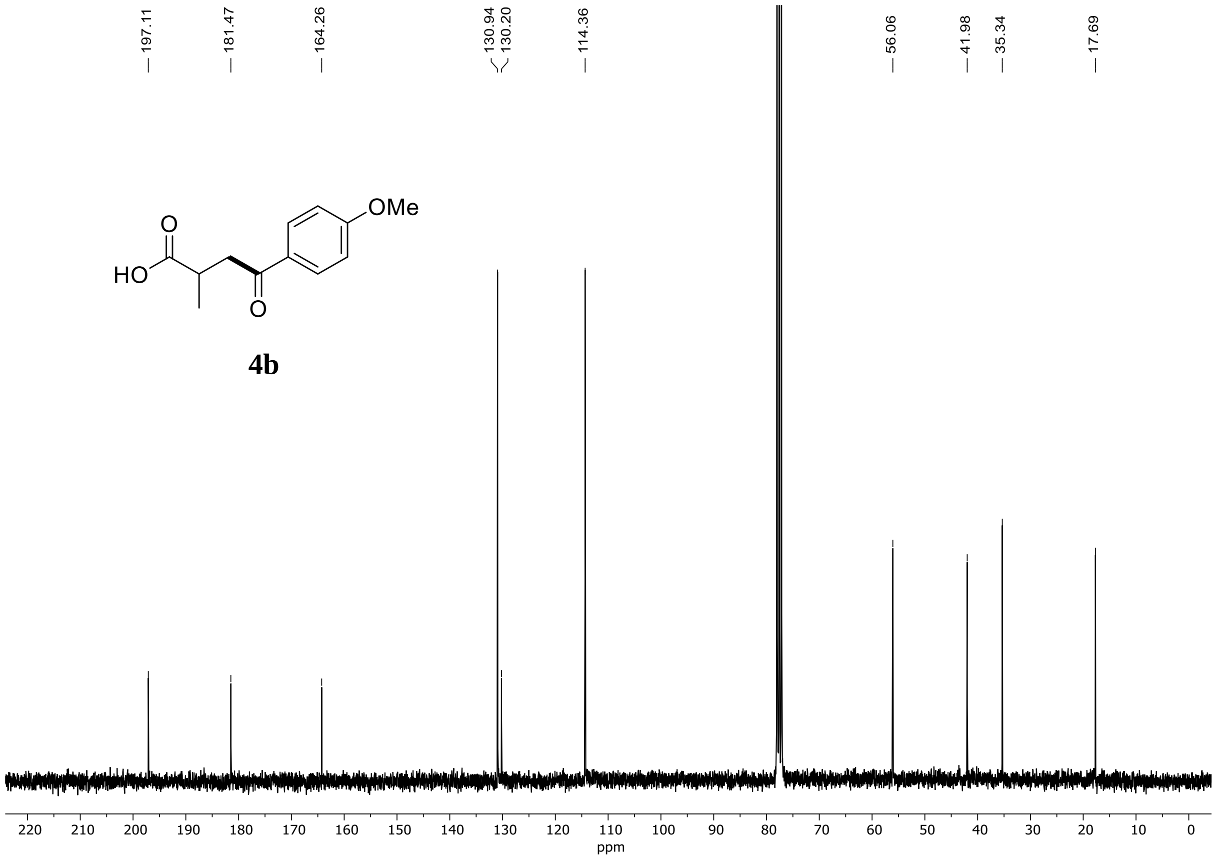

4-(4-chlorophenyl)-2-methyl-4-oxobutanoic acid (4c)

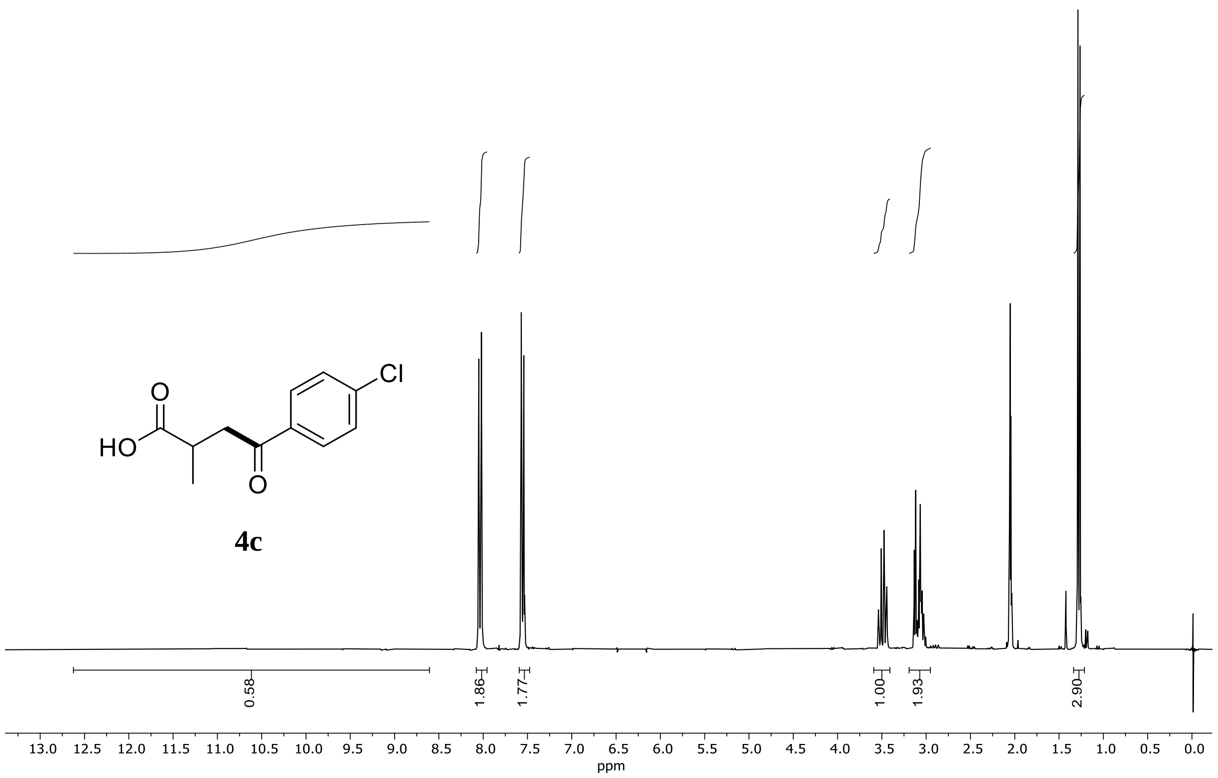

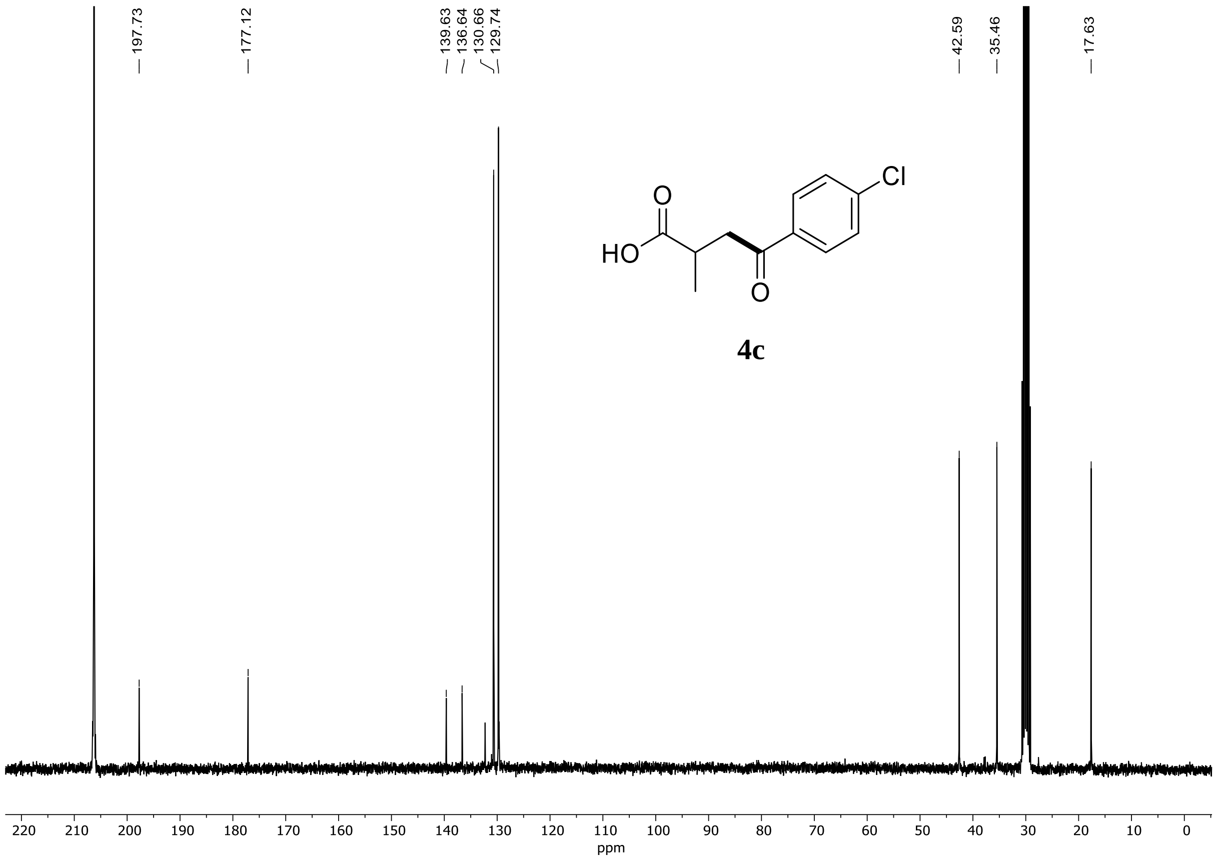

2-methyl-4-oxo-4-(3,4,5-trimethoxyphenyl)butanoic acid (4j)

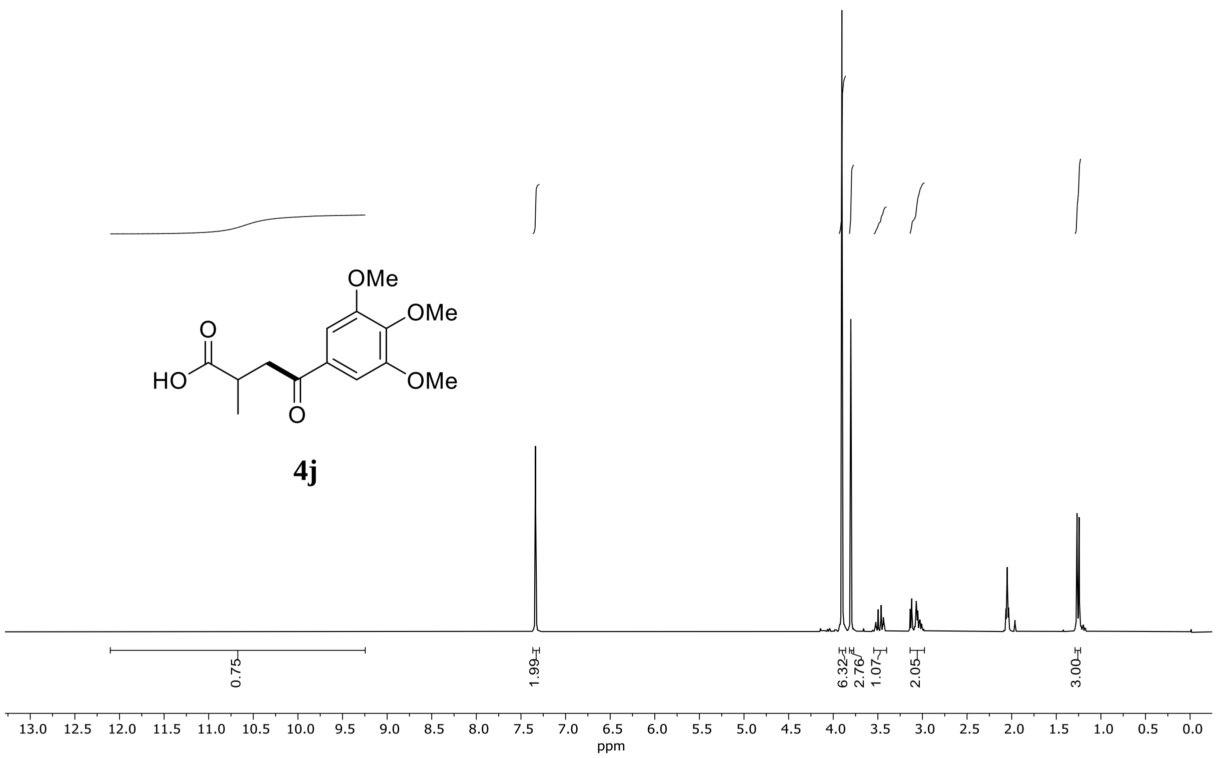

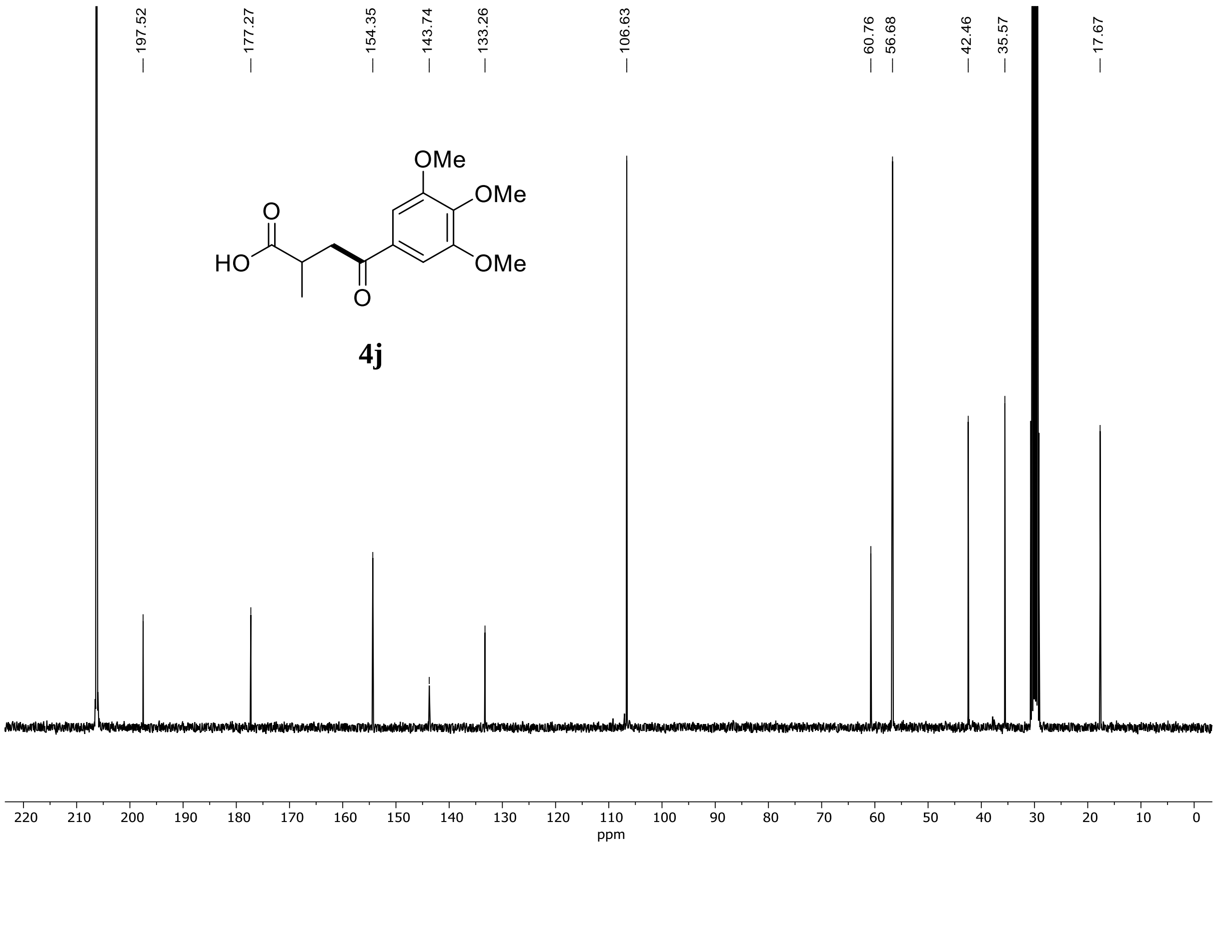

Reaction between hydrocinnamaldehyde and citraconic acid (mixture of 4k/4k')

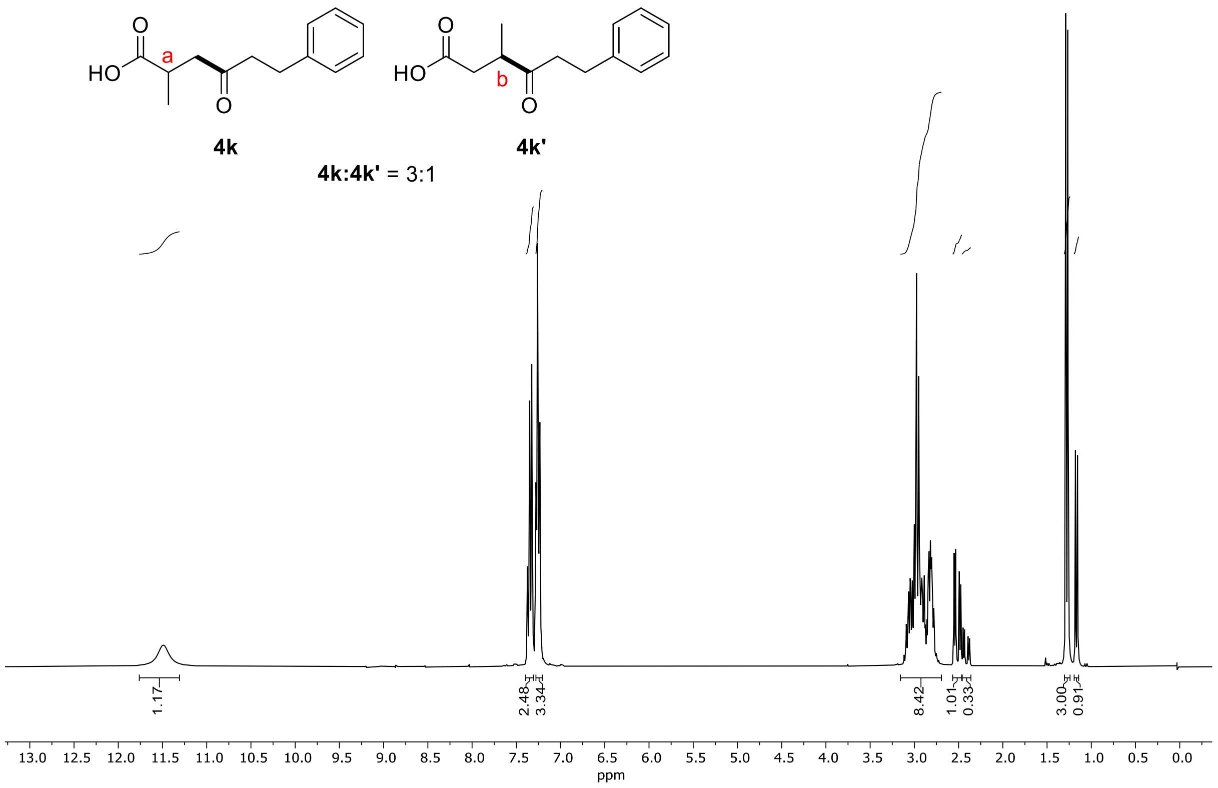

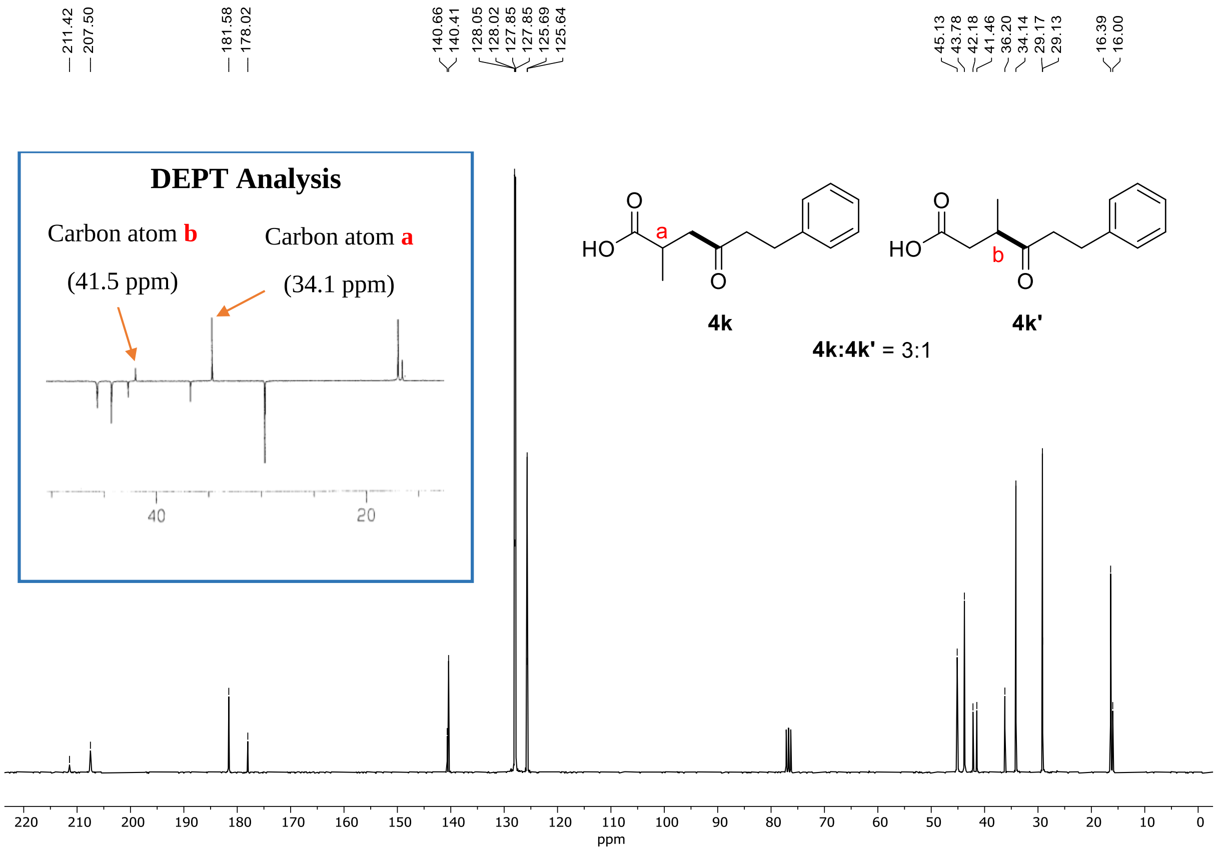

2-(4-cyano-2-methylbutan-2-yl)-3-methylsuccinic acid (4o)

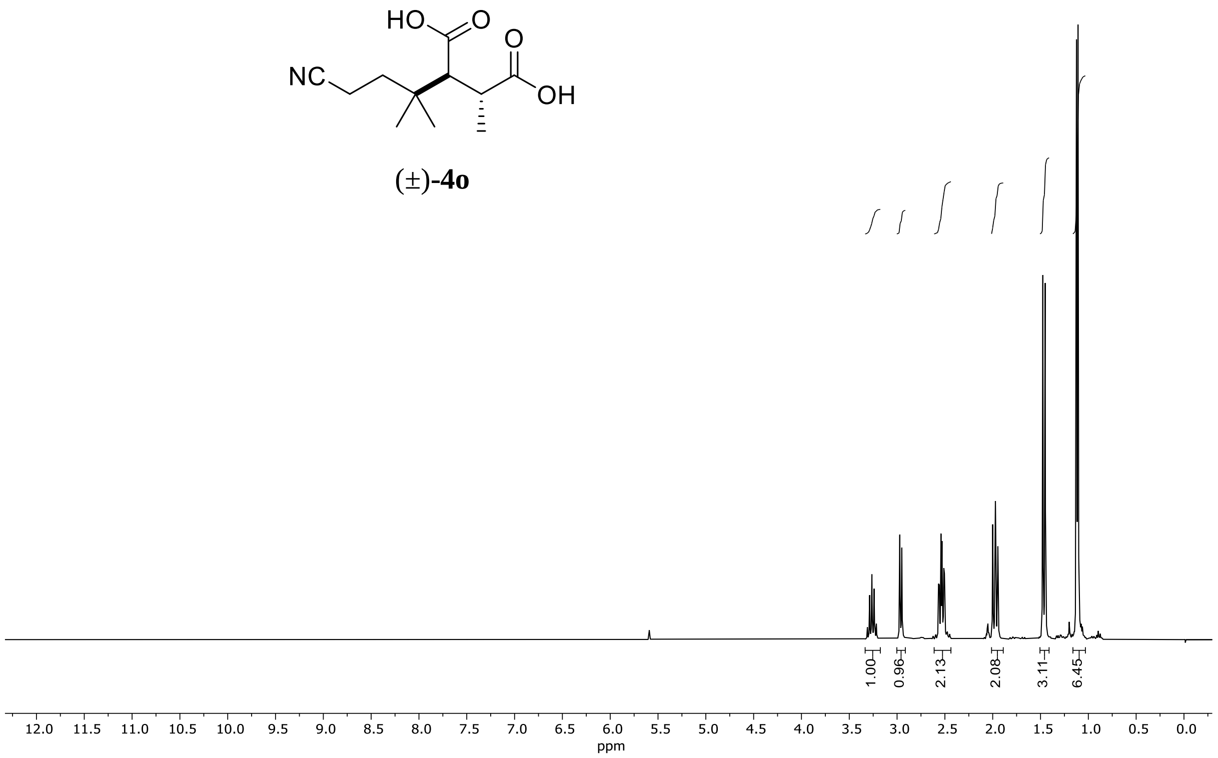

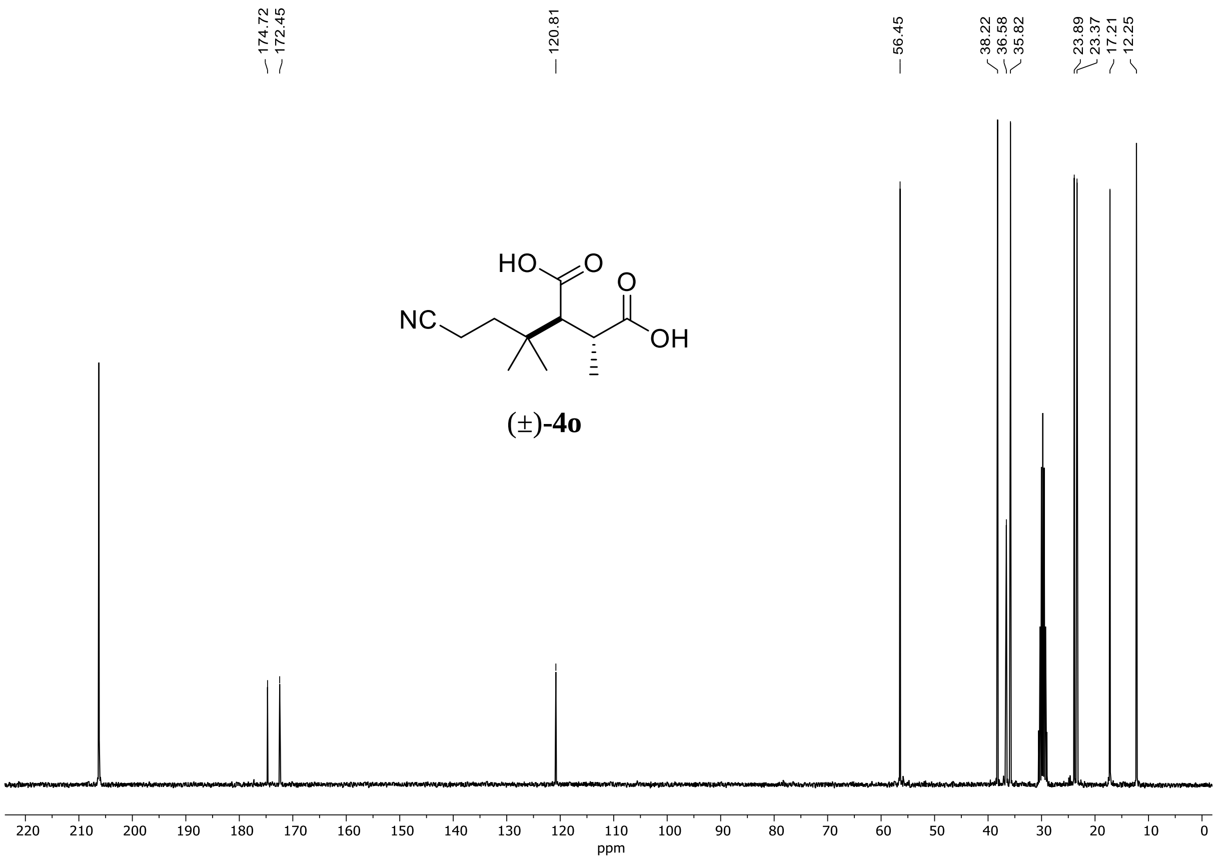

2-cyclopentyl-3-methylsuccinic acid (4r)

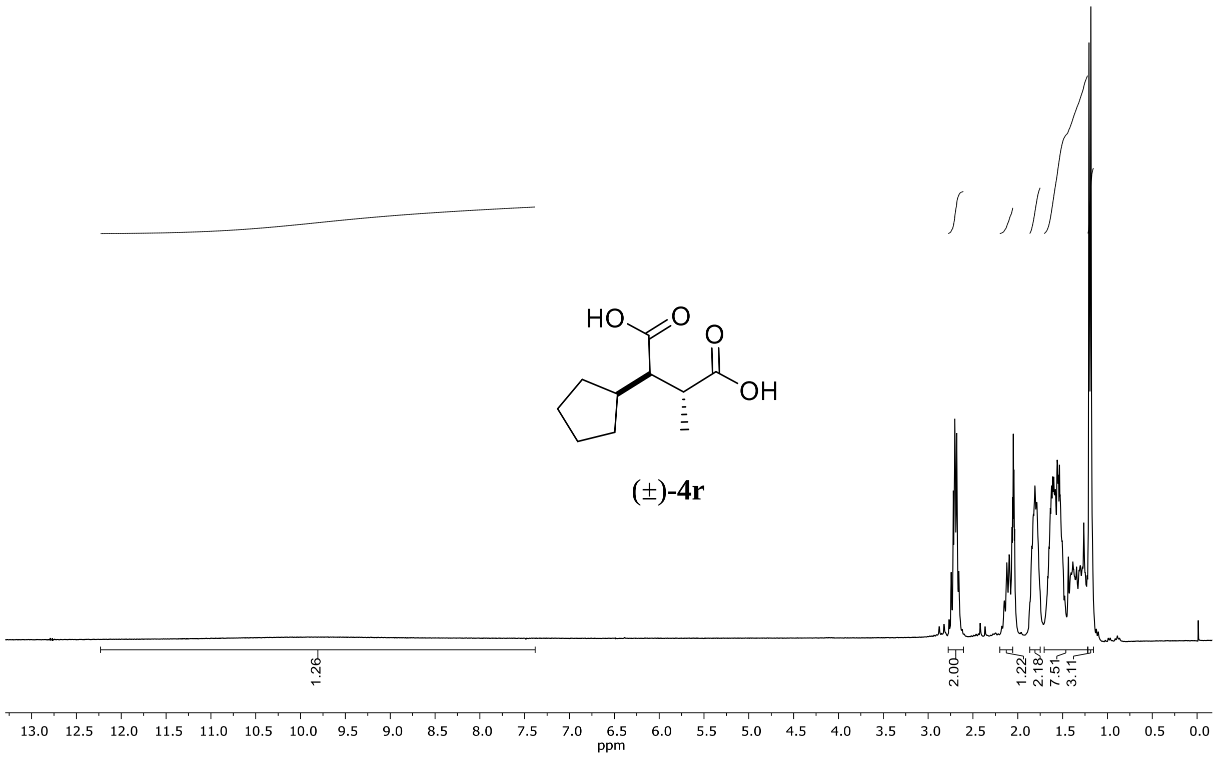

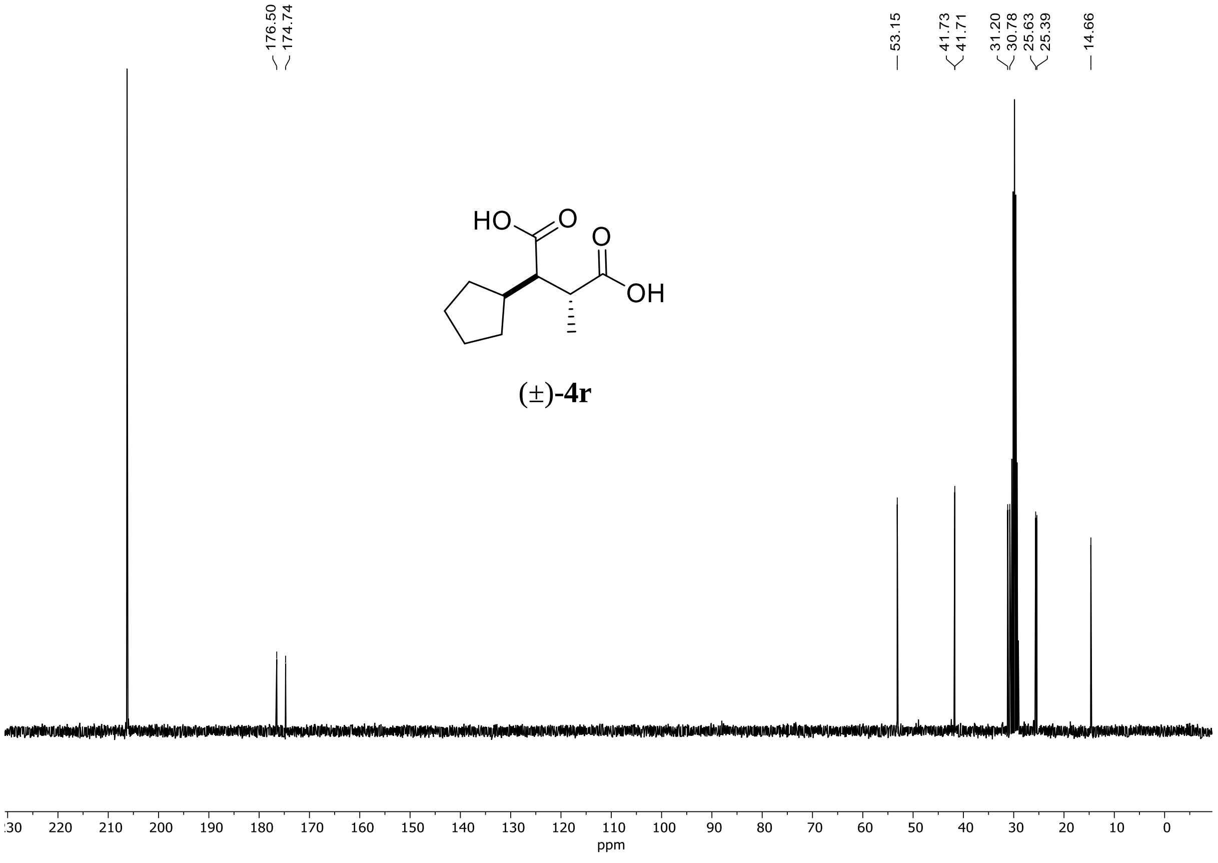

2-cyclohexyl-3-methylsuccinic acid (4s)

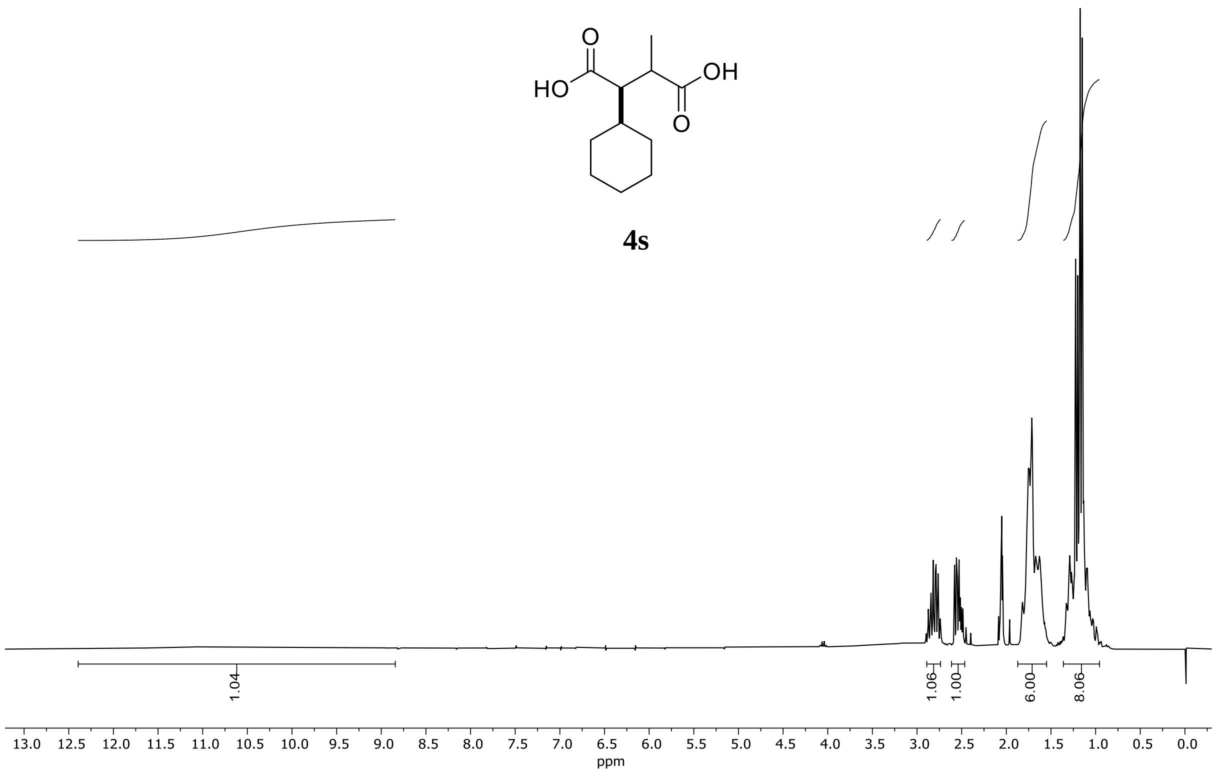

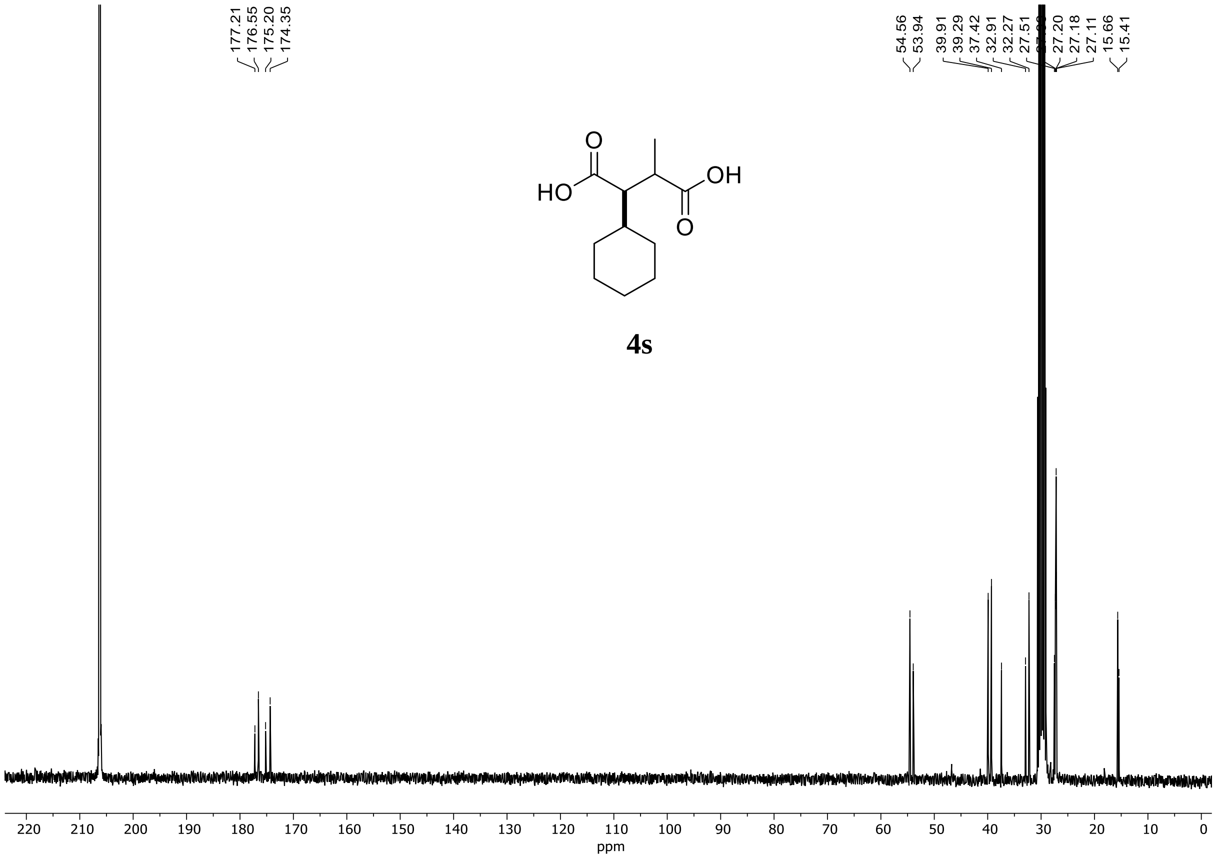

2-cycloheptyl-3-methylsuccinic acid (4t)

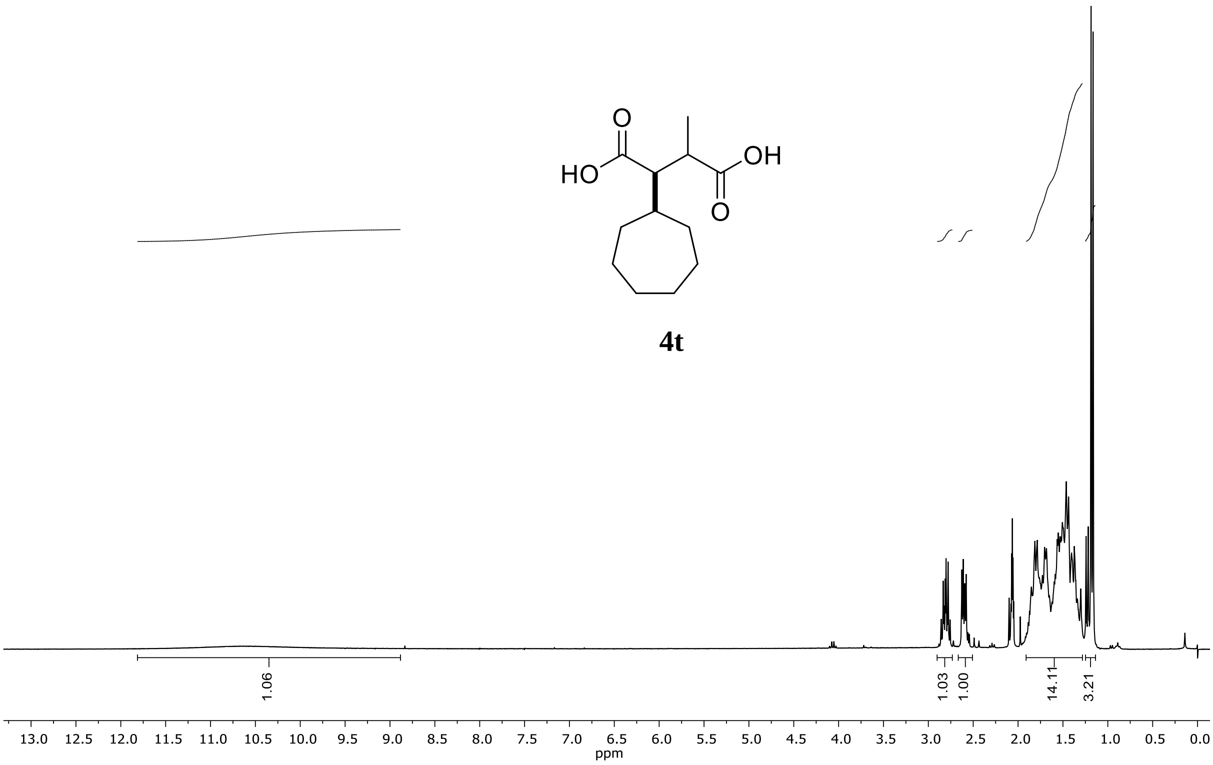

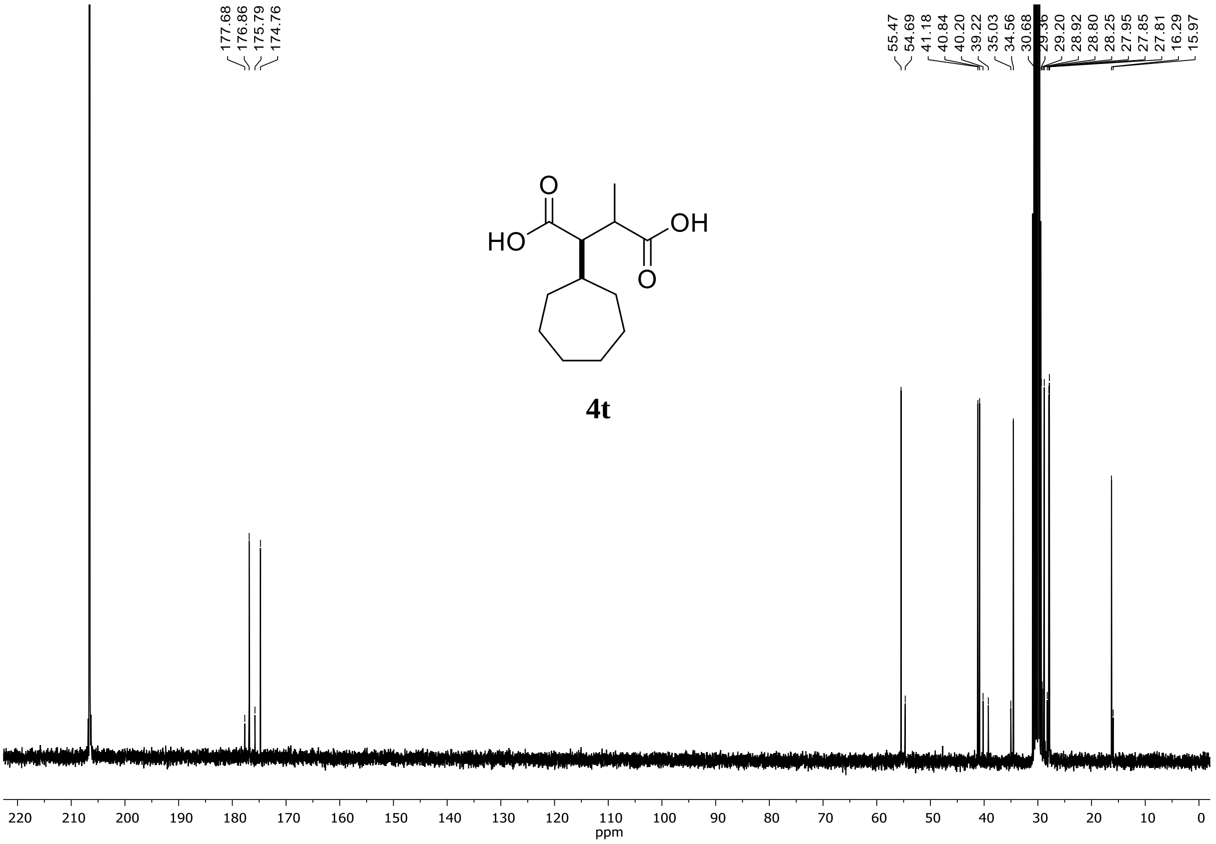

2-cyclooctyl-3-methylsuccinic acid (4u)

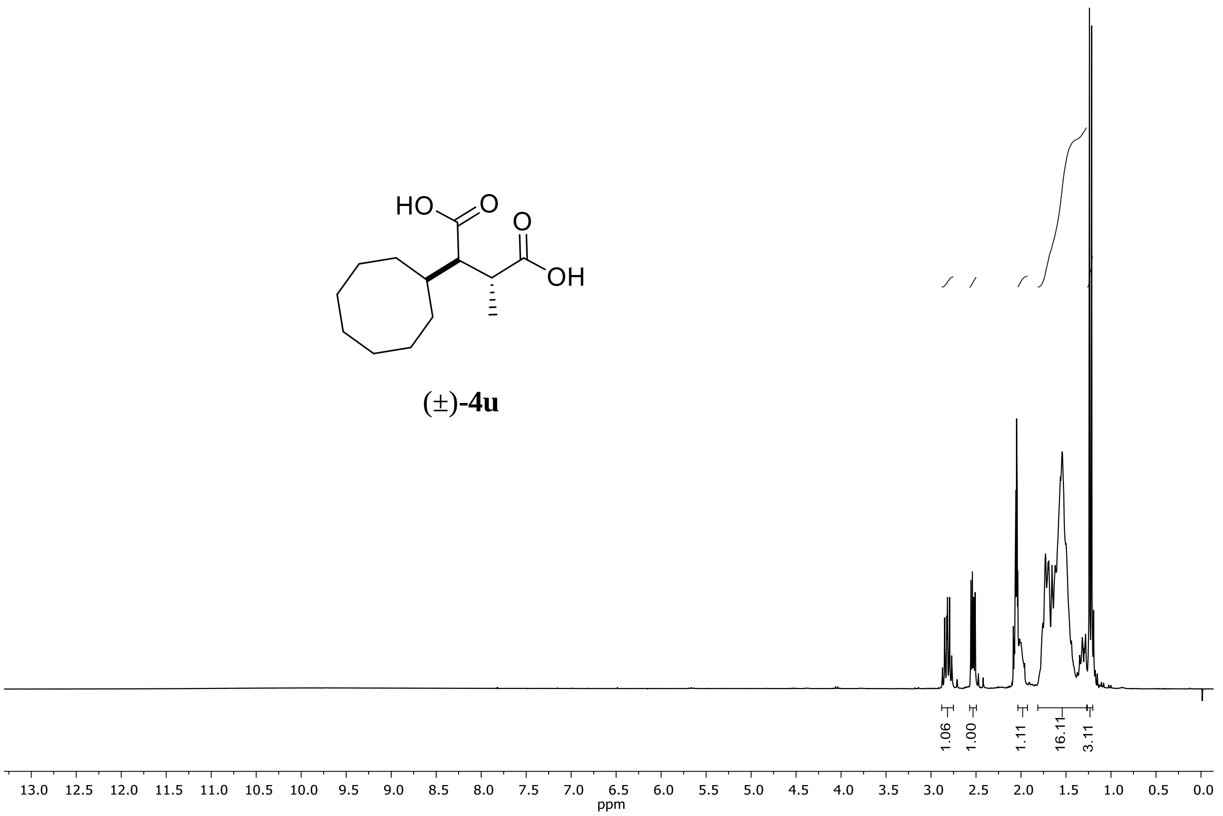

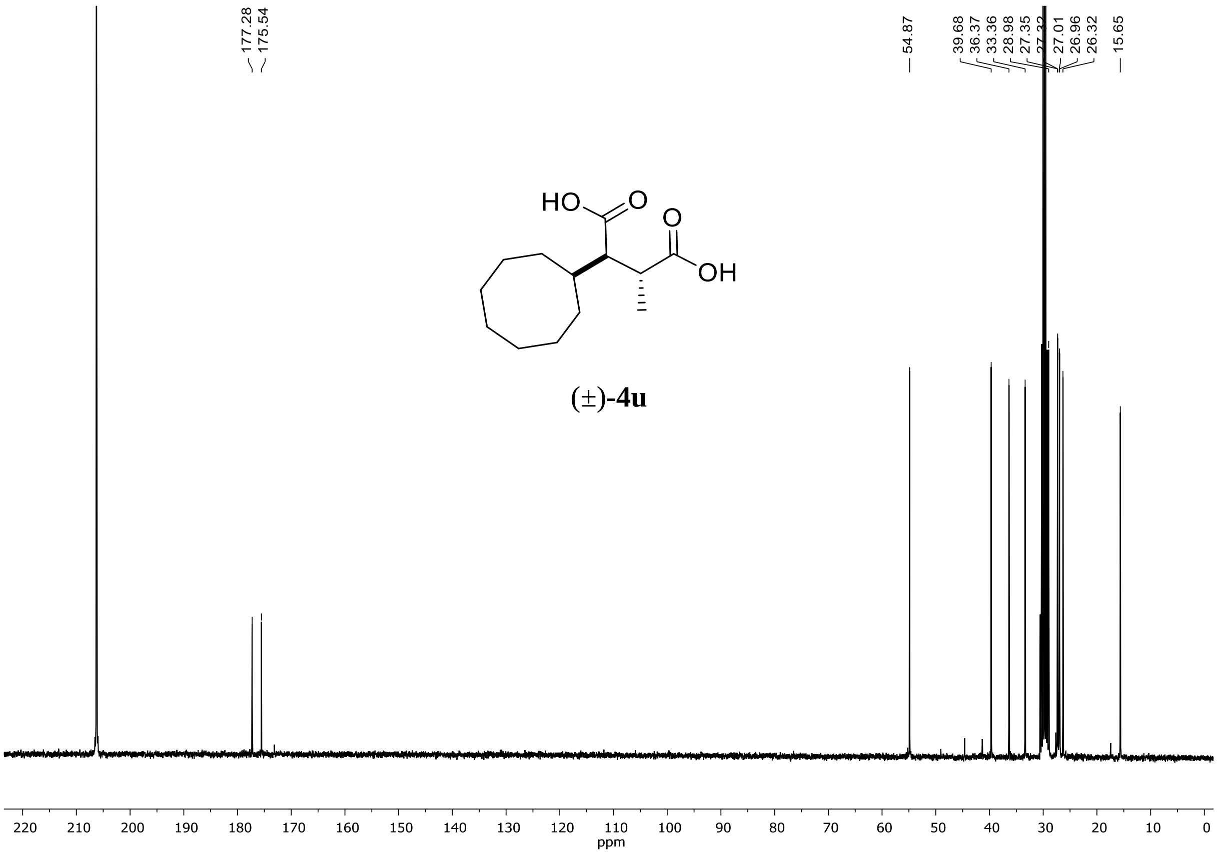

Supplement: Supplementary file 1 — Supporting Information [file CSSC-15-0-s001.pdf]
